# Supplementary material for: From “wading through treacle” to “making haste slowly”: A comprehensive yet parsimonious model of drivers and challenges to implementing patient data sharing projects based on an EPaCCS evaluation and four pre-existing literature reviews
Source: PLOS Digit Health. 2024 Apr 1;3(4):e0000470. doi: 10.1371/journal.pdig.0000470 (PMC10984410; doi:10.1371/journal.pdig.0000470)
Supplement: S1 File — (DOCX) [file pdig.0000470.s001.docx]

**S1 File: Further methodological information**

1. **Further information on the design of the broader study, “Prepared to Share?”** (https://www.phpc.cam.ac.uk/pcu/research/research-projects-list/prepared-to-share/)
   1. **Aims and objectives:**

- Describe the data sharing tools used in the Cambridgeshire and Peterborough Clinical Commissioning Group (CCG) Project for Data Sharing in End of Life Care (the EoLC Data Sharing Project) and the broader configuration of processes, roles and structures which enable their functioning. Use this as a starting point in developing a generalisable framework for describing Electronic Palliative Care Coordination Systems (EPaCCS).
- Collect indicative data on the uptake of the EoLC Data Sharing Project, while supporting the development of its reporting functionalities (the EoLC Dashboard), including with a view to future research opportunities and needs.
- Following principles of a realist evaluation, articulate mechanisms sustaining current problems and precluding change (challenges to the EoLC Data Sharing Project development and implementation, reasons for its low uptake and low effectiveness) and mechanisms enabling change (drivers/ facilitators of the project development and implementation, reasons for its uptake and effectiveness), as well as contexts conducive to and precluding the firing of change mechanisms.
- Elicit the knowledge and attitudes of a broad range of stakeholders (health professionals, project developers, commissioners, patients, carers, etc.) to patient data sharing, at the end of life and more broadly, as potentially key elements of mechanisms precluding/ facilitating change.

These aims were somewhat different in their specifics (though not in overall direction) than the original study aims (protocol available from the authors). Three factors led to a number of modifications of the initial study aims, objectives and data collection approaches:

- The development and implementation of the EoLC Data Sharing Project was far more challenging, slower and less successful than expected. In addition, the final technical solution was quite different to the original vision (a ‘dispersed’ data sharing solution as opposed to a centralised register), which affected the availability of routinely collected data. As a result, the planned-for quantitative evaluation of the EoLC Data Sharing Project was far more ambitious than what was possible in the actual evaluation. We expanded the qualitative aspect of the evaluation.
- Second, in 2013, the debate on data sharing, both in the health service and broader society, started moving from a distant periphery to centre-stage. A key trigger was the media coverage of *care.data* – an initiative, subsequently suspended, of the UK Government to have pseudonymised patient data extracted from all GP records for the purposes of enabling service improvement and research, using an opt-out consent model. This led to a number of high-profile reviews on the topic of data sharing, a heightened public and professional awareness of the issues, and increased levels of caution and concern. Such developments further shaped the context in which the EoLC Data Sharing Project was implemented and affected its progress. This broadened the scope of the research-evaluation study, since, in line with principles of a realist evaluation, it was committed to a careful exploration of context.
- We were able to secure additional funding, which allowed us to extend the study duration and enhance its depth and rigour, e.g. we conducted a comparison with two other sites in England (see below) and performed a more extensive analysis of the qualitative data.
  1. **Methods of data collection**

The primary data collection methods of the broad study included:

- ***interviews*** (discussed in this paper);
- ***questionnaire*** on General Practitioners’ (GPs) and Practice Managers’ (PMs) familiarity, engagement with and perceptions of patient data sharing in England;^[[1]](#footnote-1)^
- ***ethnographic observations*,** mostly from the process of developing the data sharing system, but also from use of the data sharing tools in healthcare settings;
- ***document analysis*** (reports, meeting minutes and email communication of the service development project);
- ***case study comparison*** with two of the most successful EPaCCS in England, Coordinate My Care and the South West EPaCCS.^[[2]](#footnote-2)^
  1. **Relationship between the service development project and the research project**

Both authors were members of the team developing the actual data sharing system. SB was Clinical Lead for the project. MP was member of the core project team. This set-up significantly facilitated recruitment of certain types of interviewees (project developers, Steering Group members and key informants from the locality) as well as access to documentation providing a rich context for evaluating the service development project. While important study findings were fed back to the team and influenced some decisions, this was not an action research project or a formative evaluation. The two strands of work preserved their independence. For confidentiality reasons, SB (being the Clinical Lead for the project) has had no access to non-anonymised interviews and only to excerpts from anonymised interviews.

A range of ethical dilemmas arose due to the dual membership of the project development team and research team. None of them has compromised the integrity of the reported findings. We are preparing a paper on the benefits and downsides of evaluating projects with a diffuse boundary between a project team and an evaluation team.

1. **Further information on the development and contents of the interview schedule**
   1. **Development**

The interview schedule was developed iteratively by both authors. Members of the Lay User Group also advised on the formulation of questions for patients and carers to make them easier to understand and more sensitively expressed. The core schedule was adapted to the roles and context of interviewees. Gradually, it also began to reflect the researchers’ growing understanding of the issues arising.

- 1. **Contents**

**Core interview schedule for health professionals**

Version 3, 23 May 2013

This interview schedule will be adapted slightly depending on: 1) type of user (GP, district nurse, out of hours staff, ambulance services staff, hospital doctors, A&E doctors and specialist palliative care services staff); 2) frequency of use of “Share my Care”^[[3]](#footnote-3)^ to date; 3) rights for creating and updating records; and, for GP practices, 4) medical information system in the practice (SystmOne or other). Indications of adaptations are given before questions and at the end of the schedule. As the majority of interviewees will have used “Share my Care”, or at least will have been trained in its use, the core questions assume some use of the system.

1. You mentioned in our telephone conversation that you have already used the “Share my Care” system. Roughly how many times? [OK, we’ll come back to that in a while.]
2. How did your [organisation] become involved in the SmC rollout? What was your participation in the decision, if any? How were the System and the expectations of its use introduced to you?
3. How were you trained in using SmC? How did you find the training?
4. How do you normally use SmC? Can you tell me a story, situation, episode to describe this?

Sub-questions and prompts:

To what extent is it something routine? How do you go about checking for shares, reviewing records, acting on the information, updating records? How is the information you provide handled on the receiving end? Has your use of SmC changed over time? Has use on the receiving end changed over time?

1. How are you finding SmC? What is good/not so good about the template [“view”, if only recipients] and the processes of its use?

Sub-questions and prompts:

Are there things you would want to change in the template [view] or in the processes of its use? What are the sections that you are finding most useful/least useful? [if the interviewee has rights to create and update records] Are there sections that are particularly difficult to fill in? Any sections that you feel are missing?

1. [if the interviewee has rights to create records] How do you decide that a patient is suitable to be added on SmC? Can you tell me a story, situation, episode to describe this?
2. [if rights to create records] How do you go about discussing with patients their potential inclusion on SmC? Can you tell me a story, situation, episode to describe this?
3. [if rights to create records] How are you finding those conversations?

Sub-questions and prompts:

Has the introduction of SmC made a difference to the frequency with which you are having conversations about approaching death and end of life care? Or has it made a difference to how and when you approach them? Can you think of a story, episode, situation to illustrate these?

1. [if the interviewee does not have rights to create records] Are there cases when you think that a patient should be on “Share my Care”, should have been identified as an end of life care patient, and they are not? How frequently does this happen, if at all? What do you do in such cases?
2. Has there been any effect of having patients on “Share my Care”?

Sub-questions and prompts:

Has communication between settings and coordination of care improved? Have hospital admissions been avoided? Have some patients been able to die at their preferred place, which may not have happened if relevant information was not shared? Have patients and carers made any comments that can be attributed to the use of SmC? Or to teething problems with it? Do you feel you are now working more closely and/or know better health professionals from other settings? [prompt for specific stories again]

1. What are the things that are impeding or can impede your use of “Share my Care”? Can you give me an example when this has happened, if it has?
2. What are the things that will make you more likely to use “Share my Care”?
3. SmC demands a degree of record sharing which has not existed so far but which is setting in from a number of directions, such as through the use of the Clinical Records Viewer in hospitals, the simplification of sharing processes enabled through SystmOne, which are expected to come into force from August this year, Department of Health drives, etc. How do you see this growing move towards the sharing of patient data and clinicians’ records?

Sub-questions and prompts:

What are your main concerns? Are there organisations and professional groups you are more/less willing to share with? Are there types of patients about whom you are more/less willing to share information? How do you see patients responding to this increased level of sharing of information? Do you think such sharing should happen automatically or should a patient and/or their carer always be asked for consent? [If very much pro-sharing] Some colleagues of yours have expressed serious concerns about patient data sharing. Why do you think this is the case?

1. Media representations of the Liverpool Care Pathway have raised public anxiety about end of life care tools. Do you fear there might be misunderstandings, in the broader social arena, of the nature of End of Life Care coordination systems such as “Share my Care”? Or misuses of these? Do you feel that such public concerns may be having an effect on how you and your colleagues are using a tool like SmC?
2. Who do you think should have the rights to initiate a discussion with patients about them being added on the System and to create a record for them?
3. Who do you think shouldn’t?
4. How about rights to update the information?
5. For you, what is of primary importance in delivering high quality end of life care?
6. How does the availability and use of an end of life care coordination system relate to this?
7. Theoretically, now this is available for end of life care, it can be expanded to many other types of care, it can even become the norm of care. What is your view of this?
8. If you have to summarise your attitude to increasing levels of medical information sharing, what would you say?
9. Any other comments you would like to add?

If an interviewee has not used the System, start from question 10, having asked before the following questions:

- Have you heard of the SmC system before you received information about this study? What do you know about it?
- What is your view on having such systems [for sharing information on patients approaching the end of life]?

Questions will be revised for users of other medical information systems (different to SystmOne), depending on the arrangements for their use of Share my Care.

- 1. **Further details on the interview approach**

The interview approach combined principles of realist^4^ and episodic interviewing.^5^

The realist interview tests hypotheses based on a “rough programme theory” developed at the start of the evaluation. This testing of hypotheses is more explicit and collaborative than typical of most semi-structured interview approaches. The interviewer tends to share their evolving hypotheses, theories and findings and engage in a more active conversation as opposed to recede into the background.^[[4]](#footnote-4)^

Key features of the episodic interview approach include: 1) explicit focus on three types of situations, namely “episodes” (particular events or situations), “repisodes” (representations of repeated episodes, established practices) and historical situations (large scale events); 2) intentional seeking of data types such as definitions, abstract argumentation, explanations of concepts and their relations, and their discrepancies and contradictions with the personal experiences and practices recounted through episodes; 3) structured attempts to position the issue of interest relative to the interviewee’s biography and everyday life.^[[5]](#footnote-5)^

The interview style was generally one of giving much space to the interviewee to follow their inner conversation, reassuring them and validating their perspective. It did show some variation according to the role of the interviewee e.g. was more direct and at times challenging with health professionals and commissioners and more sensitive, supportive and empathetic with patients and carers.

1. **User involvement**

We benefited greatly from the feedback of our**User Group.** It had six members, five female and one male, all with personal experience of palliative and end of life care of their loved ones. We use the GRIPP2 short form items to provide a brief description of user involvement in this study.^[[6]](#footnote-6)^

*Aim:* Involving users aimed to:

1) solicit feedback on the sensitivity and clarity of the patient and carer recruitment materials (information sheets, reply slips and consent forms);

2) hear of personal experiences and opinions that could inform the patient and carer interview schedules;

3) seek advice on approaches to patient and carer recruitment;

4) get an overall feel for the broader patient and public knowledge of and attitudes to patient data sharing in end of life care. No plans were made for involving users in the analysis.

*Methods:* We recruited members by using existing collaborations (three members of the group had worked with the Palliative and End of Life Care Research Group before); through a practice manager (PM) advertising the opportunity in her GP practice and in other local practices (two members), and through an opportunistic conversation at an End of Life Care event (one member). We held four face-to-face meetings (roughly twice a year in early stages of the project) and had email exchanges in between them and subsequently.

*Outcomes:* Feedback by our User Group members transformed the patient recruitment materials, by making them far simpler, easier to understand and personal as opposed to legalistic and formulaic. We also added vignettes of experiences, some of which loosely based on stories our User Group members had shared. Some of the patients/carers whom we interviewed commented that it felt as if they were reading about themselves and this prompted them to get in touch with us.

In some respects, the new information materials were at variance with existing guidance. The Local Research Ethics Committee was, however, very positive about them and only requested that we re-position some of the information.

Our users also contributed to clarifying and simplifying questions in the interview schedule as well as provided feedback on one of the key papers of the study.

*Discussions and conclusions:* User involvement was crucial for designing effective recruitment materials, patient and carer interview schedules as well as for gaining an overall sense of patient and public knowledge of and attitudes to end of life care data sharing. It was also truly motivating, as the group was very supportive of our work.

*Reflections/ critical perspective:* There was no provision for Patient and Public Involvement (PPI) in the budget for this study. While we could enable it through broader research group funds, we needed to keep it within relatively narrow boundaries (e.g. we did not involve users in analysis, writing up and dissemination work).

Sadly, one of the PPI group members died during the study. While this is not unexpected in the context of palliative and end of life care research, it is still a difficult experience which can affect the cohesion of a group.

1. **Further information on the interview sample and data**

We conducted 40 interviews with 44 primary participants.

Two further individuals were primarily observers to their respective interviews (a junior doctor accompanying the health professional whom we were interviewing and a family member of one other health professional, whom we interviewed at home), but some of their comments were later included in the analysis or had an impact on the direction of the conversation.

The two leads of the project development team were interviewed twice in a short space of time (4-7 weeks), as the reflections they were willing to share were extensive.

While all interviews were planned as 1-person interviews, 7 interviews (3 with family members, 4 with health professionals) were with more than one participant. Apart from the above two cases where the second participant was primarily an observer, in all other cases the second, or (in one case) also the third interviewee, took an active part in the conversation. In one case, they were far more active in the conversation (apparently, they were designated as the spokesperson for the family) than the person we expected to be interviewing.

The main manuscript describes the four stages during which interviews were conducted. Two of the participants who did not fit the stage descriptions had unique roles for the sample (GP practice administrator and a hospital consultant in medicine for the elderly). Two others were steering group members and one was a palliative care consultant. One of the project leads was interviewed outside of any of the formal stages (Apr and May 2014).

Interviews were conducted in the service locality (the then Cambridgeshire and Peterborough CCG, one of England’s largest Clinical Commissioning Groups covering a population 0.89 million [69])^[[7]](#footnote-7)^, mostly in the workplace or house of interviewees.

Four participants (one patient, one nurse and two key informants with professional roles involving significant familiarity with the law) preferred not to be audio-recorded.

A professional transcriber transcribed verbatim 34 interviews amounting to > 285 000 words. The two final interviews, conducted after heavy saturation was reached, were transcribed verbatim by the first author only in sections we intended to use for reporting.

1. **Petrova M,** Barclay M, Barclay SS, Barclay S. Between “the best way to deliver patient care” and “chaos and low clinical value”: General Practitioners’ and Practice Managers’ views on data sharing. Int J Med Inform 2017;104:74-83. <https://doi.org/10.1016/j.ijmedinf.2017.05.009> [↑](#footnote-ref-1)
2. Petrova M, Riley J, Abel J and Barclay S. Crash course in EPaCCS (Electronic Palliative Care Coordination Systems): 8 Years of successes and failures in patient data sharing to learn from. *BMJ Support Palliat Care* 2018;8:447–455. http://dx.doi.org/10.1136/ bmjspcare-2015-001059. [Published Online First: 16 Sep 2016]. [↑](#footnote-ref-2)
3. “Share my Care” (SmC) was the original name of the service development project*.* [↑](#footnote-ref-3)
4. The RAMESES II Project. *The Realist Interview,* 2017. <http://www.ramesesproject.org/media/RAMESES_II_Realist_interviewing.pdf> (Last accessed Jun 2023). [↑](#footnote-ref-4)
5. Flick U. *The Episodic Interview: Small scale narratives as approach to relevant experiences.* LSE Methodology Institute. Discussion Papers – Qualitative Series, 1997. [↑](#footnote-ref-5)
6. Staniszewska S, Brett J, Simera I et al. GRIPP2 reporting checklists: tools to improve reporting of patient and public involvement in research. *BMJ* 2017;358:j3453, http://dx.doi.org/10.1136/bmj.j3453 [↑](#footnote-ref-6)
7. Office for National Statistics. Clinical commissioning group population estimates (National Statistics). Dataset. Mid-2019 Population Estimates for 2020. <https://www.ons.gov.uk/peoplepopulationandcommunity/populationandmigration/populationestimates/datasets/clinicalcommissioninggroupmidyearpopulationestimates> (accessed Oct 2022). [↑](#footnote-ref-7)
