# Supplementary material for: From “wading through treacle” to “making haste slowly”: A comprehensive yet parsimonious model of drivers and challenges to implementing patient data sharing projects based on an EPaCCS evaluation and four pre-existing literature reviews
Source: PLOS Digit Health. 2024 Apr 1;3(4):e0000470. doi: 10.1371/journal.pdig.0000470 (PMC10984410; doi:10.1371/journal.pdig.0000470)
Supplement: S2 File — (DOCX) [file pdig.0000470.s002.docx]

**S2 File: Further quotes**

This appendix offers further quotes which illustrate and/or provide evidence for claims made in the text. It also illustrates the richness of the data. It is to be considered in combination with the paper: quotes already in the main text are not repeated here, with some exceptions where a longer segment is provided.

Together, the paper and the appendix still do not represent the complete dataset we have used in our analysis. In some cases (such as the topic of Information Governance), we have included no relevant quotes as a paper is in preparation. In other cases, the quotes used to generate a finding were brief and matter-of-fact, while here we have included more detailed and evocative quotes. In still other cases, we have stopped adding quotes once differences in perspective diminish and new quotes are too similar to what has already been included. Further data can be obtained from the authors upon reasonable request.

We have not indicated the source of statements made by members of the core study team, individuals having leadership roles in the local health economy, and participants with unique roles. Attributing quotes to such roles would compromise participants’ anonymity, especially if several quotes are brought together. The source has also been omitted in cases of interpersonal conflict.

Quotes have been edited only minimally for excessive repetitions of crutch words (“I think”, “actually”, “you know”) or to remove threads of thought that take a different direction to the one we are illustrating in a particular case. Such edits are indicated by … other than for some of the crutch words, which, later in the process, were simply omitted.

1. **PURE CHALLENGES**
   1. **Radical innovation challenges**
      1. **Magnitude of repetition (“Banality of evil”)** – immense magnitude of repetition required both to set up the underpinning infrastructure (in different settings, teams, even computers) and to modify the behaviours and mindsets of individuals.

*Minor repetitive tasks or tasks with small variations experienced as particularly frustrating but potentially having safety implications*

“So we get eight different MDT [multidisciplinary team coordinator] projects instead of an MDT project with perhaps a little bit of local variation. It very much feels that you get eight different ones.”

*Interviewee 1:* “Some people call them a red star patient, some people call them vulnerable, there’s lots of different names for them and you're trying to work out what they are then … *[unfinished]*.”

*Interviewee 2:* “In [GP practice] a red star patient is a violent or aggressive patient.”

*Interviewee 1:* “'What’s [whispers] a green star patient …?'. It came up on the home page, green star, I thought, end of life, is this cancer, what, what is it?” *[Interviewee 1 – Out of hours professional 1, Nurse]*

*The work becomes mundane while most individuals working in innovation are seeking the excitement, creativity and buzz associated with it*

“I enjoy new things, so any new project is exciting. … But then, after a while, if the service is up and running, it becomes about performance management, and that is a bit repetitive, really.”

“For some services, it is a drag, it doesn’t run … and you’ll have to re-adjust, re-educate, resend out communications, begging your stakeholders … ‘you’re not using it, you asked for it and now it’s here, you’re not using it!'.”

*In some settings, the diffusion of innovation relies on a single individual*

“[A]lthough there was the commitment to this project, really, it’s only been my time and energy … my time instigated by me but supporting it within the organisation, yeah.”

### 1.1.2. Alternatives (“1001 nITes”) – the broad range of alternative tools and workflows available or under development

“I don’t do hospitals, I have got a yellow folder which is known to the ambulance men and paramedics, that’s all my notes and everything about me is all written in that folder … and I keep that in the bedroom.”

“[L]ast year, when we had an out-of-hours doctor come, it was useful for him to look at it because although he’d got his laptop, he could look up things, it was as easy to look in the book and see what was relevant.” *[both quotes Patient 1]*

“[Some practices] are happy to take on the tools that we’re providing for them and others are saying, ‘no, we’ve got our own system, we don’t wanna use that’.”

“I’ve forgotten [00:58?] what they call it exactly, but it’s like a summary sheet that we complete and we then send to [OOH service], and then that’s uploaded.” *[GP 4]*

“[W]e write, and I’ve seen other clinicians’ [letters] on patients who I’ve been looking after, I’ve got involved in near end of life, they’ve written, again, a free text letter effectively.” *[GP 4]*

“[P]atients who are fully engaged with their GPs understand completely what’s going on, whose family are all completely sensible will give us most of the information that’s on here. *[Palliative care professional 1]*

“[W]hilst information sharing through IT is a marvellous thing, talking to people remains a better option. And probably always will.” *[Out of hours professional 4, GP]*

“[T]hen we come to the summary care record. Okay? ... [T]he sharing model for the summary care record is different again, because the default was everybody share out unless they said ‘no’, so it’s a different sharing model again, in the same record we’re looking at.”

“We then come to the MIG, which I have no experience of as yet, but I’m very interested in, and I’m sure there will be a shared record related to that.”

“I talked to their IT man ... who is responsible for producing parts of the [new provider] solution, which is a single view of the record. ... I’m just trying to discourage him from launching a single view record on the 1st April that only has in it the drugs and the problem list and allergies, because that’s all he can deliver on the 1st of April.”

“A lot of data is automatically extracted from our systems, which is more anonymised [compared to care.data]. We have something called the GP Extraction Service, the GPES and then we have QMAS, which I don’t even know what that stands for, probably Quality Management.” *[GP 7]*

“[A]t the nursing home, they have their own DNR forms they want, which I’m kind of refusing to sign, I’m just doing those red-edged boxed ones.” *[GP 7]*

“[T]he district nursing team emailed me about a month ago a new form which [local community services] have requested they use, which is, I suppose, a kind of Liverpool Pathway replacement form, which seems like a huge amount of duplication and we basically said we’re not going to complete that form.” *[GP 7]*

- - 1. **Vicious circles (Catch 22 challenges)**

*Users are prone to give up quickly, while developers need continued engagement and constructive feedback*

“[O]bviously, the information has to be on it for, I don’t know, what do you think [turns to second interviewee], 90% of the patients?” *[Palliative care professional 1]*

“[I]f the special note or whatever is not picked up by 111 or the ambulance trust and then suddenly you have a GP saying, ‘Well, I had this patient, we did all this and then anyway, they end up [in hospital]. So, obviously, why are we filling this in if it’s not actually looked at?!’”

“[O]ur frustration was [that] we were completing all of this, and we had a few episodes where we knew that out-of-hours weren’t seeing our templates because if they had, they wouldn’t have sent in paramedics and started CPR.” *[Palliative care professional 2]*

“I say to them, ‘Please have a look at the things. You’ll probably find some of them useless. Tell us and we will improve the quality of it. But if you don’t actually start to use it, then we’re wasting all our time in the community entering the data’.”

*The mechanisms of negative learning are strongly hard-wired*

“We’re not looking at it that often and, as always with the bio-feedback, when you’re looking at it and you’re not getting anywhere, you think, ‘oh, I won’t bother again then’!” *[Palliative care professional 1]*

“[I]t’s a bit demoralising when you find the ambulance service won’t look at it! ... [Y]ou just think we’ve got better things to do, because if you have a rat and you want to motivate a rat to fill that in, you’d be giving the rat some praise while people are looking at this!” *[Out of hours staff 3, GP]*

*The emergent nature of innovation comes into frequent friction with traditional project management requirements*

“[E]very time we met, they would say, ‘Is our template there?’ ‘Have you told us what template you’re using?’ ‘No.’ ‘Have you had a project plan?’ ‘No.’ ‘Then what do you expect us to do?!’.”

*vs.*

“Every single project team meeting several things drop out and new ones come in and I don’t necessarily see the logic as to why it had to be that way, other than that key players didn’t necessarily bring the issues in fast enough, soon enough.”

“My contention is I could have spent … an extra day a week into simply getting a nice spreadsheet plan and changing the dates every single meeting. I could have done that. … I’m not convinced it would have changed the pace of the project.”

*The time and cost savings promised by data sharing first require investment of time and resources, in the context of overburdened services*

“[I]f you’re going to learn something new, you’ve got to have time to do it and, quite rightly, they’re focusing on seeing the patients who need to be seen and ‘this can wait, thank you very much’.”

“So we’ve got the whole system [struggling] when, without much effort, they could start to look at the information, which will help to join up community and hospital, and create some efficiencies for them, that they can actually meet their quality targets more readily ... [I]t was quite frustrating … how difficult it is to get them to move."

*The old can remain “good enough” until “broken”*

“I’m fairly stuck in what I was taught originally, for example the way that we write letters ... is really a very antiquated way of doing it. … However, it’s what I’ve used and what I always use, and what my staff use here ... [I]t’s not broken, so we carry on with it.”

“[I]f we were unhappy really with the way in which we use our systems, we would call for help. But as it is, we’re not unhappy, because we’re used to it and you just carry on doing the work.” *[both quotes GP 2]*

*Success can lead to disinvestment*

“[O]n the CCG level, the whole work stream around the End of Life care has run into the same problems in that there is no return on investment on any of these things. So it will all cost money rather than show any [left incomplete], because actually in terms of, maybe nationally, as I understand it, we’re not doing too badly on preferred place of death. So there’s not a lot, necessarily, we can do to move a lot of activity out of [hospital], for example.”

- - 1. **Uneven involvement** (“In the deep waters – toes in the water – high and dry”)

“[N]ot all the stakeholders necessarily have been kept up to speed, I’m thinking particularly about … the ambulance crew, which are absolutely integral to this and they’ve been allowed to slip off the radar and they’re the people that should be uppermost in that. I think representation from the out-of-hours service, whilst we had [IT Manager] … we could have benefitted from having someone specifically clinically out-of-hours orientated.”

*Interviewee:* “[T]here’s all this work done on these End of Life templates and there’s also the care of the elderly templates, which, in 111, we’ve got access to those, we don’t have them in [organisation] yet.”

*Interviewer:* “Don’t they?!”

*Interviewee:* “They do not!”

*Interviewer:* “Interesting.”

*Interviewee:* “I keep muttering about this, it’s something that I think should change, but at the moment they don’t have access to them.”

*Interviewer:* “I believe [name], who is on the team, believes that they do. But you …”

*Interviewee:* “Well [pause], I work there.”

“It’s partly our problem if the team that are going haven’t fed it back, then it’s yeah, that’s not so much your problem. If you’re giving them the information, then they need to pass it back to us”.

*Interviewer:* I was going to ask if there was a team discussion whether you would like to join?

*Interviewee:* [Laughs] Not that I remember! … We are using it because, as I said, we were really told we would have to use it and that was that, there wasn’t a discussion. Which is fine, but without really understanding what benefit it was going to give or how helpful it was going to be, so [left incomplete].

### 1.1.5. Overvaluing the uniqueness of one’s project or idea (“Lovely baby problem”)

"[I]t is interesting, very interesting to look at a cycle, whereas initially [template of another EPaCCS] was looked at, it was like, 'No, that’s far too big, we can’t use that', yet now they’re pretty much the same!”

“I know from meetings where [names] have gone to … demonstrate the Share my Care template, and, unfortunately, it was understood to them that it was a magical system … and [GP practice], who are very competent [System D] users and have been for nearly eight years, just laughed, laughed them off and just said ‘this is crap, we’re not using it’.”

“I think that will revolutionise the way [hospital] ED looks at some of these dying patients and some of these very frail patients."

“The perception was that it was a new system of doing things rather than making effective use of the system that we’ve got available to us by putting in some tools, some templates and views, and just making use of the systems we’ve already got. It was about embedding process probably rather than a system."

"[T]here seemed to be this myth that Share my Care was a system and its own thing and … that message has gone out to a lot of places. And it’s not a system, it’s a template and it’s a view, that’s all it is … And you need to have eDSM shares in place for that information to then be visible elsewhere. So it’s confusing to some people when they hear about Share my Care as being a system, because they believe it’s gonna do something magical."

*There has been some level of re-invention of the wheel, because of overestimating the uniqueness, innovativeness and capacities of the project*

"[W]e should have gone to see [names] very early on, knowing that they developed the template in [location], to say ‘how could we work together’, and we didn’t. And [name]’s been very gracious and patient in allowing us to redesign the wheel."

"Yes, there has been duplication of work and because the palliative, so the [location] one [template] was designed ages ago … [and] was demonstrated to the Share my Care team … and the team, I believe … [said] it was far too complicated, too much information in and … went off to create another template. So yes, there has been duplication of work, because really it is the same … it’s just a different layout, that’s all it is."

"[W]hen we initially became involved as a team, we recognised that the aims were quite similar in terms of our side of the work around the recording and sharing of the information, was very similar to, funnily enough, to the work that had already taken place at the [location] end. So it was just trying to share those lessons that we went through at [location]. And they said they needed this because of that and there was some quite detailed work, and we said, 'well, this actually looks like it’s the same, you haven’t got this or you’re doing this in this way, have you thought about doing it another way?'."

### 1.1.6. Securing a host/ sponsoring organisation (“Finding a home”)

"[W]here to base the project, that was a big challenge, and when we first went to the funding body they said ‘come back to us when you’ve found yourself a clinical base’ and we were pretty unenthusiastic about [Organisation 1], because they wanted an upfront payment and things, and [Organisation 2] seemed the obvious place and we’ve had to flex for all sorts of reasons, and we have got back where we didn’t think we were going to start [Organisation 1] and … they’re doing a great job for us."

### 1.1.7. Impact of delayed external innovation (“Innovation domino”)

"We’ve got [provider] who’s not able to manage that [daily extracts from GP practice systems], but as an alternative solution that would come at a cost, we can only install it in five specific PCs and we’d have to … manually run the reports daily or get [company] to manually run the reports on a daily basis."

"[I]f we do the extract on the 5th and they have an MDT … on the 1st … that’s pretty much a month out of date and … the whole principle of the Dashboard was to address the [clinical system] problem and so we’ve got to find a way of making that work."

### 1.1.8. Slipping timelines (“We are scheduled to finish 6 years ago”)

*Interviewer:* [T]heoretically, the project is supposed to come to an end in July [2014], we’re not ending in July?

*Interviewee:* No, [name] and I have agreed that we’ll continue funding him until December so at the moment the envisaged project end date is December 2014. *[Note Nov 2020: While certain aspects of the project are “business as usual”, in other aspects it is still seeking to achieve outcomes and to resolve issues which, in early 2014, were expected to be “done and dusted” by the end of that year.]*

"[I]t’s all very well saying, ‘we need to see everybody in … [LCG name] by the end of December, early January’, but if I email them and they don’t get back to me and then they can’t meet until such and such, it pushes everything back, so I’m very reliant on other people’s responding quickly [sigh]."

"[Y]ou have to take it quite slowly to start with … and then all of a sudden, which is where we are at now … everything’s taking off, and I can’t be everywhere at once … [I]t is always a bit of a slow burn to start with and then you’ve just got to be prepared for the ‘aaggh, I need to be everywhere by Christmas!’."

"[T]here’s an element of having to go back and redo some of the stuff that we could have been doing over the summer when we were sitting, twiddling our thumbs a little bit."

"The other thing is, it has required giving a lot of time to it and being prepared to give it the time that it needs."

### 1.2. Challenges of working in the health service (“Living on the Edge”)

### 1.2.1. Resource limitations

*Financial strain*

“None of our hospitals are truly viable, our [Trust 1] is not viable, but it might be rescued by taking on [Trust 2] staff, expanding. [Hospital 2], who knows what the future there is? [Hospital 6] is completely broke, it loses 25-30 million a year and it has a PFI [private finance initiative] that it’s short of another 25 million every year.”

"[T]hey’re absolutely cash-strapped, they failed to make Foundation Trusts and the management team are disappointed at that. They are a very, very challenged organisation and asking more from them in a field that the CCG sees as top priority and still piles pressure on them to do other things, we are dealing with what is regarded as a failing organisation as our main platform of making this work."

“And that is very depressing, and I think any public sector worker will say the same really that, yes, we are here to be looking after the public purse and we are quite responsible, I don’t, I don’t, I never not think about this money we’re spending, however people do get ill all the time and then the older the population and so on and so forth. So it’s really difficult to run what we see as an efficient, but basic, service for the type of money there’s available in the NHS today.”

"[O]bviously the CCG has a huge financial issue, particularly our CCG, so we’re redesigning services and we’re making a start on that process. ... [W]e always try and do thing properly, our team, so we have a series of stakeholder events planned for this month ... [W]e’re asking for patient feedback and for colleague feedback about what things work, what doesn’t work in a mental health service, but also being realistic. There ain’t any money, so we have to be very [careful] what we do spend, we want to get our best value for money out of that."

*Staff shortages*

"[W]e have more clinical advisors than Pathways say you have to have ... but you cannot guarantee to always have a clinical advisor available ... [S]o we can’t always warm transfer and we are, I think, warm transferring about 80%. So about 80% of the cases can go straight to a clinical advisor. On those other [20%], it would drop into a queue.”

“Now we’ve had a case recently, there’s been a lot of discussion, what we do if the clinical advisor isn’t there ... [W]hat they’re probably going to do is to still dispatch the ambulance, and then hopefully the ambulance will then pick it up and not convey it necessarily."

"[B]ecause the health advisor had seen there was the SPN for End of Life, they said, 'Right, I’m just going to put you through to one of my colleagues'. There was no colleague available. There should have been a floor walker, there should have been somebody who could have come round and just said, 'that can wait' or whatever, and I don’t quite know why at that point there wasn’t. And she hung on for one minute and 55 seconds or so, nearly two minutes. In the meantime she spoke to one of the coaches. So the coaches aren’t clinical, they are experienced call handlers, and also the shift manager, and they decided the right thing to do was to dispatch an ambulance."

"[W]hat we call the Community Access Team, which is really care, social care provision, there isn’t enough of that. Now making a diagnosis is quite easy that someone is dying, putting a syringe driver up is quite easy. What’s really hard is trying to clean them when they can’t get out of bed, trying to get them on the toilet when they can’t get out of bed without hoisting ... [W]e couldn’t get CAT support before someone died, this was last month. We need ... just someone to lift someone out of bed, a carer to go in urgently and getting those OT physio assessments, they need more capacity." *[GP 7]*

[in discussing priority changes to local End of Life Care] "[S]econdly, [it will be] to hugely increase the amount of hospice-at-home type of support, but not necessarily just so close to the end of life, just more hands-on caring. So it’s not Macmillan nurses going in and giving symptom control advice, but actually people who can be there and be overnight and care. And it doesn’t need to be particularly senior people, but people who know when they need to call for help, but can be with people and stay there. And so what would you call that? The hands-on day-to-day generalist end of life care, that would be my vote." *[Palliative care professional 3]*

*Disproportionate impact of staff shortages on remote areas*

"[W]e had a couple of people that died last year, one was a man with disseminated malignant melanoma ... [I]t was a Saturday afternoon, I had been visiting him on and off over the weekend, [nurse practitioner] had been visiting him on and off over the weekend, district nurses came in and they gave him an injection and his [pause] I get really upset talking about this [pause].

He was, and the district nurse said that there’d be another district nurse that would come at like 2 o’clock in the morning and give him another injection ... and then the woman was left on her own. I’d been there about 10 o’clock at night, she then rang, she then rang [nurse practitioner] at her home at 1 o’clock in the morning saying the district nurses had rung to say that they were too busy to come and see her husband and give him this injection.

So this was a woman with a dying husband, who basically had been told that she wasn’t actually important enough and her husband wasn’t important enough, and, urrrm … so, as I say, she rang up [nurse practitioner], [nurse practitioner] rang me up, I came out at 2 o’clock in the morning and gave him this injection. Wasn’t that, he in fact died about 4 hours later on, which was fine, and all the stuff, but it was the way that my patient’s wife had been told basically that they weren’t really important enough."

"[I]t was bizarre because 2 weeks later on, it was this time of year because I was in [city, distance] and ... another lady and this man had, he had liver cancer and I’d been quite involved in him again, and his wife rang me on Sunday morning and I was in [city] at the time, to say that she needed help with her husband. And I suggested that she ring the district nurses. She rang the district nurses who somehow or other said that she didn’t need a visit and they would come and see her next morning, come and see him the next morning."

"[H]e’d actually fallen out of bed and she couldn’t get him back to bed, so when I came back from [city] that day on the Sunday afternoon, I went round to see him and he was dead and he was on the floor, because she couldn’t lift him and because the district nurses had said again that he wasn’t important enough."

"[A]nd the reason for this was not because they are awful people who hate my patients, it’s because the community nursing service are based elsewhere and it takes half an hour to drive out here and see somebody, and then half an hour to drive back, whereas if they’re in [cities], it takes them 10 minutes to drive somewhere."

"[S]o we get left, we get put to the end of the line."

"[T]he point of the story, these appalling stories, is not the fact that we are wonderful people, it’s the fact that because [of] where we are, we can’t trust anyone to do stuff. So as much as we do information sharing and as much as we use IT processes, we’re in the middle of bloody nowhere and no one likes coming out to [village of practice]." *[GP 2]*

*Staff turnover in settings particularly relevant to end of life care (e.g. care homes)*

"[Care homes] don’t pay particularly well, and so their staff retentions are low and if you can get new staff, they leave quite often. They recruited from India two years ago, and they recruited a lot of females, and they’ve all got pregnant, so now they seem to have changed their recruitment idea to taking more male Indian nurses. But they come with a visa and they get stuck there for a certain time, and there have been some quite difficult issues. They’ve not been very kind to some of their staff ... [O]ne lady miscarried twins at 20 weeks, because they’d made her work many extra hours, because they couldn’t get staff in to one of the units ... [S]he was devastated and eventually she left six months later.

So the staff just get up to scratch and we train them and we get them using, doing the DNACPR and getting engaged with it, and we get them familiar with the Just in Case bag charts and then we lose them ... [W]e need to continue to educate the carers in the care homes because they turn over quickly." *[GP 3]*

"[I]f one leaves or a new person comes along, then they’ve got to learn all again, but I hope there’s enough groundswell of people who feel that it is useful and does prompt conversations." *[Palliative care professional 5]*

*Stretching of roles*

"[W]e’re stretched incredibly thin as well. ... I had a team of three for supporting 27 practices a couple of years ago. I lost, a member of staff left, because then we were really, really pushed as well, just for 27 practices and one PCT. That vacancy got whipped away from me. Then when they restructured into the CCG, I was slotted into the post which, I still got a job, I’m not complaining on that basis, but I was slotted into the post at the same band, no extra money. And it was, like, you’re given another 70-odd practices and eight more organisations and albeit you can have an extra member of staff, but I had that extra member of staff before!"

"I’m the CCG [condition] lead, and that is an enormous role, so whereas I’m contracted to just cover the adult service, so that’s adults of working age, unfortunately, we didn’t manage to appoint to the older people’s post ... so I’ve ended up being interim for that as well. You probably know about the procurement locally, so that’s involved huge amounts of work, so I drafted the outcomes framework for the mental health indicators, was involved in interviewing the bidders, marking the bids, and, as I say, now, technically, that’s not my role, but there isn’t anybody else."

"The clinical lead role is supposed to be ten hours a week. In reality, I’ve been recording it as 18, but I’ve probably been under recording."

*Difficulties recruiting, including because of financial counter-incentives*

"I also worked on a project around diabetes which was binned in the end, because we couldn’t see the return on the investment, partly because of the lack of supply of diabetes nurses. We can’t employ them, there’s no money around, so we can’t actually make community services because we can’t fill the [posts?] with clinical staff ... [W]e have all these great ideas, but if you can’t find the staff, then you can’t do anything about it."

"One of the reasons why they can’t get enough out-of-hours doctors to fill the slots in [location] is that if you do out-of-hours, it virtually doubles your medical insurance. So my colleague would pay about £6,700 a year for her medical insurance, [but] because she was doing 4, 4-hour, 8-hour sessions at [OOH] a week, they put her defence union subs up to £11,500." *[Out of hours professional 3, GP]*

*Saturation with certain aspects of one’s role*

"So we decided that we’d done as much education as we could do, because we’ve repeated some of it and we’d used other resources and quizzes and questionnaires, and we’d engaged nicely with [names]. But we’d reached the point where we were all a bit fed up with it, and we couldn’t, with time, and other people’s time, we decided that practice engagement was for the next year."

"I think the first one was so successful because I was so passionate about it and keen about it, and many of the relatives were on our Patient Participation Group, so they’d actually come to [educational events] as patients ... we’d had patient engagement as well ... [S]ome of those people have died ... I just feel that the enthusiasm’s gone a little bit, we’re all a bit exhausted, haven’t got time, can’t think of anything new to sort of inspire people with." *[GP 3]*

*Insufficient equipment*

"[S]yringe drivers, we purchased some for the surgery and staff, but accessing a central [storage/ resource], the CCG have a number of syringe drivers in a locker somewhere, getting those can sometimes be a problem, but we’re alright now because we’ve got enough." *[GP 7]*

*Inappropriate use of services resulting from the health system being free at the point of care*

"If you provide a free service in a financially focused society, you’re immediately taking away one of the constraints that can adjust patient demand. And because there is no evidence of any value of the interaction, people think that healthcare costs nothing. So, again, I’ll see people in A&E, one particular young lady who accidentally stabbed herself on the leg with her pencil and came to A&E, and you’re thinking, ‘have you put a plaster on it, have you cleaned it?!’. They have done absolutely nothing, because they don’t see why they should make the effort to have some sticking plasters at home, it’d be easier to go to A&E." *[Out of hours professional 3, GP]*

"So the NHS is being used very much as a sort of [pause] barn to just keep people generally happy, but it’s so expensive and it’s too easy to use it inappropriately, and if I have the foresight, because we had more joined-up care, then of course I would be able to start the consultation in a much better place." *[Out of hours professional 3, GP]*

*Changes in society that impact on the workload in the health service*

"I’d been thinking it again the other day, what’s going on in [location] that this summer there was no lull in activity and the winter pressures never relented? So by the autumn of this year, and it was just a month or two ago, we were already in a bed crisis with routine, this is before [clinical IT system] came in, with routine admissions being cancelled."

"And I was just noticing that the streets are so much more full, everything is so much more full, the classes in school are filling up with new children who keep appearing, and there is an overwhelming amount of new building in [location] at the moment. [Location] is unrecognisable over the last three or four years."

"[I]t’s been a massive acceleration in building and people moving into the city, and my theory, for what it’s worth, is that that’s why it feels like everything is precarious, even before [clinical IT system] came in, because the existing services have become, and not just health services, but everything’s becoming overwhelmed by the influx of people."

"[T]here’s a huge influx and it’s a positive thing, and it’s one of the most booming economies in the country, and there’s so many good things happening, but inevitably infrastructure lags a bit behind. And then they build the roads and then they find the parking, and then they build the schools, and then the hospital gets bigger and so on. So I think there’s probably some more fundamental things going on, which are contributing to the pressures, and then with [clinical IT system] on top of that, that makes it particularly challenging at the moment.

But, you see, you can turn that round and say the positive thing is that these IT systems are coming in, [names], at the time where everything would have become suddenly unmanageable if there wasn’t the order that comes from a virtual system." *[Palliative care professional 3]*

- - 1. **Fragmentation**

“But one of the main frustrations has always been the structure of the organisation, when I say ‘organisation’, a not very organised organisation!”

*Significant levels of autonomy of organisations, and to some extent professions, and associated lack of clear mechanisms for driving multi-setting projects*

"[H]aving that degree of stakeholder thing was important because people don’t like the idea of something being forced upon them. I guess it’s a problem with having lots of contractors, lots of different services, lots of professionals, nobody likes being told from on high what they do and they don’t have to do. So having those groups are important to get people to feed into things."

"[T]he very structure of the NHS these days is that doing something that is as system-wide as I’ve ever come across, finding the leadership is very difficult."

"There’s just no effective mechanism to drive it through."

"It’s nothing to do with this project, but I do not understand why a) we need a purchaser–provider split and b) why do we need acute and community services to be under separate management … [T]he performance of community services to take hospital discharges is absolutely vital to success but, on the other hand, the moment they take those discharges, they’re clocking up costs and going bust."

"I think this is a reflection of how unmanageable the NHS has become. Very disparate."

"[I]ndependent practitioners in GP land are very, very difficult to marshal."

"I am very concerned. A lot of GPs are very concerned about where the NHS is heading. Obviously, we’re very fiercely independent, that's why we are like we are." *[GP 1]*

"We know that GPs in particular don’t have to do this if they don’t want to, because they’re independent practices, they don’t have to."

"[T]he people that you’re trying to present it to are very strong-willed and single-minded sometimes, they don’t automatically buy into everything that everybody says, quite rightly … They have enquiring minds and they want it to be proven that it works … So I understand that, but it’s hard work sometimes."

"[Y]ou don’t have to use all of it, and it’s always dangerous to tell GPs, because they all like to do different things, that you have to do anything. That’s like, 'Well, actually, use it, see how you get on, start off just using two or three pages of it, and then you might want to use more of it as you get familiar with it'.” *[GP 6]*

*[has changed since interviews conducted – LCGs, i.e. Local Commissioning Groups, no longer exist]* "[W]e are a big organisation, which was kind of sold on the basis of economies of scale and all of that, yet all we’ve done is, we’ve split it up into nine organisations, because we’ve got eight LCGs plus a CCG. And to those of us trying to support all of those customers, plus a hundred and seven practices, it’s incredibly fragmented and there doesn’t seem to be much cohesion at the CCG level, sort of steering the LCGs.”

*Multiple projects and initiatives competing with one another*

"[I]’s not an isolated problem. So the problem with end of life is part of many problems for 111, one of many of the ambulance trust problems, so it might drown a little bit in the priorities. Or trying to make that a priority in every discussion might be difficult, because there is multiple clinical problems."

“But could we get them [“the hospital people”] to attend the meetings reliably? No, because it’s not a priority to them.”

"[T]he approval is difficult because there are so many parallel projects going on, and it’s more about prioritisation of the work that we can genuinely commit to, and to commit to do it properly and well enough. So although there was the commitment to this project, really it’s only been my time and energy … my time instigated by me but supporting it within the organisation, yeah."

"[O]bviously, we have to compete on any project, on any initiative, we have to compete with many, many others."

"It just feels like there’s project staff come in and out all over the place and the rest of us are working our [pause] backsides off”

*Some of the preceding or concurrent domain-related projects (in this case EoLC) may have been exceptionally comprehensive, which makes taking up a new project in the same domain particularly difficult, even if it has more generalisable benefits (in this case broader data sharing).*

“For that project [name] we had a target of … about 2,500 Group B staff [staff who have frequent contact with End of Life Care, e.g. acute hospital staff, district nurses, ambulance crews] in the two years of the project, we then got a third year’s funding … [to] deliver the training package to at least 20 new care homes … and we had to train staff using a bespoke Train the Trainer programme for 18 care homes.

[A different project is] for clinical staff but also … for non-clinical staff, admin people, reception … based upon a learning needs assessment of their confidence and knowledge around End of Life Care … We attend a palliative register meeting or GSF meeting, we make observations as to how their palliative register meetings run, the sorts of issues that they’re encountering, how many patients they have on their palliative register list … how many people attend, what’s the skill mix of the people attending, do they get the district nurses in, do they get the specialist nurses in, how frequently they run, how long do they take, all those types of things.

And then we ask them to complete, as a practice, 10 confidence questionnaires, looking at how confident they feel around issues such as running palliative care meetings, having difficult conversations with patients, managing symptoms, setting up syringe drivers, knowledge of Liverpool Care Pathway, those types of things.

And then once we’ve got those 10 questionnaires back, we look at them in conjunction with the observations from their palliative register meeting and we create a bespoke training proposal for that practice, which we send to them.

[A]nd it’s for the practice to decide what they want to do, how long they want to spend on it, and I will facilitate that learning in some way, shape or form, free of charge, at their request.

[T]here’s 108 [practices] and I’ve had contact with 67. … [T]hat’s not always necessarily been that I’ve been to 67 Gold Standard Framework meetings or palliative register meetings but … somebody within that practice has had some educational component from me.”

*There are several initiatives which have different sources of funding and support, cover somewhat different areas, but have largely the same goals and stakeholders and require the same types of input.*

"[T]he challenge around the ‘is [location] in Share My Care or out of Share My Care?’ was a bit frustrating because what we’re saying is 'does it need to be in or out of Share My Care? Are we not trying to do the same thing? Is it not End of Life Care summary in a different guise?'. And it isn’t different, but there was this perception that it was [location project] versus Share My Care. And that wasn’t just from the Share My Care team, that was from some of the [location] leaders. And we were trying to open that out and say, 'we’re not talking about will you play or won’t you play, we’re already playing the same game with a different badge'."

*Interviewer:* And have you seen the End of Life Care Cambridgeshire template?

*Interviewee:* Right, well, you see, I’m aware of several, there is the End of Life care template which I was introduced to by [name] ... I don’t know whether that’s the same one? ... [T]here’s then, there’s the Share My Care thing that came out of [pilot area].

*Interviewer:* Yeah, interesting, because Share My Care is the old name for this project and [area] was the pilot LCG.

*... Interviewee:* So the one that we’re talking about is the one that used to be called Share My Care? ... So there was Share My Care and then, but the one that we were told about, which was the [location] one with [name], that’s separate? Or are you all friends together? *[GP 2]*

*Insufficient national/ central leadership and control*

"[T]here’s no pressure from Strategic Health Authorities or NHS England or the Department of Health … to say ‘you should not be having 7 ambulances sitting in, queuing for a hospital to unload’."

"Arguably, if the pot of money is NHS expenditure, then the CCG should have some leverage in how it’s used, but in practice the CCG has little influence on how [hospital] choose to use their money. And if [hospital] do have a pot that’s for End of Life Care, I would suggest that the CCG have very little influence over that."

"[T]here’s been a lot of confusion pertaining [?? 37:30] to the different sorts of consents and data sharing, so the *care.data*, which came at a similar time, did confuse. So, again, more national clarity on data sharing and what it means and that there are many sorts of data sharing projects." *[GP 4]*

"[W]e were expected to discuss it with everyone, when we just didn’t have the capacity to do that, [we] put up various displays on the wall in the waiting room, but they are only seen by people who are sitting opposite that display in the waiting room. It should have been a much more national approach, it shouldn’t have been delegated to local services to do and there should’ve been a much more streamlined way of patients getting out of it if they don’t want to or withhold consent to that sharing." *[GP 7]*

"I’ve got a huge issue at the moment, because one of the Government targets ... is dementia diagnosis, which is actually a bit ridiculous, I don’t support it at all, but it’s a CCG [target], it’s difficult for me, because my role is to meet the targets. And the difficulty is, obviously, that sits in primary care, and I’ve got no levers in primary care to make GPs do it."

*The targets of different organisations are not coordinated around integrated patient care and collaboration with other settings. They become self-serving concerns for organisations and sources of huge inefficiencies.*

"The moment you establish an NHS where an Acute Trust is paid to hit these targets, an ambulance service is paid to hit these targets, a Community Trust for these targets and they’re all different, each organisation focuses on what keeps the Board in a job."

"The Ambulance Service are dominated by hitting their 18-minute response times and … you can quite cheerfully roll up at [hospitals] and look at 6 ambulances all queuing because in the A&E department hitting the 4-hour waiting time target from arrival to admission … is far more important in the eyes of the Department of Health than the ambulance target. And so the A&E department doesn’t want to crowd itself up with ambulances coming and unloading patients … and so ambulances are made to wait."

"[I]f you’ve got 8 acute hospitals across [area], then you can quite easily have 30 of your ambulances with patients inside them sitting at those hospitals. That only leaves you about 10 available to respond and you’re supposed to hit response times. And it’s just crackers."

*The lack of familiarity with other parts of the system, resulting from this level of fragmentation, often leads to unjustified blaming*

*[Interviewee describes in some detail the case of a hospital admission which led the patient's GP to express strong frustration with how the case was handled.]*

"Daughter rings, says, 'My mother’s breathing, she’s got a breathing problem, but my mother’s breathing has got decidedly worse in the last hour, and [?? 19.42]'. I’ve listened to the call, the call handler actually handled it very well. She didn’t just accept that, because in Module 0, about the third question in [it] is, 'Is the person fighting for breath?'. Now this really means gasping your last [breath] ... not just you’re breathless, not just you’re having a bit of an asthma attack, which will then, if breathing is the main problem, get further probes into the Module 1."

"So the call handler probed further, 'Can I hear your mother speak? Can I listen to the breathing? Could you get her to just say a sentence?', because if you can give out your address, then the answer is, 'they’re not fighting for breath' ... [S]he tried to get her to read the first line of her address, she wouldn’t do it, she went [makes croaking and gasping noise] and so, at that point, the call handler said 'yes' to that question. What it then does is, it says, 'Right, we’re going to dispatch an R2 ambulance'. An R1 ambulance is if someone is arrested and unconscious, and R2 is the next one down."

"But instead of dispatching the ambulance to her, because the health advisor had seen there was the SPN for End of Life, they said, 'I’m just going to put you through to one of my colleagues'."

*Uncertain status of the involvement of an organisation and its employees in many cross-settings projects (may not be resourced from new funds, but still expected on the grounds of “we are the NHS”)*

"But when someone is running a project completely based on goodwill … I have taken the view … it is not a good thing for me to be leaning on people and demanding of them to do it faster or quicker or … better, if what they’d done was not good enough … [N]ot when it’s not their main job."

"There certainly have been times where … I had thought, if this had a person who wanted or was willing to push it, push, push, push, hard, hard, hard, bully, would that help? But my take on the situation we’re in is that … we’ve got a whole load of NHS employees [on the project team and stakeholder group] and we rely on their contribution as a point of goodwill, all of them can, in essence, refuse to cooperate."

*Existence of multiple support structures, also undergoing (frequent) change, which makes it hard to identify the right expertise*

"What I wish we had done is much more actively sought out [name]’s team's expertise much earlier, because she’s been absolutely superb. And it was months before we realised that she might be available to support us, although we’d heard her vaguely talked about as being somewhere up in [city]. So that is something that I wish we’d thought, 'uuurrh, we need to go and talk to her'."

"[A]t the end of March we were invited to a workshop being run by [team] … who were the specialists in the CCG for designing templates and views. And I did think it extraordinary that it had taken over a year to be put in touch with such people."

"[W]hat would have been much better is if someone had alerted us to the fact that there’s a team up in [city] who design templates. Don’t even think about using a practice manager to develop this … [N]o mention that 'you know we have a team up in [city] who are experts in doing this?'. And I don’t understand that."

"[W]here there’s been some blurred lines or some difficulties initially is that [colleague]’s team [pause], not that they should have, but they don’t have the same understanding of the clinical records side and the information side as my team do. So some work is done over there that we might come in if we find out about later and say, 'actually, it might be better to do it like that' or 'that doesn’t fit with this' or 'we’re already looking at that'."

"[I]t ought to be clearer than it is, but in practice there seems to be a bit of a mix. And a lot of that is history, because my team existed for a long time in [location], but is still relatively new in the CCG, and a lot of the folk like [names] historically didn’t know there was my team to call on to ask those sort of questions.”

“So it probably did all sit with people like [colleague's team] to do, because there wasn’t the other side, whereas ... to me it feels like it should be [colleague] saying, 'there’s this project going on, I’ve been involved, they want to use this system, they’re saying they need to record this, I need to get them to come and speak to you, about how they do that'. And so working together, but with each using their own expertise in certain area.”

And ... there’s a bit of the same issue around the dashboard side of it, because I know that my manager [name and role] would potentially feel that a dashboard around monitoring information is actually business intelligence, not IT."

"I don’t think, as a CCG, things are that clear about remits within the teams. And that doesn’t help with people trying to run projects, because there’s not a clear [left incomplete], and something that we’re all trying to work on is communication about ‘this team does this, and that team does that’, and it is a bit unclear."

*Challenges in boundary areas or settings that cover multiple localities*

"[T]he trouble is, the Ambulance Service is huge, and we’re only a very small part of it. That [incentives for them] has been brought up before, but they, they’re not [left incomplete]. It could be [new provider] will negotiate something with them, so we’ll see."

"[A] lot of patients are tertiary referral patients so they’re not even living in [area] ... So because of all the tertiary referrals centres, they are coming from all over East Anglia." *[Palliative care professional 1]*

"So our CCG commissioned a service, but for [area] it’s commissioned from an organisation called [name], who started life as the out-of-hours provider in [location, somewhat distant], it’s a social enterprise company. The one bit that’s about to change ... there are three practices in [LCGs], their service will be provided by [location] ... because they want their patients to be looked after by [location] out-of-hours which is going back to [hospital], which is closer to their system for them."

- - 1. **Constant transformation**

*Projects come and go and this confuses health professionals*

"[T]o try and change something, people put in a new structure, so then the GPs can’t remember, 'do we do that?', because there was this vulnerable patients scheme and there was a yellow folder in patients’ homes, which when I was doing the out-of-hours at [location] and it isn’t [30:54] mobile, but really, really, really helpful and that’s been stopped."

"[W]ith the End of Life Care, I feel that it would have been much better if we just extended the vulnerable patients scheme, where in the out-of-hours base there’s some big folders where ... we can go and look and get all the background information and then take the call and discuss it with them with that benefit."

"One of the troubles the NHS has is that it never lets systems bed down, they always change before they have and so, and because everything is changing, the message never gets down firmly to the lower levels that are providing the patient service." *[Out of hours professional 3, GP]*

*There is a pervasive project fatigue*

"There is template fatigue and there is Dashboard fatigue out there, but that is nothing particular to the project, that’s just the way people are at the moment, and there’s a considerable amount of initiative fatigue because there seems to be a new initiative every day in the emails that come out."

"Some practices have gone ‘oh, bloody Share my Care, I don’t want anything to do with it’. And that’s not anything against Share my Care, it’s just another project that they can’t be bothered to deal with because they’re up here with other things … it is very difficult for practices because they’ve got so many targets to meet."

“[S]ome GPs will be very aware that they’re so busy trying to meet targets that they’re no longer caring for their patients, so they get very frustrated when new projects and new tools and things are put in their place because they think ‘oh, another thing I’ve got to do that doesn’t allow me to sit and look at my patients and make sure that they’re OK’.”

"GPs generally are quite sick and tired of being asked to do more and more initiatives on the grounds that they’ll be really good for patients, they’ll be really a step forward when A) they’re a load more work and B) there’s no more money with them at all. And during times of plenty when funding was reasonably good and the workloads were manageable, then I think that that was fine, but, at the minute, I don’t think general practice is particularly receptive to doing more stuff without any financial resource to do it, just because somebody says, ‘well it’s a good thing and will help people’. I think it’s very much ‘what’s in our contract, are we going to be paid for it’, so that’s a kind of sad state of affairs really."

### Environment deprioritising extras

*Relentless, overwhelming pressures, affecting the capacity and morale of staff and organisations*

"I do feel that the NHS is going to collapse either this winter *[Note: interview conducted Sep 2015]* or next winter depending on what the weather’s like. The system is grossly overloaded and because they are working harder and harder, they’re making more and more mistakes, which is becoming more and more costly and causes more admissions to make it right." *[Out of hours professional 3, GP]*

"[P]rimary care’s on its knees at the moment ... so really just anything is extra is too much." *[GP 6]*

[I]t’s very hard to be proactive when one’s overwhelmed. *[Palliative care professional 3]*

"[O]ur key workforce are the district nurses and they are so under clinical pressure that implementing change with them has to be very carefully thought about in order not to just overload them, because it is their clinical priorities versus generating the change in practice that’s needed, so we’ve had to pace that very sensitively and carefully."

"But sometimes it’s when you’re very busy and you’ve got a lot of patients, I don’t automatically create a template on Day 1, but I always create it because I’ve been told we have to and it’s going to be audited and we were audited and we were *[?? 41:41]* 100%. ... I just basically feel sometimes it’s just another thing we’ve got to do on top of everything else." *[Palliative care professional 6]*

"[T]here was a time where I was doing a ward round on Fridays to try and manage the workload, but it could vary between four and fourteen visits ... [T]o actually see that number of patients and then come back and write up all the notes, it can really push the two hours and you’ve also got other letters to write and other paperwork to do and outcomes from those visits, like arranging swabs from *[?? 07:15]* screening or MSU forms, bits and pieces, so it was really proving very difficult to [left incomplete]. So, and it’s partly the way they [care home managers] were managing it and not supporting the staff well enough and the staff just felt they had *[?? 07:32]* to call the doctor for everything."

"Probably the problem with some of these care home patients is you just haven’t got sufficient time, they are the ones that actually should have them [templates], but it is enough funding and getting time to actually have enough time to just check you’ve done it, because you know them, you know the patients inside and out." *[GP 3]*

"[O]ne of the factors that has slowed it down has been clinician engagement and clinicians are clearly at the moment in every setting, both primary and secondary care, busy and preoccupied and if we’re basing this largely around general practice, which we are … they’re not looking for things to spend time on."

"[T]hat’s the feedback I hear from GPs, they have ten-minute appointments, and they can’t spend five minutes of it explaining EDSM and getting EDSM consents, because they actually need it for their consultation. So finding the capacity with both GPs and community staff to fully implement EDSM is challenging."

"[K]ey challenge is the staff time to really take the change of practice on board."

"[N]ow, of course, the issues that we’re struggling with are those of GP engagement at a time when GP practice morale is falling fast and funding is decreasing for GP practices."

"Maybe staff who are within an LCG feel differently, but those of us in teams that are supporting at the CCG level ... it’s, you do feel [pause] I’m going to say 'undervalued' for want of a better word, but you don’t feel as recognised. You’ve got various project managers based in LCG’s who go about sorting things out without consulting the right people, and then you feel like an inconvenience sometimes when you say, 'hang on, we could help with this', or then you get the 'why didn’t you help with that?'."

"It just feels like there’s project staff come in and out all over the place and the rest of us are working our [pause] backsides off and, and it isn’t acknowledged, I think. So that’s one of my frustrations and when I’ve been around such a long time, it’s probably more frustrating for me having been around not so many years ago where your input was more valued."

"I do fear for winter pressures … we have a lot of experience over recent winters, how stretched community services truly get over the thick of winter and … that’s a big blocker, that I can’t engage the staff because they don’t have the capacity left to engage."

"In some weeks, it can be twenty visits and then some weeks it can be ten, but I wouldn’t say it’s any less than ten, and then sometimes … it’s a bad week when you might have thirty or forty visits up there if there are chest infections and gastroenteritis outbreak." *[GP 3]*

*Focus on “core business”*

"We’ve tried to engage the Ambulance Service with the Stakeholder Group and they’ve stopped attending that. They just don’t have any money to spare the time. It’s not important enough to them."

"[T]his project is potentially helpful to them because the theory is they will take fewer patients to hospital … I’m not sure, but the Trust would probably lose money, it gets more money for each transfer to hospital than it does for just a call out … so there are predicaments there. But it won’t be at the top of their list, because at the top of their list is 'we get to a patient within 18 minutes of being called'."

"[I]t’s one of our best, better successes. Well, it’s a very good success story really. The problem is that we can’t expand this because the ambulance trust, at the moment, has been asked to focus on meeting their targets only so they’re culling every service. And you think this is the best we can do for admission avoidance, but we can’t expand it because they’re not allowed to focus on these things."

*Culture of responding, firefighting and urgency rather than long-term planning*

"[Y]ou find that perhaps something hasn’t been thought of in that initial stage or something that has an unintended consequence, and so you end up firefighting issues for a greater duration of the project after go live … [W]ereas you would devote six weeks to the project if you did it properly, you’re probably devoting ten weeks to the project to get it all in and … then of course … project 63 is drifting further away and somebody else isn’t happy about it and then we’re falling further and further behind."

“[S]ome things we have to do just because something’s happened and a service will lose their N3 connection by tomorrow if we don’t drop everything and we do something about it … [W]e have to be very good at responding to changing needs.”

*Short-termism*

"[O]ur financial commissioning cycle is a year only, so it runs from April to April, and obviously nothing, no service, nothing in the real world runs for only one year. So we can’t do long-term commissioning, we don’t see the results of what we put in place, we don’t allow a project to run enough or long enough to pick up and it becomes embedded into the health economy before we have to evaluate whether it’s value for money. And that means that we open and close, open and close quite a lot of projects, and that’s quite depressing."

[I]t’s really difficult to run what we see as an efficient, but basic, service for the type of money there’s available in the NHS today. And all sort of long-term strategies about your chronic diseases, it goes out of the window because we can’t show a return on investment in the same year. So diabetes prevention for example, we know diabetes costs billions every year, but we cannot put in long-term strategies, that takes 5-10 years, when you have to put in, spend a lot in a community and then we’ll see an effect when people grow up or when they get older. But we can’t plan with that and that is quite sad."

*Crude decision-making models overdetermined by a concern for saving money, NOW*

"You have to remember our way of calculating things is very, very crude. So it’s very much like 'this will do that', it’s not like 'this and then this and then out here and then coming back and then, oh, registered care, blah, blah, blah, it would result in that'. It’s very much, 'Will this stop an inappropriate admission? Yes or no? Tomorrow'. Much like in that [laughs]."

"Plus, a lot of time goes by filling in yet another template with another report attached to it or, and also a little bit putting your finger up in the air, 'oh, I think it’ll save this much money', but doing that without having had the time to scope out. So we, as an LCG for example, would have agreed to a budget and we agree to a number of savings we have to make and then, after that, we work out where to find those savings. You already know what those savings have to come in to, so everything you do after that is really quite constraining, because you will have to find the savings some way or another and it’s not that easy. So we sort of say something and then we run desperately after that goal and see whether we can do it or not.

And often we can’t, because it’s not influenced by our intentions, but whatever people do in the community ... [Y]ou might have a very hot summer, like this, and all our dermatology referrals go through the roofs, because everybody comes home with a skin burn or another *[?? 07:56]* and think they have cancer and then they go to the GPs and then they’ll be referred in ... [Y]ou can advise, you can educate, you can, but you can’t actually plan this."

*High levels of attrition and replacement of representatives from different organisations, with replacements often coming from lower hierarchical levels without an appropriate handover*

"I'm sure one of the weaknesses for people like [Project Clinical Lead] and the computer people who’ve been involved - I don’t think weakness, more frustration - is the turnover of people who attend. So people are constantly coming who haven’t been before and are perhaps not up to scratch, and it must be hugely difficult when somebody comes to it new and thinks they know everything and starts telling you things that you actually have visited before. Not a problem for me, but I’m sure that must have been difficult in terms of moving it on in a timely way." *[Palliative care professional 3]*

*[of another project]* "I feel that it’s rather fallen back and we had, as admin, we had [names and roles, higher level] and they’ve gone onto more, bigger issues and left us with sort of lower LCG admin people who haven’t quite got the same feel of it because they weren’t at all the education events."

"[T]hey’ve left us with other admin people who don’t understand it as well, because they’ve not been with us, so basically the administrators are different now. And I, we don’t have the same relationship that we had with the original ones."

“[I]t seems to be people will only do it for about a year, it’s not interesting enough, because a lot of it is about strategy and local things, and they can’t really do anything, there’s nothing that they can do with it."

"[G]radually people just drop out, because there’s nothing much to get their teeth into ... [T]he groups were quarterly and they’re now supposed to be four-monthly, because we thought we could manage that, but now really, with the way we haven’t been able to organise meetings, the next meeting’s in early December, so yes, it’s difficult." *[GP 3]*

- 1. **Work in large impermanent teams** (“Motley crew”)

*Importance of fairness – in contribution and benefits*

“[T]hat’s been discussed a lot during the course of the project ... the issues about time spent inputting to a community system when we’ve got our own stuff to do.” *[Palliative care professional 1]*

“[I]t’s got to work for us, it can’t be that it’s just a tool we’ve got to use.” *[Palliative care professional 4]*

*“Politics” and conflict*

“There was a lot of conflict that needed to be managed early on, which I don’t think anybody handled particularly well and that has caused delays in the project.”

“I wanted to go and see [name] and her to run me through [clinical IT system] and three failed promises, so I stopped there.”

“I do think sometimes that the Stakeholder meetings, when we’ve got representation from several of the acute hospitals … it feels like they’re pulling, that the acute trust side of things are pulling the strings and dictating what they think they should have. And, ultimately, this is not necessarily about the acute hospitals.”

*Variety of languages*

“I don’t know if people have got it in their heads that it’s not a system … I know that one of the team members did attend a meeting where a colleague of mine had said, ‘You do understand this is not a system?’, to which they said, ‘It *is* a system’. And it’s slightly frustrating, because you think, ‘We’ve had this conversation before!’.”

*Inconsistent messaging even from one and the same sector*

“[H]ow to get [IT experts] working in a totally entwined way and delivering a consistent message, as if the two of them belonged in the same organisation and in the information and systems and IT world, has defeated me.”

*Lack of action on agreed decisions and lack of reliable enforcement mechanisms*

“I’ve included their Chief Exec, I’ve got the Chief Exec involved. They’ve just got other things on their mind. They say, ‘Yeah, it’s a good idea and we’ll make it happen’, but they don’t put weight behind it.”

“[W]e’ve got a whole load of NHS employees [on project team and stakeholder group] and we rely on their contribution as a point of goodwill, all of them can, in essence, refuse to cooperate.”

“[I]t is not a good thing for me to be leaning on people and demanding of them to do it faster or quicker or … better, if what they’d done was not good enough … [N]ot when it’s not their main job.”

“[A]nd, in the end, I have to admit, I gave up and just thought, “well, let the project go on when it goes on, that’s, I’m not going to lose sleep over it, I’m not the project manager, I’m not accountable for delivery of it”. But … it doesn’t sit well with me.”

“[T]hat caused quite a bit of fallout because here we were [project team], telling someone in [organisation] that we were not going to follow their advice and they were certainly not very happy. So that did make life very, very difficult for a while.”

*“Passing on the buck” around infrastructural solutions*

“[E]ssentially, we need a little bit of infrastructure, probably cost 3,000 pounds or something for them [care home] to do that, and that’s peanuts, but that’s not happened yet. I’m still in firm negotiations to try and get them to pay for it, I’ve refused to pay for their internet access line.” *[GP 7]*

### 1.4. IT work, narrowly construed (“We’ve known you for so long”)

*Multiplicity of systems and lack of interoperability*

“The biggest barrier to data sharing is the use of multiple different software packages, it’s just daft, isn’t it ... [Y]ou have a phone call with somebody from mental health and ... they’d say, 'But haven’t you looked on IPAS [?]?’ and I’m like, 'No, because that’s not a software package that we have'. 'Have you looked on [System D]?', ‘Well, no, because that’s not a software package that we have'. So it’s having multiple different IT systems is just not good.” *[Out of hours professional 4, GP]*

“[H]aving lots of different IT systems makes no sense.” *[Out of hours professional 4, GP]*

“[T]here’s no unifying computer system and not everyone’s on [System D] either, so that’s another nightmare. ... [T]he major issue for GP IT is that there isn’t one system, which I just don’t know how that could have happened, but I’m sure there must be some vested interest in IT company ... I’m sure for CCG-wide basis having a single system would make everything so much better.” *[GP 7]*

“It’s a shame ... we’ve just got so many different software systems in the region that ... [left incomplete]. We should all be on the same, or at least one that speaks to each other, because in this day and age of modern medicine ... we still can’t communicate electronically, [it] just seems bizarre." *[Out of hours professional 1, Nurse]*

“[T]here’s a number of issues, both IT issues and finance issues about making the two systems [hospital and community] talk to each other.” *[Palliative care professional 1]*

“[T]he systems still not talking to each other, I think, is the biggest hindrance for patient safety.” *[Out of hours professional 1, Nurse]*

*Technically enabled integration is not necessarily meaningful practical integration*

[Software 1] is a triaging software, it’s not your note keeping, and the whole point of [Software 2] is we want to be able to see the notes.

[T]hey [call handlers] are writing the odd free text comments, they’re writing the [Software 1] bit that in the end gets pushed into the case ... but we’re using [Software 2] for the case handling.

[B]ut what happens in the case managing, how they’ve set it up for us in 111, which if I’d known about as [role], I would have stomped my feet about, and we’re trying to stomp our feet about at the moment, is that once they’ve gone into [Software 1], if they then try to come back into the clinical note, they can’t see it. So that means if it’s coming up saying, ‘Dispatch ambulance’, and we go to try and see preferred place of care, we can’t see it.

*Unresolved challenges around mobile working*

“We have had a trial of … recording via [mobile working tool] to upload the information into [System D] and we were probably one of the most successful areas in the country … But there were technical difficulties with that project and after nearly ten months of trying to get it to work, we had to stop the project. So now we are looking at alternative mobile solutions.”

“[W]e have used [mobile working solution] with the rehabilitation staff, but it doesn’t work 100% smoothly … Essentially, they have to download their caseload first thing in the morning, then they can update the records remote and mobile, and then they have to upload them and synchronise them with the main record at the end of the day. The problems come when you lose signal as you’re doing your downloads and uploads, you find you get frozen records and lost records. So it’s not robust and we have to contact [System D] to resolve all of these stuck records.”

"[I]t is a very good piece of mobile technology but to ramp it up, the capital investment would be just huge and prohibitive, because these are about double the cost of a standard laptop and for junior staff, they don’t need that full access to the full [System D] record. For example, a phlebotomist … she just needs her visit run and … to be able to say, ‘yeah, I’ve done this address and I’ve collected my blood samples etcetera, or done the blood tests and these were the results’ … [W]e still have not resolved what mobile working solutions for which levels of workers."

*Persistence of some old clinical IT systems and software more broadly*

*[no longer an issue in the locality]* “[T]he data sharing electronically hasn’t really happened here yet because we are on [System A_2_], so nobody can access our data. So that looking into our patient record isn’t something anyone’s done yet. We share our data more by the, ‘we’ll send a patient record and summary and the template etc., to out-of-hours’.” *[GP 1]*

*Key settings lagging behind with the implementation of comprehensive IT solutions*

“[I]t wasn’t that long ago when [organisation] were going to have [System D] throughout their whole organisation, I think by the end of 2013. It then slipped to the end of March, and then it was around the end of November, maybe mid-December … that [name] announced that it was highly likely at least two of their patches wouldn’t have [System D] by the end of this financial year because there was no money.”

“And if [the acute trusts] haven’t got their act together to get [module], we’re onto a lost cause. I know only [hospital] missed out on that, the other three got their act together. So there’s contextual bits there as to why it’s all so difficult.”

*Lack of even basic infrastructure in some settings*

“The hospices, I’ve asked them all to supply us, if they wish to and feel are able to, to supply us with an nhs.net address that we can put into the system so that when we want to send them a letter, we can send it electronically to their nhs.net email. But that’s not an easy solution for hospices and, can you imagine, because somebody’s got to be monitoring that email account, and looking at it, and checking if anything comes into it. So if you haven’t already got that sort of thing set up in the hospice, you’re going to have to set up not just an *nhs.net* but the process.” *[Palliative care professional 1]*

“Working with a big nursing home that doesn’t have computers on the wards, doesn’t have internet access on the wards is a real headache and something I’ve been, for the last two years, trying to agree for them to do and it’s just not happened. They’re just not willing to spend the money on an open internet line, which we can then form secure entry connections through and take a laptop round with us, which would be brilliant!” *[GP 7]*

*The presence of pockets across the health service where paper notes still persist*

“[T]he medical admissions into [hospital], it’s all written down on paper, they’re still on paper records. Even for prescriptions, it’s pretty much all paper prescriptions, and I find that really strange. Because when I was a House Officer 26 years ago, we had electronic systems in [location] then for prescriptions and results. They’ve got it now for results and imaging, that’s electronic, and I just cannot believe [it]. And so there’s a data sharing aspect of the secondary care that’s so far behind. ... [W]hile you’ve got that mismatch, how can it be a smooth system? Because whatever you create electronically, they’re still on paper.” *[GP 1]*

“[W]e have a patient who moves area, so they move out of a [location] practice using [System D] to our practice, and the records don’t come electronically. So their practice literally print inches thick of their records from the [System D] and they post us, via some central organisation, inches worth of paper, which somebody in our practice then has to summarise and put it back onto our system.” *[GP 4]*

*Generic signal issues*

“Sometimes you are in the car and the mobile laptop, the connection fails periodically, so you’ve got to worry about whether you can even get connection and then ... you tend not to bother, but you tend to just go into the house and have the consultation, and try and gather as much information with those family and relatives, which is a shame because you can duplicate, which is burdensome to the family.” *[GP 3]*

“But we are slightly out in the country here, we don’t get very good 3G connection, don’t get a 4G connection at all.” [GP 7]

*Limitations of generic IT skills, particularly in some staff groups*

*[this is highly likely to have changed since data collection, particularly after the COVID-pandemic]* “[D]istrict nurses, many of them are still using paper records, so the very fact of putting stuff on a computer is a novelty, and some of them being people in their 50s and 60s, computers are a bit less familiar … [T]hat’s a much broader cultural issue of community nurses.”

“[I]n one of the teams … we had eight nurses who had to do the basics of getting round your keyboard: they don’t email, they don’t attach documents and that sort of thing, so we are talking the fundamentals of IT skills for some nurses … [T]here’s almost no other staff groups where you find that basic level.”

*Limited understanding of record sharing*

“It is complicated and you do think, 'no wonder it’s complicated to the patients as well, and how are the patients ever going to be, have it explained to them when the staff and the clinicians don’t get it?!'. So you can really see why it is difficult. I think because there are so many consent type things and they’re all dealt with differently, it makes it more difficult.”

“There’s then the main record, and that, of course, is then dependent on whether the GP has shared out or not, and whether he even understands whether he shares out or not, or whether their locums have just clicked on the sharing screen and they’ve just clicked through, or whether somebody has clicked on and said 'OK' and therefore actually set it, but hasn’t actually known that they’ve set it, okay?”

“So you see lots of what look like GP records, including prescribing, but it’s not GP records, it’s actually a patient being dealt with in the minor injuries unit. You have to be techy enough to have spotted the little blue icon which means that this is a GP record that has been shared out.”

*Impossibility of convening a project team where all members have high level of IT skills in addition to their primary expertise (e.g. in end of life care or project management)*

“I very specifically put into my email, ‘I am there to do the underpinning knowledge, I am not IT-minded at all, so you can ask me a few questions about the IT and I’ll be able to answer them from what I’ve picked up, but I am not an IT brain’.”

“I know from meetings where [names] have gone to … demonstrate the Share my Care template, and, unfortunately, it was understood to them that it was a magical system … and [GP practice], who are very competent [System D] users and have been for nearly eight years, just laughed, laughed them off and just said ‘this is crap, we’re not using it’.”

*Dynamics, variety, complexity and unevenness around data sharing means risks can be difficult to identify and manage*

“Any other things to particularly change? [Pause] I suppose the understanding of how the different IT systems, EMIS and EMIS Web work and that interface between [System D] … that updating of information between the EMIS and the [System D], because community staff and out-of-hours are updating always the [System D] side, the GPs are always updating their EMIS side, and it’s that continuous process of making sure that each other has the latest information. And I think that’s very complex and there are a number of risks linked with that and we’re all struggling to bottom out how we manage those risks.”

*IT systems as “work in progress” – may not live up to their promise, are not improved upon fast enough and ultimately fail*

“[W]e’ve paid lots of money for [solution], but it doesn’t seem to give us what we want, and we’re still having to make endless phone calls to GP surgeries to ask them for information, because, again, it shows you, I think, the last couple of appointments, the medication and maybe the main diagnosis, but it won’t show you details going back over time.”

“I haven’t personally seen it, but it is a big issue because the CCGs have put money in to have this system for the healthcare team and occasionally we do get a bit of complaining from board members about why are we phoning. And the reason is because it’s not doing what we thought it would when we paid for it. But if it did work, then it would be great!" *[Out of hours professional 4, GP]*

### 1.5. Rules, laws and algorithms in spaces also calling for humanity, flexibility and art (“How to Repair a Broken Human. A Manual for IT Systems”)

“I do sometimes feel that … the IT side of things means that we lose sight of the patient because the codes won’t allow us or the system won’t allow us and … it becomes, I’m sorry, but yet another piece of IT work, and we know how successful IT work is in the health service.”

“And he rang me within a few days and said, ‘My life is transformed, I can’t believe it, I’ll be able to go to my daughter’s wedding, I’ll be able to do all these things!’. And you think, ‘Gosh, to be able to make that happen at such an important time of life!’.”

“So it’s hugely rewarding, just those sorts of things would happen when you’ve been having a particularly awful morning with either computers or paperwork or management or something, you’d suddenly get a phone call like that and you just think, ‘Wow’.” *[Palliative care professional 5]*

### *Mismatch between the IT representation and the clinical or practical reality*

"And it’s often just a bit annoying, because you can’t get into the notes until you’ve consented ... [N]ormally, you like to open the notes, have a quick look and call the patient in. Well, you can’t, because you have to consent before you can get into the notes. So I find that quite frustrating, but some of the receptionists that are better take the consent when they arrive, so then you don’t have to do it again." *[Out of hours professional 1, Nurse]*

"[A]s that screen [for asking for consent for sharing] flashes up, the locums, and I suspect even the principals, I suspect are just ignoring it, and so the chance to be asking the questions individually. And then sometimes they may just click 'yes', in which case it never shows again, and then they might just click 'yes' and say, 'not sharing it'. ... You can open it up and change what the patient wants at any point, but if you’re not being shown that screen, you’re not going to do it."

### *Use of unsafe workarounds*

"Our call handlers have to tick buttons and say things like, 'No clinician available', when in fact there is one, 'Put into queue', when in fact they’re not going to be put into queue. Those calls are only going to be warm transferred, and so they’re having to click buttons that say the exact opposite of what they’re doing.

It’s a way of forcing the software to do something that it’s not designed to do, and we can do it, it works, we’ve worked it through, they’re doing the same at [location]. But it would be much better that the software was rewritten in a way to make what we’re trying to make it do is actually what it says on the screen."

"What could actually happen is that they pass, one of the first ones that was passed through to me, the way they did it, the case was then closed. So I was now speaking to a patient, but I didn’t, well, I did know who they were and I knew what the problem was ... but I couldn’t see their notes, and I had no notes to write into. And this was one that was an ambulance transfer service, that potentially was a call I wanted to take very quickly and then [involve] the ambulance service.

So in part of my instructions I got, 'This is how you reopen a case quickly', which at that point I didn’t know. So I actually created a new case from scratch so I could deal with the patient, which is something that my clinicians wouldn’t normally do, because the case has already been created by the call handler, and it still takes them about two minutes to find the demographics and create the case."

*Mismatch of personality types*

“[T]he typical type of personality … that goes into nursing practice is a very people-focused person and they tend to improvise a lot, they won’t follow rules as in ‘the IT process says you must do it like this’ …[I]t’s been challenging for the IT guys to understand the nursing perspective and the nurses to understand the IT guys’ perspective because they’re just opposite types of people and that’s a huge generalisation, isn’t it, but ...”

*Rationality of the IT tools revealing potential irrationalities in the system*

“[T]he discharge letter goes to the GP which tells them what drugs they’ve been sent home with, including the just-in-case prescription. Then if it is a patient who we expect will need to use their just-in-case medicines from that day on, we will personally refer to the district nurses, but if it’s more of an anticipatory prescription, we will then refer onto the community Macmillan team. Then at the time when it becomes necessary for them to use just-in-cases, they will get the district nursing team involved or the district nursing team would somehow get some more because the GP has contacted them.” *[Palliative care professional 6]*

### *(Broader than IT on “Rules, laws and algorithms in spaces also calling for humanity, flexibility and art”; not in the framework of the text)*

*The human, caring side of healthcare delivery feels to have become subordinate to legal considerations*

"I just can’t believe that the human interaction, the human caring side of healthcare delivery is now so expunged by the rules and regulations that staff have to follow, which again is, I feel, very much driven by legal consequences and this sort of thing of them not doing it." *[Out of hours professional 3, GP]*

"[T]he staff are frightened of stepping over the mark, they’re terrified if they do [not] resuscitate this person, even though there’s a black-and-white copy of the do-not-resuscitation thing *[Note: as opposed to a red-rimmed one, which is the standard]*, that they will get hauled over the coals for not doing this, that or the other or will get themselves into trouble. So they have to just do their little bit. But it can be entirely inappropriate and, worst of all, patient dies on the way to hospital in the ambulance, which has happened to one of my patients, when I thought I had it, I thought I had it all signed, sealed and delivered. But no, there’s a little gremlin somewhere [laughs]." *[Out of hours professional 3, GP]*

“[W] went to see a lady who was near the end of life ... and I looked for the Do Not Resuscitate form … and the only form we could find was a blank one. … [Y]ou’ve not got that information to hand, so actually you need to start CPR on that patient.”

“Even though clinically I would know the right thing is to make her comfortable and leave her, in a court of law, you wouldn’t [stand up], if the relative turned round and said, 'she died and you didn’t do anything', it makes things a bit difficult.”

“Well, there are some nurses that would say … because the lady was really poorly, I was thinking, in my head, 'we have not got the right paperwork to support our decision making here', and her thought was, 'well, I’m not going to do anything even if she does' ... It’s difficult, isn’t it, because it’s not in the best interests of the patient or the family or whatever, but the law is the law! Isn’t it? I wouldn’t want to lose my registration!” *[Out of hours professional 5, Nurse]*

**1.6. Core tasks – peripheral tasks** (“Mixed Abilities Class dominated by a No Abilities Group”)

“[H]opefully, if our GP or district nurse colleagues have had that experience of a surprise answer [of a patient's preferences for end of life care contradicting their expectations], then they’ve realised how incredibly helpful it is. But I know quite a lot of them do like the specialist nurses or people from the hospice to have those discussions.” *[Palliative care professional 5]*

“Because I’m not in mainstream general practice now, I don’t know what the understanding of it [data sharing] is out there ... [P]art of the problem was poor GPs have got so much to get their heads around that they’re probably not bothered, and if they’re sitting in their own little world, it doesn’t bother them too much.”

“We know in our hearts that there’s a lot of GP’s that don’t really care about End of Life Care. It doesn’t float their boat.”

**1.7. Working with highly sensitive, emotive, incendiary issues** (“The tabloid press”)

*GP 2:* “I was against the national data sharing scheme, the recent one whose name I’ve forgotten, because they, the Government were lying to us about the fact that the information was being [pause] sold to commercial organisations.”

*Interviewer:* “Care.data”

*GP 2:* “Care.data. I didn’t like the fact that they were lying to us.”

“Care.data did worry me slightly ... research is very important and [I] want ethically approved research to be able to access that completely anonymised data, but the concern is about private companies have high access to it. And I think that’s a genuine concern and one I’m not fully clear about at the moment and I feel that’s terrible, because I’m supposed to be advising people about it!” *[GP 7]*

“[W]e hit a quagmire of IG issues, neither of which we’d really clearly foreseen … [W]e took about twelve months getting past those hurdles before we could really get going and that was pretty dispiriting … [W]e were the first data sharing IG problem for [name], so that took her time and … with the care. data that’s all exploded.”

*Interviewee 1*: “And I think it’s media propaganda.”

*Interviewee 2:* “Yeah.”

*Interviewee 1:* “That stops people.”

“Like you [other interviewee] said, lack of patient education. You have the Daily Mail, don’t you ... ‘Tesco’s staff will be able to see your [data]’ and then people, ‘Ooh, I’m not doing that!’. And there is a massive lack of understanding out there in the general public of what it’s actually for." *[Interviewee 1 – Out of hours professional 1, Nurse]*

“Maybe it’s bigger than this, maybe it’s bigger than the local. Maybe it’s the demoralisation of the collapse of the Liverpool Care Pathway, and many of us that thought that it was a good tool … And the adverse media attention and the misuse of it perhaps in hospital, the district general hospitals, by untrained staff and poor communication, it’s rather given it a bad name ... [A]dvance care planning has got harder since that publicity.” *[GP 3]*

“[End of life care] is so emotive and people have all sorts of personal reasons for not wanting to engage with that.”

“[It’s] complex and subtle and even tiny differences in how you phrase something – like, for example, in the DNACPR teaching we’ve just been doing, where we were talking about whether one says, ‘futile’ or ‘no reasonable chance’ or ‘only a very small chance’ – all of these result in entirely different outcomes and responses from people ... [I]t’s an art, these conversations, not an exact science.”

“I’m afraid, very afraid actually, and I use that word meaningfully, that ... there will become a tick box approach to needing to have advance care planning conversations in a way that will be really unpleasant for some patients.” *[Palliative care professional 3]*

**1.8. Technology – human users** (“Humans vs. Machines”)

“[I]f you go for the last three entries, say, you phoned them up and couldn’t get through to them and you write, ‘unable to get through on the phone’, and then one of the dispensers says, ‘message sent to doctor to check review date’ ... you can end up with loads of ‘administrative offal’ basically, which has no real clinical narrative in it ... and that blocks it up.”

“[Y]ou can try and say, ‘I only want ... clinical events which are a face-to-face thing from a clinical member’, but quite often doctors will not change the thing which says where it occurred ... [I]t’s not a smart system, it’s just doing what you tell it, to check what the last three things that get dragged in are. *[both quotes GP 7]*

“[L]oads of people are generating data but we don’t have the discipline, and we don’t have the systems, and we don’t have the culture which sees it as necessary to be strict enough at the point of entry to generate stuff which makes a difference further down the track.”

*Interviewee 1:* “I’ve had not any update in my training in [System D] ever since I started, and you get the updates and changes, but who reads them?!”

*Interviewee 2:* “Me.”

*Interviewee 1:* “You are a geek! ... I rely on people like to tell me then basically” [laughter]. *[Interviewee 1 – Out of hours professional 1, Nurse]*

**2. PURE DRIVERS**

**2.1. Pure drivers internal to the IT solution**

*Over 60 (potential) benefits identified. See manuscript for quotes on the most frequently mentioned and most impactful ones.*

*Nature of domain*

“[End of Life Care is] slightly different from everything else where we’re looking at access rights to a patient record, in that people just want joined-up care, so it doesn’t get messed up when you only get one chance right at the end.” *[GP 7]*

**2.2. Pure drivers internal to the project development and implementation team** (“Dream team”)

*Positives of the Clinical Lead*

"[O]ne of the greatest strengths is, obviously, [name] and his leadership of the project. He has a very calming and considered way of working. He’s always very validating of everybody’s contribution to the project and in that way he’s really got a good team around him who are all really positively motivated towards delivering this.

I mean, [name, other]’s role is good because he seems to sort of do a lot of the organisation and administration behind the scenes, but I think [name] takes the thorny issues and he deals with them head-on so they don’t become barriers, but he does it in such an affirming way with all of the people that he works with that he is managing to carry this project forward.

I think if it wasn’t for his leadership, the politics could be getting in the way and we wouldn’t be delivering this project as well as we are, but the fact that we’ve delivered in [pilot LCG] and now the rest of the county are asking for Share my Care is just absolutely testament to his leadership of this project."

"[W]e had proper, true, clinical leadership from the outset with [name] … in many ways we’d see him as a senior responsible owner, and need to involve him when the world’s going to end and there are real problems with the project or areas that we said, 'We can’t resolve this, we need you to give us a steer', and having his involvement on the ground, really on the ground of the project, has been invaluable."

"His strengths lie around the fact that he is passionate about End of Life Care. He’s very well educated and researched and trained in it. Best I can tell is he’s also very well connected. I also feel, my judgement is that he’s got integrity and I don’t necessarily see that trait in a fair number of people who end up serving in positions like he is, on a chair in a Programme Board. And he has been prepared to put a lot of his own personal time into this."

"I certainly don’t think he’s ever ducked an obstacle that was within his domain and he’s put a tremendous amount of time and commitment into the project. But the measure of success is going to be how many GP practices create, use the template, create plans and summaries and then share it."

"[Name] as a, as a chair is very approachable, everybody knows him, and very likeable, so people quite like to have him around ... he will communicate something and people will respond."

"[Another strength is] [name]’s very clear vision of what he wanted to make work and he knew that, he obviously saw what the benefits could be, with his background in general practice and out-of-hours work. So enormous strengths in that way, yeah. I think it’s a project he very much needs to be proud of."

"[Name] is very inclusive, included us in the project meetings right from the start."

"[T]he project leads like [name] are pretty switched on and willing to listen to advice. And I think that’s one of the key strengths"

*Clinical champions for data sharing and service development more broadly*

"[H]ow we work nowadays is when you have strong clinical leadership, and GPs really, really driving something, it works really well, as obviously also there are areas where things haven’t worked that well, but it does help the agenda and went down well with the GPs."

"[Name] is a natural champion. If he believes in it, he will champion it and shout from the rooftops. Whereas there are other people who are far more reserved and so [name], he won’t jump up and down about much. He will just work diligently and in a trustworthy way and do his lead by example and do it himself, but not necessarily vocalise it and kick others. But then [name, former], as the character that he is, is also in a formal position of leadership, whereas [name, latter] isn’t."

"[Name] … as an [System A] user was championing the using of [System D] for this project that we were doing. I certainly didn’t think of it at the start of the project, the way to make this project fly is to find a non-[System D] champion to say 'this is the way to go'."

*Good variety of professional backgrounds and organisational roles on core project team, with members being of high calibre in terms of their knowledge, skills and expertise and also having sound understanding of local structures.*

"[W]e’ve got a good group of people with different knowledges to be able to … make the best of the project … that’s been a strength."

"[T]he strengths then, I think is the fact that there is a mixture of clinicians and non-clinicians on the team."

"[Name] became involved first and foremost because – although she’s only a practice manager, and by that I mean she’s not a GP – she happened to have the End of Life Care Lead for [location] … The fact that she was a practice manager rather than a GP … we saw as an advantage because she saw a bigger picture for administrative processes."

"[T]here has been a dedicated resource around it in terms of support, IT, technical support, strong leadership - [name] generally speaking, [name] for us locally, yourself [researcher], [IT person], so there’s been a very strong group around it. And that means that whenever we have little hiccups or just extra communication, it’s all been fed in and the response has been quick."

*Poignant personal experiences of end of life care of some team members*

"It’s a strength that [name] understands where we’re coming from … [I]t would have been very easy to have had a project manager who, yes, had experience of managing these types of projects, but really didn’t get it because there’s a lot of people out there who don’t get what the fuss is about with End of Life Care. But I know [Project Manager] has got a very personal reason for making this work and getting this done … [which] keeps the team moving in the right direction."

"[P]robably if I had not had a wife die next to me in bed knowing it was what she wanted and not having had discussions with her about a lot of things close to her heart and mind, then I’d never have stepped into this type of ring. But then I would never have been asked to do it because a big part of why [name] would have chosen me, I believe, is that I had a particular life experience that, when you added it to my skills set, made me credible. And without the life experience I wouldn’t have been as credible and, collectively, could probably have found someone better."

*Committed, proactive, competent and determined individuals working on the project – comments about other team members, expressions of own motivation and commitment, and comments by external people*

"What I wish we had done is much more actively sought out [name]’s team's expertise much earlier, because she’s been absolutely superb. And it was months before we realised that she might be available to support us, although we’d heard her vaguely talked about as being somewhere up in [city].

"[T]o be having such cheerful colleagues willingly going the extra mile in pretty adverse circumstances is just a testament to the quality of the individuals involved, that everybody has got the bigger picture: this is about improving care for patients. And people could have retreated into silos and been difficult and yeah. Yeah, it could have become highly dysfunctional on numerous occasions, but people have been gracious with the bigger picture."

"[A] bit like [name] said in an email many months ago, ‘I don’t do can’t do!’."

"[W]e’re in this period of change, we have the ability to change … we have the opportunity to look at that, if we’ve got time, of course, to try and make a positive impact on both how we function as an organisation and use IT in a way that is going to make a difference.

"The End of Life Care Dashboard … that’s one of those areas that I’m very excited, I’m very motivated to move forward and deliver."

"I just feel it’s very much a group of, “We work for a solution, let’s find a solution, there’s a problem, there is [a solution]” and that’s very refreshing, I must say, I think it’s been very good [laughs]. ... [I]t would probably look different had it been a CCG-run project, because ... the funding would have looked different for [36:32], but also I think the [human] resources ... there wouldn’t have been the same level of people."

“The project's group’s been really, really helpful in accommodating how we can implement in [organisation], largely because our key workforce are the district nurses and they are so under clinical pressure that implementing change with them has to be very carefully thought about.”

"[W]e’ve had some very, very difficult times, when it has looked as though the whole thing was going to go pear-shaped and actually we just plodded on."

"The secret has been fairly dogged determination, not give up, and I can understand why many areas of the country may have given up, because it does require persistence."

"[W]e’ve not given up and we’ve kept popping, sticking our heads above the parapet recurrently, and we’re clearly an initiative that’s not just going to be here today and gone tomorrow."

“[T]he most significant step was when we steadfastly resisted and said ‘No’ to the original advice of … using the Summary Care Record. That was what the first IT systems person within the CCG recommended and we had a considerable difference of opinion.”

"I rang up … [location] who in the Ipsos MORI poll had … an evaluation of their efforts, and they were one of the places that had used the Summary Care Record as a platform. Spoke to their project manager and he said, 'you’re absolutely right to dig your heels into the ground … we are about to fold because we regard our project as a failure, although we might not wish to present it like that to the outside world, but we are stopping because we cannot engage GPs because they do not want to be responsible for all of the updating'."

"[I]f somebody wants to see me, great, I will move heaven and earth to get there and see them, but if they don’t, I might try and encourage them a couple of times but I’m not going to keep banging on the door. ... You’re not going to make any friends or influence people by making yourself a pain in the neck and, sadly, rightly or wrongly, that has been the approach that’s been taken in [area]."

"I don’t think this has been about any one particular group of individuals, this is nobody’s vanity project … it’s just about getting it done."

**2.3. Pure drivers at the interface between the internal and external** (“Invisible threads”, or “weaving a spider’s web”)

*Fit with and work on adapting features of the IT solution to a variety of features of the external environment*

"Most of [the practices] have chosen to [use IT team tools] because ... someone else is maintaining [it] on your behalf, particularly around the QOF [Quality and Outcomes Framework] stuff ... [W]e always keep ours bang up-to-date, earlier than the clinical system suppliers do.”

"[We] try to join things together with [location], because most of the elements that were trying to be captured were the same and trying to, which is our general aim across the CCG, is to standardise the way things are recorded, and End of Life was another one like that. ... [W]e saw Share My Care as a part of an ongoing programme of work around End of Life recording, but, of course, it introduced the concept of the sending the summaries to the out-of-hours."

"Some of this will just be trying to key ourselves into the information management strategy of the CCG … but understanding, I guess, what we’re delivering over what timescales and then marrying that up with the information management strategy and … any other initiatives that we have."

"I think, what we were told by the company [was] that, ideally, 70% of it stays the same and we only change 30% of it. I’ve no idea how much we’ve changed! [Laughs]... [T]here was a lot of work to adapt it for the NHS in the first place, including terminology and spelling, and we haven’t been doing that, but the company has made a lot of adaptations. For example, there’s an awful lot of software in there that talks about how you bill people, which we don’t have to do ... but we do have to have a different component of the software for tracing the charges that the CCG would want to know about."

"[W]hat was pre-existing on the system about palliative care was very, very medically orientated and ward-based interventions and so I looked at that thinking, ‘can I use any of this?’ and most of it, I thought, ‘that is really not what we do here’, so I just started afresh ... [I]t has made me realise quite how different palliative care in the States is."

"[T]he software has been purchased from an organisation in the States ... and those of us who are application analysts have been building or amending that software to fit the work flows and the processes that happen here."

"There isn’t a person, an application analyst for every single department, so we’ve done broad areas where we have the most knowledge and then other areas where we’ve gone to consultants, the clinicians and say, 'explain to me what you’re doing at the moment, I need to know how you do it and what information you need and then I’ll go and make it and then I’ll come back and show you on the computer and see if it fits what you need'."

"Anything that I built for a speciality also has to link with all the other specialities in the baseline ward, nursing ward, doctor information. So you’re using some of the pre-set software that’s set up, but then you’re adding on bits and changing bits and fitting it all together."

- 1. **Pure drivers external to the IT solution and team, but internal to the broader health IT ecosystem (“Catching a wave”)**

“[W]e’ve been fortunate in that we have ridden the crest of a wave in the CCG, and possibly in the wider NHS, in terms of initiatives for greater data sharing. Partly we may have catalysed some of that, but I think we’ve also ridden a wave generated by other people such as by [name] and then by the MDT co-ordinators.”

“When we set out, it needed a special name because it was a special project and it was a special thing to be doing. It’s now boringly normal.”

“I think I have been quite sensible and sat back and let people like [name] drive, I’m damned if I’m going to make the same effort, it would be the same thing, let him trailblaze and then we’ll follow.”

“Just last week I had one of the consultant oncologists here … and he somewhat timidly said to me, ‘You know, I gather that lots of you GPs out there are using [System D], do you think there might be any way and would the CCG possibly approve if we were to arrange to get access to [System D]?’. And when I bit his hand off and said, ‘I’m desperate for you to get access to [System D]!’, he couldn’t quite believe that we weren’t going to be difficult but for my response to be, ‘this is exactly the way we want things to go’.”

“I think the benefits need to be sung about.” *[GP 6]*

“[T]he positives to me are overwhelmingly, the case is cast in iron, it’s a very strong case for this, but I’m mindful of the challenges and barriers” *[Palliative care professional 3]*

“[M]y take-home message is, the more we share, the better it is for patients.” *[Out of hours professional 4, GP]*

**3. OPPOSITIONAL AND/OR AMBIVALENT FORCES**

### 3.1. Oppositional forces, temporary (“The race is on”)

*While beneficial to data sharing in the long term, concurrent transformational initiatives may be highly problematic in the short term (e.g. transition to an electronic patient record in some settings)*

"[R]ather like [clinical IT system] being a necessity here, the data sharing project was something that just had to happen. And in a way these two big IT changes happening at the same time is very positive, because the way of working that each of them brings will help the other one ... they’ll be synergistic."

"Yes, except, of course, they oppose each other at the moment, in the sense that people are just lacking the time and energy to be involved in a system which isn’t the primary one they’re using, when the primary one is so challenging. But it really will improve." *[Palliative care professional 3]*

"[T]he combination of initiatives, I think, do make sense, but it has been really hard work to implement those changes and we have had a lot of resistance and a lot of tears along the way because the scale of change is quite dramatic."

**3.3. Ambivalent forces, legitimate differences in clinical contexts or contexts requiring clinical information** (“All in the open or skeletons in the cupboard”)

"The template is used to update, so if the patient was deteriorating and then needed a change in medication, we would add that onto the template, but just in a line or so ... the greater detail is put in the journal entry. So again we were told it should be short and sharp because someone’s got to read it quickly, out-of-hours would want to see the basic information." *[Palliative care professional 2]*

"[W]e will see the patient for the first time and then we have our own specialist palliative care template, which has got a lot of our national data collection type stuff on it. This is where some people have struggled as well because we’re asking them to complete our own template and then to go on to complete the End of Life template. But it does have slightly different things in it, it’s there for a different reason." *[Palliative care professional 2]*

“Our colleagues in the community initially were a bit sort of wanting to use both and we’ve had debates in the team as to how ... and we said, 'come on, we’ve got enough to do, we don’t need to be filling in another one'. But I try and use this [project EoLC template] ... sometimes I find myself writing in these boxes just in order to make sure it’s documented for my own team in the community ... I’m using it [project EoLC template] to replace this other one because I feel that it’s duplicated, but I perhaps, I definitely write more than [trails off and follows an association to another topic]" *[Palliative care professional 2]*

*Interviewer:* And you stopped using your original template?

*Interviewee [Palliative care professional 2]*: In the hospital team we have, but not in the community.

“Some of the templates [of district nurses] are far too, you know, do you really need to know that they were with them for 4 minutes, or it was a 4-minute entry, no, we don’t, and we may well have to scroll another page and a half and read through a load of rubbish. But they obviously need it for audit and for purposes their end, so you have to just read in-between the lines sometimes.” [*Out of hours professional 1, Nurse]*

**3.4. Ambivalent forces, divergence of values or complex entanglements** (“One man’s meat – another man’s poison”; “You don't find roses growin' on stalks of clover”)

*Conflicting perspectives (and the same thing appearing as a challenge or driver) towards features of the data sharing tools and even the foundations of their existence*

*Value of planning (a) vs. downsides of planning (b)*

*Both a) and b)*

“[I]f you’re faced with a very clear plan from an organisation or somebody you trust and know, which has the effect of ‘what happens between W and Z’, it’s pretty straightforward and you save a lot of time. But plans which are not short enough, or specific enough, or can’t be related to the context that you’re presented with, inevitably mean that certain amounts of the work have to be done again."

b)

"[I]t’s possible to be unrealistic about how much pre-prepared plans can short circuit clinical decision making, given that the individual commissioned at the point of making decisions is responsible for whatever that action is."

*b)*

"[O]ne of the troubles is that life doesn’t follow guidelines and so [laughs] we’ve spent time doing some of our reviews of the plans for patients and then, blow me, something new will come along that you couldn’t possibly have predicted!" *[Out of hours professional 3, GP]*

*b)*

"[T]here’s a lot of people who we are sharing data, doing care plans on, who are elderly or vulnerable, are at risk of things, but don’t have anything particularly helpful for us to pass on. We’re sometimes struggling to come up with something that we can put in a care plan that isn’t ‘recommend annual flu vaccination’, ‘come for regular check-up’, whatever, that’s actually helpful for anybody!" *[GP 4]*

*b)*

"[T]hat makes it probably less efficient than you think it might be, because it’s quite hard to sit down and make an advanced plan for all the different scenarios that you could possibly imagine, although people go through the same loop time and time again, [there are] all those important facts from their history which might be considered."

*b)*

"[P]eople are looking at the plan, they’re not looking at the patient and the context of the patient, which a very simple vulnerable patient system did quite nicely, [with] the GP just copying a letter or sending a letter to [out-of-hours service] and [them] putting [it in] a file and then the GPs providing out-of-hours care could see that information." *[Out of hours professional 3, GP]*

*The information can be obtained more efficiently in ways other than data sharing (a) vs. those other ways are (invisibly) burdensome (b)*

*a)*

"[P]atients who are fully engaged with their GPs understand completely what’s going on, whose family are all completely sensible will give us most of the information that’s on here and so I won’t be looking to [left incomplete]. It’s those who think, 'oh I’m not quite understanding this' or who say, 'no, I never see my GP' or 'the GP said something', where you’re thinking, 'actually I need more information from the GP'. So it’s not always necessary to look at that or speak to the GP to get the right community information, if the patient’s sensible." *[Palliative care professional 1]*

*a)*

"[M]ost of the information I’ve had from, if I’ve been dealing with a patient [at the] end of life, I’ve had from the carer or from the relative, so you do get the information, but sometimes, or district nurse … yeah." *[Out of hours professional 5, Nurse]*

*b)*

"[W]hen you’re stressed out, those things you forget, and you come away, ‘Oh, I should have said that’, whereas if it’s all shared, when you’re in a time of stress, you haven’t got to keep going around and saying it all around all the time." *[Recent carer 05]*

*b)*

"You just look like a parrot all the time! You can’t remember everything!" *[Recent carers 02-04]*

*b)*

"[T]hey were asking us all the same questions again! And we didn't have the answers. All I kept saying was, 'He's got cancer in his lung'." *[Recent carers 02-04]*

*b)*

"You had to keep repeating and repeating, wasn’t it? .... And going through it and being asked all the questions, and then it started getting confusing because you’re being asked dates you can’t get from the top of your head, can you?" *[Recent carers 02-04]*

*The information is already shared (a) vs. having a summary is better (b)*

*a)*

"[I]t seems a bit bizarre to me that people have to write these separate templates when all of the information is on [System D] and it’s just the way that you view it that, I don’t really understand why we need to ... do all of this because, basically, it should be on [System D] already and it’s just the way that people view it." *[GP 2]*

*b)*

"You could argue that so long as the relevant information is captured in some way on [System D] or in a shared system, that’s what we need. The beauty of having it in one area is, obviously, that it’s much quicker, you don’t have to spend time reading through everything." *[Palliative care professional 5]*

*Perceptions of a) benefits to “allowing access” to own record and “relinquishing control” vs. b) lack of clarity as to who owns the patient record, with the consequences this has for professional independence and control*

*a)*

"[C]ertainly for End of Life Care, for vulnerable adults, for coordinated care plans, I want a single record ... I would be willing to allow the ambulance service to look at the DNR coding that we’ve got on [System D], just as we do with the out-of-hours GP service."

*a)*

"[N]ursing homes should have internet access, we should be able to bring up [System D] there and I would be willing for nurses to be able to access things like the repeat template section on [System D] perhaps active problems so that then we could streamline repeat ordering."

*b)*

"I go on trust, I suppose, a lot for that, but it does feel slightly that we are losing control of that data a bit. I think that kind of loss of control of data is something GPs do have to accept, that we can’t, there’s no way we can work individually, and I’m very happy to relinquish control to other groups or clinical teams. What I don’t want is for private companies to be able to use that for any kind of marketing purposes ... Public Health would have a right to that access, that very valuable data about prevalence, and a lot of that’s already shared anyhow through QOF [Quality and Outcomes Framework], but what I would hate would be any drug companies being able to access it or any insurance and predictors being made on that data." *[GP 7]*

*Conflicting perspectives (and the same thing appearing as a challenge or driver) with regard to the support structures for the project*

*Budget has been (a) generous vs. (b) insufficient*

*a)*

“We’re in a fortunate position that the budget is incredibly generous.”

*a)*

“[W]e’d factored in some monies to support having a dedicated resource or dedicated time to be able to deliver this, however, on the re-evaluation … there wasn’t any resource identified … [so] we had to try and add this onto our existing workload, it wasn’t a planned full project in effect.”

*b)*

“I don’t think we’ve had the resources and the energy to do that [advance work on the Dashboard, a support tool for the project].”

*Perceptions of stakeholder involvement as a) a significant strength vs. b) not entirely adequate, as key groups have been missed; c) not entirely adequate, as intra-organisational channels of communication have been unreliable d) resulting in an incredibly slow process*

*a)*

"Certainly project management, as I’m accustomed to, requires you to engage the key players. And in the way that we brought a project team together I’m content that we did that fairly well."

*a)*

"[W]hat we could do and we did do is get a stakeholder group established and well represented and thoroughly engaged. That worked well with representation from across trusts and all sorts of people."

*a)*

"We’ve had excellent engagement in terms of the stakeholder meetings and so it’s really exciting to have that sort of arrangement in place where you’ve got such good representation of everybody to try and show that what you are delivering is appropriate."

*a)*

"[W]e’ve got a good group of people with different knowledges to be able to … make the best of the project … that’s been a strength."

*a)*

"[T]he strengths then, I think is the fact that there is a mixture of clinicians and non-clinicians on the team."

*a)*

"[T]here has been a dedicated resource around it in terms of support, IT, technical support, strong leadership – [4 names/roles], so there’s been a very strong group around it. And that means that whenever we have little hiccups or just extra communication, it’s all been fed in and the response has been quick."

*a)*

"[Part of my input has been] to clarify some of the more clinical issues, things like particularly the DNACPR, advance decisions, those types of things, when it’s very easy to get bogged down by Read Codes and all the rest of it, to keep us focused a bit more on what that means clinically and what is important to include. And it’s good having [name] with a clinical hat on as well, because he can look to me and I can look to him and we can sort of support each other and be a small voice amongst IT people!"

*a)*

"It’s good that there are, as I say, clinicians who feel passionately about getting it done and hopefully between us we manage to drag it back to the patient when we need to."

*b)*

"[W]e had an initial view, very ill-formed and, honestly, uninformed as to how it would work within [System D], partly because I don’t particularly know much about practice IT systems and don’t have particular interest or expertise in that area but, as I say, slightly naively going in being told by [name] and one or two other IT people that you can share data. As we subsequently discovered that was more challenging than we thought."

*b)*

"Without [names] we wouldn’t have made any progress, I don’t know how we ever thought we’d make any progress not having known who they were ... Talk about a leap into the dark, it was naivety in extreme and if only we’d been able to engage with those two people sooner!"

*c)*

"We had [names] who were the participants [in project team meetings]. I think it depends on how proactive they are in sharing the information as to whether you feel part, as if you’re participating. And maybe, I’m very keen on data collection and making sure that we’ve got robust data and was involved with setting up our [System D] data system, and tend to be seen a bit as a lead in it, and I’m very keen to disseminate information, and to make sure people fully understand what it is they’re getting into, and maybe we just didn’t have enough feedback from the members that were there to know."

*c)*

"We are using it because, as I said, we were really told we would have to use it and that was that, there wasn’t a discussion. Which is fine, but without really understanding what benefit it was going to give or how helpful it was going to be, so [left incomplete]. I think if you’re still having, if you are still having stakeholder meetings, it’s making sure that [the two people above] feed back to us. Are they still attending those meetings?"

*c)*

"So it may just be that [pause] maybe also communicating it to our [pause] making sure that the people that attend the meeting actually are attending the meeting and feeding the information back. And if someone can’t attend, that they ask someone else to attend, and that there’s enough notice to make sure that you do get to the meetings. Because if [name] couldn’t attend and she asked me if I could, then I would do that, or my other colleague, [name], who’s quite keen on this sort of thing ... [I]t’s making sure that somebody does go and then feeds it back to the team. It’s partly, it’s partly our problem if the team that are going haven’t fed it back, then it’s yeah, that’s not so much your problem. If you’re giving them the information, then they need to pass it back to us."

*d)*

"[T]he other frustration, inevitably with things like this, is how long things take to develop and how difficult it is reaching a consensus. Because if we look at the in-house template that I made when I was a Registrar, I produced that in two hours, and Share my Care has taken, what, a couple of years and a massive team of people. And they’re not the same, there are some differences, but the differences are pretty small, it does pretty much the same thing. But ... Share my Care obviously does it in a way that’s been agreed by multiple people and the palliative care teams are happy and all of the other practices are happy.

*d)*

And it just seems to [be] so difficult to do anything in the NHS in that you set on a project, you have to set up a board, you have to set up a stakeholder group, and ultimately after, if you think about one template that took two hours of one person’s time and Share my Care, which must be running into thousands of hours of man [?] time, when you look at all of the hours of the project team and all the stakeholder groups attending the meetings, and all of that kind of stuff, and you look at, 'okay, well, is it really a thousand times better?'. I wouldn’t, clearly, clearly it’s not."

*d)*

"[I]t’s just how long it takes to do all of this stuff. And Share my Care is clearly a good idea, clearly there was a capability to do it in [System D], fairly easily, but just trying to force the whole thing forward has been a real struggle.”

*d)*

"And that’s where the NHS [pause] falls down really, because if it was a private company running all of these, somebody senior in the company would say, 'Yeah, this is what we need to do, this is going to work', and it will just happen. And someone would design the template, it would be implemented, there’d probably be a bit of feedback and it would be fine-tuned, but it would be all done and dusted within a few weeks. Whereas it’s the fact that this is the NHS and this is the way that things work that it’s taken such a long time. And even so we’re still, we’re only part way through implementation of it now, and there’s still going to be some practices that are slow into it. So I know it’s a big organisational body, but I’m sure, [if] Tesco’s wanted to implement new till systems [both laugh], it wouldn’t be quite so difficult."

*Many of the people engaged in this project have shown exceptionally high levels of commitment and enthusiasm, often going beyond the formal requirements of their roles (a)*

*vs.*

*Most of the people engaged in this project have much broader roles, which limits how much they can do (b) or At times, the level of ownership tipped into possessiveness and territoriality (c) or Involvement has not always been appropriately funded (d)*

*a), d)*

"[W]e’ve had a huge amount of time and thought and effort and energy from people whose time was not funded by the budget at all, so the IT team and people like [names] … have given an enormous amount of time to the project, they’re continuing to give an enormous amount of time to the project, whereas they weren’t named in the list of co-applicants or the budget holders, so as the project developed, we discovered we needed their support and advice and they came on board."

*a)*

"[I] was more about the commitment and the involvement of the likes of [name] who really drove, and had an interest, and wanted to move this forward, had taken ownership of it and that’s the way it should really be, it shouldn’t be a project manager or IT person sort of leading on development of templates because it’s all about how that template is used to promote good patient care and sharing of that information."

*a)*

"But [name] was excellent. And [name] was excellent. When I say ‘excellent’, all of these people were putting a lot of effort, thought and time into how we should do things."

*a)*

"I’m very optimistic about the success of the Share my Care project … this is one of the few areas where you see so much enthusiasm from clinicians and everyone, I guess, involved in the process to try and get [it] right."

*b)*

"I’d love to be able to say, ‘yes, I’m going to do this full-time’ and then I could throw myself into it heart and soul but … it has to be a small part of the other three jobs that I’m doing so I can’t … be everywhere I would need to be or be as available as I perhaps could be."

*b)*

"I guess I envisaged initially that I would do this up to a point, until somebody else came along … and I could hand over the reins, but nobody’s appeared and I don’t know whether that’s because I’ve … kept my head above water … so, therefore, there hasn’t been the drive to find somebody else … or whether it’s just the way it is and everybody’s happy with the amount of input that I can give."

*b)*

"[S]ome things we have to do just because something’s happened and a service will lose their N3 connection by tomorrow if we don’t drop everything and we do something about it … [W]e have to be very good at responding to changing needs."

*c)*

"I and [name] reflected on a number of occasions a degree of discomfort that the project was being taken over by them and we held our tongue, didn’t challenge them and rode the wave that felt a little unsafe at times because … [name] and I were not setting the pace. But something was happening and things were being done and feedback was being given."

*c)*

"I wondered at one or two points whether I needed to say ‘actually, hang on [name], this is our project’. And that would have been potentially quite damaging … [I]t didn’t feel entirely comfortable, but we don’t live in a perfect world and we were getting a lot back from this relationship."

*c)*

"I didn’t feel there was a subversion, there was just very, very, very strong ownership, it was almost too much of a good thing … [A]s it turned out, that’s worked out fine and the individuals concerned have now completely reined back, but they were hugely committed … and giving up industrial amounts of time, and we learnt a huge amount from them. … [I]t had some tensions, it generated a huge amount of good stuff and wasn’t … deeply problematic, yeah."

*c)*

"[C]ertainly initially [name] was a real driving force and kept everything going … but it became almost too personal."

*d)*

"[T]he reason I’m fairly laidback about the fact that it hasn’t gone as fast as I’d have liked it to is I haven’t charged the days either. … [I]f the project manager had been recruited to this project on … the original bid was three days a week for a year and a bit, once that person had been appointed, they’d have been charging three days a week [irrespective of slow start and fluctuations of work] … then the number of days charged would … have been consumed at a very rapid rate."

*d)*

"But when someone is running a project completely based on goodwill … I have taken the view … it is not a good thing for me to be leaning on people and demanding of them to do it faster or quicker or … better, if what they’d done was not good enough … [N]ot when it’s not their main job."

*d)*

"There certainly have been times where … I had thought, if this had a person who wanted or was willing to push it, push, push, push, hard, hard, hard, bully, would that help? But my take on the situation we’re in is that … we’ve got a whole load of NHS employees and we rely on their contribution as a point of goodwill, all of them can, in essence, refuse to cooperate."

*Difficult issues have been discussed, and discussed openly, constructively and professionally (a) vs. Important negative views were not put on the table at team meetings (b)*

*a)*

"[I]t’s a token of the team that we’ve got, but when we’ve had feedback that hasn’t always been easy to hear, it’s been with colleagues who we know aren’t out to score points. … [W]e’ve talked about the difficulties that [organisation] have with their [staff], etc., etc., … [name]’s been surprisingly honest. And when you have flagged up with me that [name] team had anxieties … I was really worried about that meeting, wondering what on earth I was going to get, but … we talked very openly and that was very constructive."

*a)*

"[E]verybody working together to kind of talking things through. So when we’ve had the template … nearly there and then it’s been ‘well hang on, what about this’, everybody’s been very … professional, if you like, in talking the issues through and trying to come to a result."

*b)*

“[Name] perhaps picked up that this was for a particular project via [name] and said we could help with that, because [name] recommended more efficient ways of working with things and the templates and the ability to share it out. And there were some tensions at the beginning, I think, with [name] feeling undermined ... So we had to do quite a lot of bridge building, said 'we’re not trying to take it away from you or take over the work, but we may be best placed to support this'. And I think that was how it ended up that [name] was the main attendee. ... And there was, yeah, there was a lot of sensitivity at the beginning about whose, 'oh, I’ve done a lot of work on this', and it was, yeah, it was a little bit sensitive."

*b)*

"I had a bit of a dilemma about a fortnight ago because I was still using the old guidance notes and then [name] had said to me, ‘they’re not worth the paper they’re written on’."

"[W]hen I’ve looked at ours in relation to, say, the Coordinate My Care ones from London, ours are very … inconsequential really … their guidance notes are very, very comprehensive … almost to the extent that anybody could pick it up and work through and know what they were doing."

*b)*

"[O]ne of the difficulties that I’ve really struggled with, not just with this project but with the other projects as well, is getting engagement from [organisation] ... I went to see ... [name] to say, ‘can I come and see some of the [organisation] doctors and deliver the training … and I’ll come in as many evenings as you want to’ … But it wouldn’t have mattered if I’d offered to come in every single day and see them one at a time because her view was, ‘all of our doctors either know what it’s all about or they’re working as GPs anyway so they’ll pick it up from their GP practices’. And the most she was prepared to give me ... was a three or four line paragraph in the newsletter that goes round to the [organisation] staff … [T]hat, to me, says that they don’t appreciate the value of what we’re doing or the importance of what we’re doing."

*Perceptions of the MDT coordinator role as a) crucial for enabling data sharing vs. b) problematic*

*Both a) and b)*

"I very much hope … the MDT co-ordinators continue, because I think they’re what makes it happen particularly for the non-[System D] practices … I’ve done my little bit to augment that, but I’m not convinced that they’re going to be here for much longer. I don’t know, but the feeling out there is … ‘is this really a good use of three quarters of a million pounds?!’."

*Both a) and b)*

"[T]hey are only contracted for 12 months, so unless they … [get] referred on for another twelve months, it could fall … [because] the practice is so used to the MDT coordinators ensuring this work is done, that they then forget to do it themselves."

*a)*

"I can’t see it [end of life care data sharing] surviving in general practice without co-ordination. Having the MDT co-ordinators was a really big change, it really enabled things.” *[Palliative care professional 1]*

*a)*

"[T]here is a lot of interlinking between multidisciplinary working, bringing in the co-ordinators is also thoroughly linked because at the MDT meetings they will be discussing not only people on the frail older persons’ register, they’ll be discussing people on the End of Life register, so the training for the MDT co-ordinators has included Share my Care sessions, the training for the community matrons includes both."

*a)*

"[T]he game plan is developed by default that we’re going to try and drive it through MDT coordinators."

*b)*

"[T]he other issue which confounds all of this is the appointed MDT coordinators ... [T]here’s a huge amount of money been spent on them across the whole CCG, we’ve had one appointed to us, I haven’t met her yet ... [N]obody’s really sure what they’re supposed to be doing. I’ve got an idea of what I want our MDT coordinator to do, but I’m not sure whether my idea will fit with her idea of what the job is. ... [Because] this is a relatively small group of people, if you could train all these people to be very proficient at using them [data sharing templates], that would be a really good way of getting that into the practices. They’re our key group to focus on really, but there’s a lot of uncertainty about what their role’s going to be and how it’s going to work and whether they’re going to make a big difference.

*b)*

“It’s going to be difficult given that £750,000 has been spent on them. It’s going to be exceedingly difficult to demonstrate that degree of savings actually ... £750,000 is a huge amount to spend. I’m a little bit sceptical about whether we’ll ever see that degree of benefit from that project."

*Both a) and b)*

"[T]here’s two ways of [getting feedback on the template]. There’s a formal feedback loop to the official Primary Care Information Team and there’s the MDT coordinators, who are going to learn a lot. But the thing is the MDT coordinators are gradually becoming a solution for almost everything, which is rather unfair."

*Both a) and b)*

"[T]he MDT coordinators, they could bring more to this, but ours isn’t particularly proactive, two months and I still tell her what to do all the time. ... [T]he ability of MDT coordinators are different, because I’ve heard that the [LCG name] one is excellent and is doing all the templates for the doctors." *[GP 3]*

"[T]hey [new provider] guaranteed those 20 MDT Coordinator jobs, but ... perhaps in three years’ time they’ll have a hard look at it and actually [be] saying, 'this doesn’t work', and do something different. Which will be fine, but I’m optimistic that we won’t get any illogical, irrational, abrupt stopping of these work streams. I think we’ve put in place something that it’s going to have its natural life, and it will only be replaced when there is evidence that there’s something better. So I’m optimistic."

*Perceptions that a) local data sharing projects have served as catalysts for one another and also validated the overall concept vs. b) collaboration between local data sharing projects has been insufficient*

*a)*

"[P]eople had developed in-house templates to summarise information, to try to avoid admissions. We then perhaps slightly sort of sped past and overtook them, because we had dedicated project with time and other things, and they very much now with [name] and the MDT co-ordinators probably overtaken us or certainly ... not that it’s a race … [W]e had a big push and a leap and were able to move things on and the whole thing’s been catalytic really, but if it hadn’t been that environment, this might well not have happened."

*a)*

"[P]artly that’s the MDT co-ordinators and [name]’s project that just suddenly has made this so mainstream that we’re quite happy no longer to have a special name."

*a)*

"[I]t would be good to link up with [name]. It would be good to link up more ... across the clinical leads, because we’re all doing similar things. I must admit, I will say that I think I have been quite sensible and sat back and let people like [name] drive, I’m damned [?? 37:32] if I’m going to make the same effort, it would be the same thing, let him trailblaze and then we’ll follow.

*b)*

“But there needs to be a little bit more thought ... perhaps there needs to be a bit more linkage with the other clinical leads that fit into End of Life Care. ... I wouldn’t even say the lead, but the whole team, there needs to be a bit more cross over and communication at the CCG level about what the teams are doing and ... how we can work together even more and avoid, reduce our workload."

*Both a) and b)*

"We should help each other. And what that would do, which again I said, we need more of a clinical voice overall, so that will encourage colleagues to say, 'Okay, [name and name], blah, blah”, they’re all doing it'. So suddenly you’ve got more credibility. People think, 'well, these templates, okay, they’re a good thing' ... they wouldn’t be thinking, 'Uhmm, end of life' and then it’s, 'Oooh, there’s another one, there’s dementia'. It would be a joined-up approach. ... [T]here wouldn’t be constant little dribs of information, which annoys people, there will just be one ... [W]e all need to join, and I’m at fault there, a lot of it’s time, but it would save time if we did a bit more of that."

*Perceptions that the collaboration between the End of Life Care data sharing project and its closest partner project has been a) efficient and productive vs. b) often difficult*

*a)*

"By then also [name] was pushing them [IT team] and using them to develop the frail and elderly template. And that’s where [name] became an extremely useful ally, although his agenda was frail and elderly he was using resources and expertise that, in fact, we needed access to as well."

*a)*

"Likewise, having [name] there, that’s also helped drive a good relationship."

*a)*

"I was quite keen for [MDT] template and our template to be one and the same, [name] has always wanted them to be separate and there’s pros and cons on that. And we’ve gone with the flow and that’s probably right, certainly for where we are at the moment. But the MDT co-ordinators are in post … [with] at least some of them giving time to End of Life Care patients as well."

*Both a) and b), focus on a)*

"[W]e’ve worked very well together, the tensions are just sort of, sort of egos and personalities, mostly on my part, so I think we’ve aligned lots and lots of things. ... [We]’ve come up with some agreed terminology for special patient notes, and that’s a single signposting way for both frail elderly and End of Life, and we’ve included learning disability and other things in that, so that was a bit of work we did together with 111.

We’ve, I’ve been very happy to allow the MDT coordinators to incorporate all - the End of Life care template, the protocols and training, and to get them to think of it as a continuum as well, and similar population patients with slightly different needs. So we’ve aligned that pretty well. So I think just some of the things you may have noticed are just sort of some philosophical things, we’re all on a journey thinking about things in different ways, and eventually there’ll be a consensus."

*It has been possible to tap into broader highly attractive incentivisation schemes, such as PDMA (Practice Development and Management Agreement) and CQUIN (Commissioning for Quality and Innovation), not least by facilitating reporting for them (a)*

*vs.*

*Incentivisation has been much more limited than hoped for, even agreed (b)*

*a)*

"[A] number of the LCGs have introduced End of Life Care into their PDMAs or Local Enhanced Services … but if you’re going to get the money, which is quite a significant chunk of money, you can either trawl through all of the notes of your End of Life Care patients or you can use the template and the report is there."

*a)*

"The shape and the eventual solution to the PDMA that the CCG use to incentivise GP’s to use [the project], the eventual agreement there could well be quite significant, because it will be a matter of points means prizes and money into GP practices."

*a)*

"[Organisation] has a CQUIN this year, called CQUIN 6, and the target is about the proportion of people on the district nurse and community matron caseload who have a Share my Care template completed and, secondly, that it’s updated. And then the final element of the CQUIN is around the proportion of people who actually die in their stated preferred place of care … [T]here are mirror CQUINs for our partner organisations so, for example, [hospitals], etcetera ... [T]he CCG has tried to put in place the right environment to support the implementation of this, very much so."

*a)*

"MDT work has really taken off. It was partly sparked by that CQUIN, but also there was Admission Avoidance Enhanced Service, where we paid GPs to identify 0.7% of the adult population aged 18+, this was in 2013-14. We excluded End of Life from that, because we wouldn’t want them just to take all the End of Life Care register ... But from the 1st of April ’14, a new enhanced service for proactive care planning incentivises GPs to identify 2% of the adult population, and at that point we allowed them then to include all the End of Life Care. And we’ve so far identified something like 12,000 patients across the CCG."

*b)*

"I was disappointed that slightly under half of the practices have got End of Life Care in their PDMAs for the next financial year, because if you’ve got End of Life Care in your PDMA, then you’ve got to report how many of your patients who died had just-in-case drugs in place or had a DNACPR or died in their preferred place of care. Well, you either do that by looking through the notes tediously or you use the template and the Dashboard, and that’s the financial incentive."

*b)*

"[I]n terms of, maybe nationally, as I understand it, we’re not doing too badly on preferred place of death. So there’s not a lot, necessarily, we can do to move a lot of activity out of [hospital], for example.

There’s certainly ... problems around the ambulance trust and the 111 and inappropriate admissions, but we don’t necessarily have to do a massive drive and, therefore, it’s very difficult to attach any money to it except for greater cost, because good End of Life Care costs, generally speaking, more than you think."

*Persistent, multidirectional communication about the data sharing tools is needed*

*vs.*

*Recipients struggle to find the time to engage with the project communication work or related IT updates*

"[M]ore campaigning, more meetings, more publicising and you’ll get everybody using it. It just needs that push behind it, it needs that reminder, it needs to just sink in."

"[T]he more people we can have on the team going out there singing its virtues and pushing for it and just keep chipping away and chipping away, ultimately, we’ll get there."

"It’s kind of selling it, isn’t it, that’s what we need to do, is just get people to see the value of using it." *[GP 6]*

"[W]e have clinical governance meetings where all the GPs meet in [city] and share such things ... that’s where I found out about End of Life coordinator and about lead [for the] CCG. Otherwise we, GPs, work in our rooms, we don’t know what’s happening down the road in the next surgery to knowing about what’s happening overall in the city, and with things changing every 6 months in NHS, it’s difficult to keep [up to date] what End of Life data sharing we are doing, what template we are using and how we’re getting on ... So sharing knowledge is helpful and it should be done more." *[GP 5]*

"[W]e don’t have time to read through [bulletins], we don’t even go to these clinical governance meetings, so there should be some incentives to go there ... or we’re given time off or something. That’s the main problem, no GP has got time, we all want to learn, we all want to increase our knowledge, we all want to know about the local services. But getting some time off, getting some time for your [professional development], that is helpful, I don’t know how that’s possible with the circumstances in the NHS and the funding, but in an ideal world that’s what I would like." *[GP 5]*

*Positive self-awareness of the downsides of one’s approach or a narrative resolution of issues that should have been actively addressed?*

"My biggest worry, and this is a purely personal thing, is that the project would be better if there was a full-time education facilitator working just on this and I worry that I’m not able to give as much time as it needs and how much better would it be if there was somebody doing it full-time."

"[T]hey’ll regard me as bossy boots and a heavy weight in more than one way. And don’t want to be taken over, so you get antibodies back, and that’s all right, I entirely understand that. So it was just probably the, it was my fault for the way I tried to explain it. But I don’t think it, I don’t think it really matters now."

"[W]e’ve worked very well together, the tensions are just sort of, sort of egos and personalities, mostly on my part, so I think we’ve aligned lots and lots of things."

"I’d like to think and … it’s for others to judge, that I like working with other people, because I’m conscious that I don’t have all the answers. And the danger of being determined is that if you’re too determined, you can be pig-headed and not hear what other people are saying … But if you’re too meek and too collaborative, then maybe nothing ever gets done, so it’s a fine line. And in this project I know there are some things of which I’m completely ignorant … So hopefully it’s playing to other people’s strengths and hopefully making other people feel valued."

"[T]he only thing I feel I could have done more of is to make more noise when we weren’t getting what we wanted to make this project go forward, because, I think, that’s where the greatest weakness has been. We’re not able to get people to go at the pace we would like them to go."

"I think you’ll find almost everywhere else though the project manager of such a project would know a lot more about [System D] than I have."

"And maybe the project manager should have been someone like [name] who might, with a grasp of the detail around [System D] requirements and information governance and things like that … the theory of how to manage a project would have been better followed."

"This project is unique for me. It is the only project I’ve done where I’m not regarded as a subject expert."

*Conflicting perspectives with regard to the broader context*

*Strong positive attitude towards data sharing (a) vs. The value of data sharing is considered second-rate in comparison to more tangible sources of healthcare services improvement (e.g. funding, staff) (b)*

*a)*

"I'm ... just massively in favour of it [end of life care data sharing]. It’s something that is obvious, it’s evidence-based that in other regions of the country, is it the South West, there’s clear evidence of reduction in hospital admissions and other such benefits ... [I]t’s one of those few black-and-white things that needed to happen because without that level of communication so many mistakes will happen, or failings of care, perhaps I should say, where things just don’t go as smoothly as they could have done, so I’m 100% behind it."

"[T]he positives to me are overwhelmingly, the case is cast in iron, it’s a very strong case for this, but I’m mindful of the challenges and barriers of which inadequate communication, tick box communication is one of the biggest ones." *[Palliative care professional 3]*

"It saves a lot of time and frustration and all the rest of it, so as long as people read what’s there, then it’s hugely, hugely helpful, yeah, you can see [38:32] I’m a fan of it!"

"I hope the project continues and is embedded and ingrained, because it has been very beneficial and, again, it’s brought quite a lot of cohesion for people. So I think it’s good." *[Palliative care professional 5]*

" I want it to share, I want to know what the district nurses have done today ... because that’s very helpful when the patient is calling me to advise on the phone and I can see what they have done, because most of the time the patient doesn’t know what they’ve given them." *[GP 5]*

"I think the benefits need to be sung about." *[GP 6]*

"[T]hat’s why we’ve chosen to run with it, because we know, if we’re seeing those patients and we’re doing it, and then out-of-hours are able to see it, we feel we’ve got things covered a little bit more, and we can then help the GPs without them having to worry so much about it." *[Palliative care professional 2]*

"It is really useful and, providing it’s secure, I’m in favour of it." *[Palliative care professional 6]*

"[W]e should be sharing, we should be doing it appropriately”

"Information sharing is a really good, positive thing that protects patients rather than harms them."

"[M]y take-home message is, the more we share, the better it is for patients." *[Out of hours professional 4, GP]*

*b)*

"So if I would say to them, the Emergency Department, 'You are 100 square metres, I’m going to give you another 30 square metres, will you take that?', they’ll say, 'Oh, yes please, thank you. And you’ll cover the running costs and you’ll give us this extra staff?', 'Yeah, of course'. They’ll say, 'fine'.

If I say to them, 'You have 100 people aged 65 and over coming through your front door, and I could give you information that will help you to manage them', that’s too soft.

'Ah, information, that means I’ve got to read it, that’s extra work for me, I’ve got to read, I’ve got to have a system where I’ve got to extract it from another computer'. And it may be that 8 out of 10 times it won’t make any difference. 'So I’ve got to read 10 sets of records extra to get 2 patients where it’s actually helped, is that what you’re saying [interviewee name]?' [Both laugh]"

"[I]t feels like you’re trying to sell a second hand car, a broken second hand car."

*Transition is a) difficult vs. transition is b) for the better*

*a)*

"[T]he palliative care service in the hospital is actually used to using IT on a day-to-day basis. The irritation at the moment is that you write something in the paper notes and you’re trying to do a short summary on the palliative care IT system, so we’ll be really pleased when we’re only doing one [laughs] and not both. And then it will be easily visible for everybody else as well." *[Palliative care professional 1]*

*b)*

"[R]ather like [clinical IT system] being a necessity here, the data sharing project was something that just had to happen. And in a way these two big IT changes happening at the same time is very positive, because the way of working that each of them brings will help the other one ... they’ll be synergistic."

*a)*

"Yes, except, of course, they oppose each other at the moment, in the sense that people are just lacking the time and energy to be involved in a system which isn’t the primary one they’re using when the primary one is so challenging. But it really will improve." *[Palliative care professional 3]*

*a)*

"One of the greatest difficulties for the nurses is because they’re working in the community and patients’ homes with people who are housebound, they don’t have immediate access to IT at all, so at the moment we have a cumbersome system of their keeping paper records in the patients’ own home and then doing a limited amount of documentation on [System D] back at base. But there is that perpetual tension between the clinical demands plus the recording demands because, essentially, we’ve increased the amount of time it takes for them to record."

*Both a) and b)*

"The reason why that’s not happening [increasing access to the shared community data] as quickly as we’d initially envisaged is because of [clinical IT system] coming in, in that it’s such an overwhelmingly new and challenging system, which will be excellent, undoubtedly, but at the moment is proving quite problematic, and that now is not the time to increase the access to [System D]."

*Both a) and b)*

"[There is] an understanding that, fundamentally, there’s no way, no way back, this is the only way forward in that things are entirely inefficient in paper form, but probably a misunderstanding of how one can’t overnight change the entire way that everyone works. Even simple things, like when you go to see a patient, you bring the drug chart with you, because then, as the patient says things to you about what they’ve been experiencing, you can tie that in with things that have been happening pharmacologically and you can’t do that when it’s just on a computer on the ward.

So it’s simple, it gets to the very heart of how people work, and in a way that perhaps was beyond even what we’d imagined. But I’ve no doubt that over a shaky year people will develop new systems quickly. It will be, it’s a revolution really rather than an evolution, they’ll develop new systems, which will eventually make it work very well."

*a)*

"A consultant colleague of mine I met on Friday and he looked exhausted and he said, 'I’ve stayed in the hospital until after 10 o’clock every single day this week doing my own work and my secretary’s work', and there’s a growing sense of how big this challenge is. But as we were saying before, as with the EPaCCS, with the Data Sharing Project, it was obvious it needed to happen. It was equally obvious the transitions would be difficult, and so one just needs to keep plugging forward."

*a)*

"[M]y emails have just reduced massively since [clinical IT system] came in and emails I send are not responded to, because nobody has one spare bit of time or energy to deal with anything else, apart from the basic trying to keep patients safe, trying to work out where the referrals are coming, how are patients not getting lost in the system. So that has gone right down to the bottom of the pyramid. It’s survival, and adding in the luxury of being able to communicate well and see information in the community to avoid unnecessary conversations in hospital or build on existing conversations, or feed back to the community about conversations here, that feels a bit higher up."

*a)*

"[P]eople are very wisely trying not to become too emotive in the way they frame things, but one does hear some extremely catastrophic things being talked about. I personally think that it will be fine, it’s just there’s going to be an element of, as with all big revolutions, some people not keeping up with it, despairing before they can ride the course." *[Palliative care professional 1]*

*a)*

"[A]bout 18 months ago the out-of-hours service switched to [System D] and it was total mayhem to begin with, because there were problems with the appointment bookings and there were problems with the mobile computers, and they worked very very hard and eventually got all of that sorted."

*“Everything is IT”; “IT is the only way forward” (a) vs. Expectations of IT may be too high (b)*

*a)*

"[I]t’s worth, thinking about, it’s food for thought that we’re moving into an era now where everything is IT, everything is electronic clinical records, you’ve got Twitter and Facebook and YouTube and everything, all formats and places where we can get patients to input into things. [We've got] patient advice, patient apps, all sorts of ways of interacting with patients as part of the modern day culture and moving that way. And if we don’t move along with them, there is going to be ... a disconnect if you will, because things are still going to come out like electronic booking systems, information systems, integrated care, portals to view information.”

*a)*

“As a health professional, you [are] bombarded, bombarded, bombarded, we’re trying to make that into a single place, but there’s going to be a need, particularly with clinical led commissioning and service planning, that there is a level of understanding of the IM&T. And I don’t mean understanding what a secure socket layer [is] … but just understanding, being able to go through concepts of IT."

*a)*

"I really do mean this, I really think that this was the only way forward, this had to happen. It was just a question of how and when, not whether it should happen."

*a)*

"[There is] an understanding that fundamentally there’s no way, no way back, this is the only way forward in that things are entirely inefficient in paper form, but probably a misunderstanding of how one can’t overnight change the entire way that everyone works.

*a)*

"It would be fair to say we’re are finding it challenging, but things can’t go backwards because it’s not coming from a good position and it will just be a challenge that people, I’m sure, will rise to because there’s no choice." *[Palliative care professional 3]*

*b)*

“I do sometimes feel that the template has become very [sighs] IT-orientated and … that the IT side of things means that we lose sight of the patient because the codes won’t allow us or the system won’t allow us and … it becomes, I’m sorry, but yet another piece of IT work, and we know how successful IT work is in the health service."

*b)*

"Sometimes people in the Trust get ... a bit muddled. There’s multiple systems within the Trust at the moment, which is part of the problem, many of them are different aspects of the electronic patient record, so the lab system, the x-ray systems, the letter system are all separate but they will be pulled together. But then there are other things like information about departments and patient leaflets that are on the intranet and they won’t be included in the electronic patient record, they will sit alongside it as they do now."

*Patient data sharing is in sync with everything being less “boundaried” and more accessible in everyday life (a) vs. The world is moving towards things becoming more closed off (e.g. with our obsession with data protection) (b)*

*a)*

"I’m not worried about that side of things [issues around consent] because in the big picture life is changing for everyone. People can look themselves up on Google Maps, can look into their garden, everything is less boundaried than it was. One never asks a patient, “Could I speak with your GP?”, even though we should, because there seems to be an implied consent and an assumption on the part of the patients that we would do that, we would speak with the GP. And so I think things will work out, patients will become less and less concerned by it, and just pleased that it means that their care can be joined up." *[Palliative care professional 3]*

*b)*

"It’s very difficult, isn’t it, because we’re so obsessed now with data protection that it gets in the way of sharing. And I don’t think there’s an easy fix to that, because I think the world is generally working towards closing rather than sharing."

*a)*

"[T]here’s things you share consensually ... and then there’s the sharing you don’t know about, i.e. the CCTVs and whatever is going around in the global world over viewing you, but that’s a long discussion! [Laughs]"

*a)*

"[W]hen you think about how much data is collected about you which you’re not aware of and shared about in random conversations, nobody’s ever going to ask you about that."

*a)*

"I just really struggle with the thought that Tesco’s can tell me how often I buy bananas, yet we can’t work out a way of holding information to make somebody’s End of Life Care better."

*a)*

"[A]nybody and everybody has a Tesco’s Clubcard that holds vast amounts of information and you just stick it in a machine and it pings up, your Boots Clubcard or whatever … why can’t we do that for healthcare, why is there all this hoo-hah about, ooh data protection and keeping this information and having access to patient records when we shouldn’t be putting anything on there that the patient doesn’t want us to anyway. Maybe."

*a)*

"He was perhaps trying to do a bit of devil’s advocate stuff, but his blog yesterday was talking about how many of us have a Nectar Card or a Tesco’s Clubcard, which provides big companies with all the information, incredibly identifiable about us ... so they can tell our buying habits, but we’re not willing to let the NHS have information that might inform healthcare provision. We can inform what goes on a supermarket shelf, but we’re a bit funny about what healthcare provision we might need to make available. I thought that was quite good."

*Some health professionals enjoy ‘playing’ with clinical IT systems (a) vs. Many health professionals consider computers and paperwork the frustrating part of healthcare (b)*

*a)*

"[T]he more you use these things [clinical IT systems], the more you get to enjoy them and to realise how useful they are." *[Palliative care professional 5]*

*b)*

"[W]orking with the patients is the most rewarding, obviously, that’s what we tend to do. I find having to do my administration and paperwork the least rewarding, because sometimes you feel that you’re having to double up on things and repeat yourself." *[Palliative care professional 2]*

*b)*

"I looked after a gentleman who’d been desperately uncomfortable for many, many weeks and ... was disappointed when he was told he’d probably got months left, because he believed he was going to be uncomfortable for months. And I assessed him in-depth with his wife present and spoke to some colleagues ... from the hospital, and we looked at his scans and things, and went back to him and said, 'Look, this may or may not help, but what I would suggest [left incomplete].

And he rang me within a few days and said, 'My life is transformed, I can’t believe it, I’ll be able to go to my daughter’s wedding, I’ll be able to do all these things!'. And you think, 'Gosh, to be able to make that happen at such an important time of life!'.

So it’s hugely rewarding, just those sorts of things would happen when you’ve been having a particularly awful morning with either computers or paperwork or management or something, you’d suddenly get a phone call like that and you just think, 'Wow'." *[Palliative care professional 5]*

**3.5. Ambivalent forces, unintended consequences** (“Dark side of the moon”)

*Out of date information may be more dangerous than no information*

"[Data sharing] is extremely difficult, and it’s extremely difficult because we work with [System A] and not with [System D], because most of the data sharing in this area is designed around [System D]. ... [W]e’re printing them [care plans for vulnerable people] out and then they’re being uploaded as a document on [System D]. But as soon as you’ve done that, they’re out-of-date. ... [T]he next day the patient’s medication might be changed, but that’s not synchronising. So, there’s actually risks with data sharing ... very much from the practice that I work in."

"[A]t the last meeting it was planned, this [updating of care plans for vulnerable people] was going to be done quarterly, but there are major issues with that ... in the sense that these are people whose medication and medical history, etc. is changing frequently and we would obviously update medication on the system and we would obviously update things, but that care plan will remain the same on [System D] until the next quarter when they’re all printed and they’re all uploaded again. ... [S]ome of the more vulnerable adults, you might see them in [out of hours service] two months’ later, and they might have had a side-effect from the medication since it was logged or something’s been increased, and that won’t be reflected on the care plan."

"[T]he whole thing just seems really clunky and potentially more dangerous than not doing anything at all, where at least when the urgent care clinician who’s never seen them just doesn’t know anything, and can go by what medication’s in the house."

"[T]here’s a danger that out-of-date information is more dangerous than no information." *[GP 4]*

*Easier to obtain specialist advice – easier to feel one’s work is interfered with*

“I can remember a couple of times picking up the phone to a GP and saying, 'I’ve had a look at [System D]'. You don’t want to imply that they’re not doing their best or it’s mismanagement in any way, but reading from what I can see, it does sound as if this patient’s in obstruction and probably not absorbing, the quickest way to get on top of the situation would be to have a syringe driver for a few days. Then quite often you get a GP say, 'Oh, they’re not at that stage yet, they don’t need a syringe driver' and you’d say, 'No, this is the symptom control for it, then it can come down again'.”

"So even at that level of just seeing what they were anticipating because why would a GP know? We have such a depth of knowledge about the pathway of the individual cancers and things that they won’t have because they’ll see one a year or one every three years or they’ve never seen Motor Neurone Disease before or even something like that and it saddened me that they didn’t ask us but again, you know, it was a good way of picking up on things. Whether it was a patient, family or a specialist nurse or even a district nurse ringing up and saying, 'It’s all going wrong'. You could look on [System D] and see what was happening and really didn’t think, 'Right, actually I’ve got an interfering person here, I’ve got, and sometimes GPs were not very pleased with us for interfering.' *[Palliative Care Professional 5]*

“So rather than getting uptight about it, let’s see what actually happened and, again, if it wasn’t clear on [System D], if it all seemed a bit odd, then again I wasn’t shy of ringing up and saying, 'Oh, I just had a look at [System D], wasn’t quite clear why such and such had happened' and ask them if we could work on it together, if there was a reason. Mostly it could be amicable, sometimes it took two or three exchanges with difficulties and a couple of GPs, probably the first question I ever said to the Macmillan nurses when they presented a patient was, 'Who’s the GP?'. There were a couple of names, early on, [Exasperated] 'Right'.”

“But that’s the challenges of working with people and you have to work with what you’ve got and again, you are the patient’s advocate in that situation, you’ve got to be prepared, and if the GP gets cross, [47:12] with, well with us is better than they get cross with the patient or the family.” *[Palliative Care Professional 5]*

*More support for those who know less – more pressure to take decisions one is uncomfortable with/ greater likelihood to be overconfident in one’s ability to make decisions*

"Anticipatory prescribing ... seemed to be something that made a big difference, and the main barrier for GPs was that most GPs weren’t doing it very often and doing an anticipatory prescription ended up being quite a lengthy process. A lot of them had to look up the strengths and the doses that they’d be giving and that would involve going back to the BNF (British National Formulary) or going back to some guidance, looking on a website, and manually typing it in into the computer and making sure they type the doses right. And ... for about 90% of patients it’s absolutely standard what you’re giving."

"[T]hat prescribing tab was really popular ... because it meant that they [GPs] could, this job that would have taken them ages before, they could do in about ten seconds just by clicking on the tabs."

"Again, this prescribing End of Life [care medication tab] is really good, especially those yellow charts and everything, but new GPs will find it hard because although we all know it, the first time prescribing we all make mistakes [laughs], so the simpler it can be, the easier it will be" *[GP 5]*

"[I]f you’re comfortable with prescribing, it’s easy to do, but if you’re not comfortable with prescribing Morphine, Oxycodone, Fentanyl patches, whatever, or syringe drivers, people find it frightening, and they then tend to not have the confidence to do it and they need to ask people like us about it."

"And they [the "difficult practices"] take their time because they’re not sure, or they ask you lots of questions about it, they’re the ones I have to be very proactive with and go in and see and talk to about, and that’s the safest way to do it then. Whereas if they’re all just expected to do it on the template themselves [pause], I’d worry that they wouldn’t be confident at it, and that’s why I wanted the pilot."

"[I]t’s more about being sure that the clinician is making the right decisions in the best interests of the patient, whether they feel comfortable enough with the medicines that they’re prescribing, that they are making the right choices, not just following a template. Just because the guidance is there doesn’t mean to say that that’s the best thing for the patient, does it?

So that’s a guide there, but you also need to think, ‘Oh, actually no, that patient’s already had Levomepromazine for their nausea and Metoclopramide. Oh, what else can I think about, what else should I be using? Oh, that patient’s got poor renal function, they’re very frail and elderly, they’re getting confused, maybe Diamorphine isn’t the right thing to be using first of all.’

I don’t want to be critical or think that the doctors don’t know what they’re doing, but some aren’t as used to using it as others ... [T]hey need extra support, it shouldn’t just be about ticking a box and filling in a bit of paper, it needs thinking about."

"They’re generalists and we’re specialists, so if you’re a generalist, why would you, every day you’re not going to be working with these medicines unless you have an interest and unless you choose, or if you happen to have a caseload that’s got quite a number of very poorly people in. I’m sure they’re very knowledgeable and very capable, but they need the support of colleagues like myself and the district nurses to be sure that we’re all making sure the patients have the right medicines." *[Palliative care professional 2]*

*Informing colleagues that you have started something – ‘permission’ for them to do less*

"I will write to the GP and say that I’ve done it, but the GP needs to be having those conversations and getting in to see the patient, not saying, ‘oh, well that’s been done, I needn’t bother to go!’ ... Personally, I think there are issues about allowing the GPs to do their good job and not trying to do it all in the hospital ... I’m not at all convinced that us inputting information is absolutely the right thing to do, but we do need to make sure that information gets to the GP and is acted on. So if I input information, say I started the template, say the patient hadn’t got a template at all and I started it, new diagnosis and they need this template, I’ve done it all, how do I know the GP does anything with it?"

"[The GP can think], 'this has been done, so I don’t need to do anything now, I’ll think about it in a month or whatever'." *[Palliative care professional 1]*

"[I]f we’re doing the template and nobody else is seeing it, or nobody else is updating it, or nobody else is amending it, it feels like it only works for us, it doesn’t give other people responsibility for managing that patient, and everybody should be responsible for managing that patient. And by not ticking the boxes that are very linked with the surgery, the red, amber, green bit, to me it felt like that at least gives me, 'well, you need to make that judgement, you as the doctor need to make that judgement', which is why I was a little bit less keen to tick those boxes." *[Palliative care professional 2]*

*Access to a well-established clinical history may prevent the fresh perspective where the solution resides*

“But then just by nature, just by how human nature [is], that’s how we sometimes think, aren’t we, we look back and think, ‘Oh, you’ve already phoned up about this or you’ve already had lots of problems”, and … that might change your opinion or change your thoughts about a certain patient, rightly or wrongly.’ *[Out of hours professional 5, Nurse]*

**3.7. Ambivalent forces, reversals of the negative** (“From defect to effect”, “Rainbow after the rain”)

*Learning from negative experiences*

“I have had a few patients who’ve declined to share notes, including a lady who certainly had a very nasty tumour.

And it was really interesting with her, because the first weekend I spoke to her, she said, I know her actually, she said, 'I don’t want you to have access to my notes and I don’t want my GP to know that I’ve been in touch with you'. 'Fair enough, okay'.

The following weekend, she said, 'I went to my GP during the week and she hadn’t got any of the information that we’d discussed'. And I said, 'Well, do you remember, that was because you declined to share information?'. She said, 'Yes'. I said, 'Well, can you now see that, actually, that disadvantages you?' [laughs]. And she was like, 'Oh, yeah, I understand now!'.” *[Out of hours professional 4, GP]*

*Trying as reversing strong negative attitudes*

"[I]nitially people found that a huge burden and then they actually found that having those conversations was helpful ... once they’ve put a toe in the water and discussed where does somebody want to die.

The best ones are the patients who surprise you with the answers. You think you know the answer and you’re almost doing it as a matter-of-fact thing and say, 'From everything you’ve told me, I’m sure you’re going to tell me what you want is to die here' ... and they say, 'Oh, oh, no, that would be the worst thing. I couldn’t possibly contemplate dying here!'."

"I made that assumption from what I’ve heard and I was completely wrong, it’s a brilliant learning experience. Hopefully, a few of our GP colleagues or district nurses will put their toe in the water, perhaps they’ll say it because they ... thought they knew the answer, and then suddenly found the answer’s completely different. And then you think, 'Well, thank goodness I did ask, because I wouldn’t have been planning in my own mind how I’m going to manage this situation'."

"[H]opefully, if our GP or district nurse colleagues have had that experience of a surprise answer, then they’ve realised how incredibly helpful it is." *[Palliative care professional 5]*

*Using potential weakness of others to one’s advantage*

"GPs are a funny bunch [laughs] in that ... initially they might say, ‘no, no, we’re not interested, no, we can’t do that’, but when push comes to shove, they are hugely competitive and they don’t like to think that the practice down the road is doing something that they’re not or getting something that they’re not … [Y]ou only need one to say, ‘oh yes, we’d like you to come to us’ and then the next one will say, ‘why don’t you come to us as well, we haven’t seen you yet’ and they suddenly start … this knock-on, domino effect."

"[I]f you look at the feedback form ... we had so far from the GP practices around the different objectives, targets, and so they can compare themselves and uuuuh, that really spurs them on! Because they get all competitive and all, 'we can’t be seen to be the lowest one in this category!'. So that’s very good and that always helps, again, because people like to compare and you don’t want to be the last one in the bunch."

**3.8. Ambivalent forces, conflicting or unclear evidence** (“Unknown quantities”)

"I'm ... just massively in favour of it [end of life care data sharing]. It’s something that is obvious, it’s evidence-based that in other regions of the country, is it the South West, there’s clear evidence of reduction in hospital admissions and other such benefits ... [I]t’s one of those few black-and-white things that needed to happen because without that level of communication so many mistakes will happen, or failings of care, perhaps I should say, where things just don’t go as smoothly as they could have done, so I’m 100% behind it."

*vs.*

"I’m not aware of anywhere else in the country that has necessarily done hugely better than us. The [project name], when you go to one of their presentations, you can be left thinking 'this is hugely successful', but … someone commented that they’ve got less than 1% of their population on their EPaCCS system."

"[I]t’s difficult to say that it really is absolutely trailblazing ... And we just don’t know enough about what’s going on anywhere else."

**3.9. Ambivalent forces, vicarious learning or benefiting from the failures of others** (“Digital vampires or digital sages”)

*The End of Life Care data sharing project has (unintentionally) benefited from some of the negative attitudes towards another data sharing project, by having the latter as a contrasting background (a)*

*vs.*

*The negative associations with other data sharing projects could potentially harm progress for the End of Life Care data sharing project (b)*

*a)*

"[T]hey’ve all been very, very positive, they all really like it, and some GPs are more enthusiastic about it than others because End of Life is something they’re interested in ... I haven’t heard anybody saying anything negative, whereas with the other one, [project name], there’s been very, quite a uniform criticism of the template."

*a)*

"I found it really easy, very straightforward, I haven’t had a problem with the End of Life template. As I said, the [project name], yes, but, no [with the end of life one], the only issue was around the prescribing."

*a)*

"[B]y and large, people think it’s quite a good template, certainly if you present it next to the [project name] template, everyone looks at Share my Care [original name of End of Life Care project] and looks at the [project name] one and thinks Share my Care is brilliant because, you know, the contrasting effect."

*b)*

"I can understand why the likes of [name] … want to tie it [end of life care template] in with [project name] template … but that’s already a sort of an unmanageable template for a lot of people, so we don’t want to be saddled with that as well or we don’t want to be having the reputation of that going before our project."
