# Supplementary material for: From “wading through treacle” to “making haste slowly”: A comprehensive yet parsimonious model of drivers and challenges to implementing patient data sharing projects based on an EPaCCS evaluation and four pre-existing literature reviews
Source: PLOS Digit Health. 2024 Apr 1;3(4):e0000470. doi: 10.1371/journal.pdig.0000470 (PMC10984410; doi:10.1371/journal.pdig.0000470)
Supplement: S3 File — (DOCX) [file pdig.0000470.s003.docx]

**S3 File: Recommendations for action**

**Table 1: “Pure challenges”, “pure drivers”, “oppositional and/or ambivalent forces” and proposed actions to address them**

| 1. PURE CHALLENGES  Descriptive name of type or subtype  (Suggested mnemonic/ metaphor)^[[1]](#footnote-1)^ | Proposed actions by health IT actors^[[2]](#footnote-2)^  (partly derived from the data, partly involving new hypotheses) |
| --- | --- |
| 1. 1. Radical innovation challenges  (“I saved the world today”^[[3]](#footnote-3)^)  Key features   - system-wide innovation which needs to filter through to every “unit” within the system; - significant behaviour change involved; - life-changing impact as a result of changes to material structures and behaviours.   Parallels outside of health IT:   - development of large-scale recycling infrastructure; - water and sanitation systems in the developing world; - (historical) the arrival of the railways, the construction of the modern road infrastructure of a country, the growth of the aviation industry, etc. | |
| 1.1.1. Magnitude of repetition  (“Banality of evil”^[[4]](#footnote-4)^)  Challenges arising by the sheer number of entities for which the underpinning infrastructure needs to be set up (settings, organisations, teams, computers) and individuals whose behaviours and mentality need to be modified. | Map as comprehensively as possible the “entities” that need to be reached:   - This is a significant task. Consider, for instance, that an inclusive, but not exhaustive, count of the settings and teams that needed the tools of the EoLC data sharing project was 330. - This is a recurring task – organisational structures are dynamic; contact persons also change. - Freedom of Information (FOI) requests from the local Clinical Commissioning Group can help.   Create processes and structures for engaging with each entity and for monitoring progress.  Take into account that some of this repetitive work requires highly skilled communication and negotiation at top hierarchical levels (e.g. for securing buy-in); some is low-level technical (e.g. setting up computer systems); and some is in between (e.g. refining the tools).  Repeat. Repeat. Repeat. Repeat. Repeat. (Continue.)  Present or require data on the entities engaged as a proportion of all entities that need to be engaged, not only as raw numbers. The latter can be hugely impressive and the work done needs to be acknowledged, but the former (proportions) are the meaningful indicator of coverage.  Similarly, present or require data on different levels of engagement (having access to the tools is different to using them). |
| 1.1.2. Alternatives  (“1001 nITes”)  The range of alternative tools and workflows available or under development. | Map as comprehensively as possible pre-existing processes as well as “the competition”. Consider not only data sharing tools, but also structured forms for capturing information that may be quite similar but not have the sharing functionality.  Do not underestimate their numbers, value or capacity to persist, not least through a biased perception of the advantages of own tool. Consider, for example, that the data sharing project under investigation had a minimum of 13 *type*s of alternatives.  Consider which alternative tools can become partners rather than competitors.  At the level of the local (and national) health economy, decisions on support for some tools and processes to the exclusion of others, including through dis-investment, as well as standardisation initiatives, are likely to be needed.  (UK) Electronic Palliative Care Coordination Systems may be far more viable as an aspect of the Local Health and Care Records than as one of dozens, even hundreds, of data sharing tools. |
| 1.1.3. Vicious circles  (Catch 22 challenges)  Paradoxical situations, irresolvable dilemmas, vicious circles or “chicken-and-egg” conundrums, including:   1. Users who provide the shared data need to see – quickly – positive action from users who receive the data. Users who receive the data need to be able to find – quickly and easily – high quality data for the majority of patients for whom they need shared data. None of that happens. 2. Users need to like a system immediately to persist with using it. Developers need initial grace so as to improve it. 3. Innovation has a hard task against systems that are old and far inferior but “not broken”, deeply familiar and smoothly integrated into workflows. 4. Time, cost-savings and improvements of efficiency in the long term often require significant investment of time, money and loss of efficiency in the short- to medium-term. 5. (Relative) success could lead to disinvestment, especially when success leads to further financial demands. Doing good work with public money can end up becoming a reason for losing support. 6. Innovation requires a clear vision to move towards but is also irreducibly emergent. | (Actions in response to 1 and 2 opposite)  Communicate initial challenges. Involve initial users as co-developers, not as consumers who have to be quickly satisfied. “Initial users” may be users over the first few years.  (Actions in response to 1 and 2 opposite)  Do not scale up too early. Giving up on a system because of teething problems can happen very quickly.  (Actions in response to 1, 2 and 3 opposite)  Both organisational mandates and inescapable need (such as digital consultations during the Coronavirus pandemic) can extend the sensitive grace period. They can also offer windows of opportunity against the old, inferior but “not broken” systems.  (Actions in response to 4 opposite)  Resource. Communicate. Choose the right time to start implementation. NB: Do not underestimate the “resource” element above. This is not work to be done on ideals of better care and future improvements alone.  (Actions in response to 5 opposite)  This is a brutal paradox. In the case of EPaCCS, be aware that the better they function, the greater unmet need in palliative and end of life care they will reveal.  (Actions in response to 6 opposite)  Create processes and structures that are both reliable, able to maintain direction and place, and highly flexible, capable of responding adaptively and grasping new opportunities. Be aware that team members can have working styles that are a natural fit to one of those polarities (as associated with clear vision and structure vs. flexibility and allowing emergence). By extension, this will mean that their working style is in conflict with the other polarity. |
| 1.1.4. Uneven involvement  (“In the deep waters – toes in the water – high and dry”)  Settings are unlikely to be engaged with a comparable level of intensity. Even the most inclusive stakeholder group cannot include representatives from all relevant teams (if it is a “radical innovation project” as understood here).  A project team is deeply involved with some settings; has on and off communication with others; and still other settings and teams are practically excluded. | Even if involvement needs to be staggered, do not lose sight of the organisations left “for later stages”. Months, easily turning into years, of work excluding such organisations can result in processes and structures that are no longer suitable for their involvement. If they were crucial to the true project success, the project will gradually die away.  Establish processes and structures for collecting reliable feedback and data across relevant settings.  Be aware that a representative on a stakeholder group may not be the perfect communication channel. E.g. they may not have sufficient power within their organisation to mandate agreed actions or may not be trusted enough by frontline staff and not receive relevant (negative) feedback from them.  Be aware that actions confidently agreed with teams that are involved in an on-and-off manner may not be followed up on. Do not assume something will be done because of a firm positive agreement and actions taken up by competent, committed, responsible and senior (and/or nice!) people. Create processes for following up. |
| 1.1.5. Overvaluing the uniqueness of one’s project or idea (“Lovely baby problem”)^[[5]](#footnote-5)^ | You must, of course, have deep confidence in the value of your project (otherwise it is not yours to advance), but remember that the flipside of this is a tendency to overvalue its positives and lose sight of its negatives. |
| 1.1.6. Securing a host/ sponsoring organisation  (“Finding a home”) | Expect difficulties in finding a host or a sponsor, even if a project can be transformative, life-changing for a sector. Remember that you are acting out of the “lovely baby” perspective above (which may be completely justified, but an idea in its early years still needs time to reveal its potential), while potential hosts and sponsors are acutely aware of the risks, too. |
| 1.1.7. Impact of delayed external innovation  (“Innovation domino”) | Have alternatives if you have factored in the outcomes of other teams’ innovations into the enhancement of one’s own innovation. |
| 1.1.8. Slipping timelines  (“We are scheduled to finish 6 years ago”) | This is a well-known challenge in radical innovation projects. It seems practically unavoidable, no matter how hard a team works and how superior its planning, processes and structures. It is in the nature of such projects to be influenced by a huge variety of external factors outside of an innovation team’s control.  Mental preparation for the drag and practical back-up plans for the times when funding will dry up are key. But a drawn-out process and numerous times of hanging on a financial thread are most likely a given. Leave early if you are a short-distance rather than a marathon runner. |
|  |  |
| 1.2. Challenges of working in the health service  (“Living on the Edge”)  Key features   - challenges experienced by a healthcare system are replicated in any (?) project that involves it, not only health IT; - challenges experienced by a healthcare system limit the capacity of organisations, teams and individual health professionals to engage in innovation, even if that innovation aims to resolve them.   Parallels outside of health IT: possibly the sectors of local government and national bureaucracies are some of the closest parallels. | |
| 1.2.1. Resource limitations, such as:   - financial strain - staff shortages - high staff turnover in some settings - disproportionate impact of staff shortages on remote areas - stretching of roles - the competition of priorities for attention, money and staff time. | While data sharing projects argue they can resolve some (many) of the challenges opposite, they will exacerbate them in the short and medium term (see also section on Catch 22 challenges above). Under-resourcing such projects commits them to failure in the context of severe resource limitations in the health service. |
| 1.2.2. Fragmentation, created by and feeding back into   - significant levels of autonomy of organisations - lack of clear mechanisms for driving multi-setting projects - multiplicity of simultaneously running and often overlapping projects - lack of sufficient central/ national leadership. | Be aware that in many localities (at least in England), a large number of organisations will need to be engaged in a data sharing project one by one rather than through a reliable local mechanism for implementing health IT projects.  The need to develop pathways and processes for a data sharing project may reveal inefficiencies and irrationality at the interface between organisations. Remember that these are likely to be associated with well entrenched practices, valued services and people’s jobs. Attempts at streamlining may be faced with significant resistances.  At the level of a local health economy, it is crucial to develop and maintain effective forums that bring together digital health leads and other health IT staff from across organisations, representing a broad cross-section of roles at different levels and different types of expertise.  Map comprehensively local health IT projects underway. As in Alternatives (“1001 nITes”): decisions on support for some tools and processes to the exclusion of others, including through disinvestment, as well as standardisation initiatives, are likely to be needed. |
| 1.2.3. Constant transformation | Be aware than any new project will typically be facing significant accumulated transformation, project and innovation fatigue.  Making things better for patients and staff is a weak or short-lived argument in favour of a data sharing project. This is the argument for scores of, even hundreds of, projects and transformation initiatives, a significant proportion of which, perhaps even the majority, do not achieve their promise. |
| 1.2.4. Environment deprioritising ‘extras’   - relentless, overwhelming pressures - minimal capacity for taking up new work - at times low morale of staff - focus on “core business” - a culture of responding, firefighting and urgency rather than long-term planning - short-termism - crude decision-making models over-determined by a concern for saving money (now) - high levels of attrition and replacement of representatives from different organisations. | Be prepared for a long-haul game and potentially slow progress with overwhelmed organisations.  Prioritise the generation of evidence tracking a project’s impact over an extended period of time.  (For organisations and commissioners) Create more opportunities for long-term investment and evaluation cycles, as data sharing projects are unlikely to demonstrate fast return on investment. They require a significant refinement period after the initial tools and processes have been put in place.  Create processes for “induction” into a data sharing project for new representatives from local healthcare organisations. |
|  |  |
| 1.3. Work in large impermanent teams  (“Motley crew”)  Key features   - an ambitious common goal leads to the creation of new teams or organisations; - these are held together by a loose hierarchy and limited shared lines of reporting and accountability; - the primary belonging of team members is outside of the team/ organisation that works towards the common goal.   Parallels outside of health IT: supranational, multilateral organisations, such as the WHO, UN or the European Union. | |
| 1.3.1. Importance of fairness – in contribution and benefits | Each organisation and team should feel that both the work and the benefits are fairly distributed, although some organisations, teams and team members (e.g. specialist palliative care teams, GPs in the case of EoLC data sharing) are more willing to take up the bulk of the work for the benefit of all, with a focus on what is best for patients. It is still a fine balance that should not be undermined. |
| 1.3.2. “Politics” and conflict | Strong negotiation and facilitation skills required as a key resource for any data sharing project. |
| 1.3.3. Variety of languages, with particular difficulties around “IT speak” | Ground rules may need to be established early around explaining concepts and processes until everybody understands, at least up to a working level.  Communication skills and patience to explain of IT staff involved are paramount.  Honesty on the part of stakeholders, some of whom occupy very senior roles and are not used to be in the vulnerable position of the ignorant, is also key. Modelling of “I don’t understand” by senior staff can help significantly. |
| 1.3.4. Lack of action on agreed decisions and lack of reliable enforcement mechanisms | A memorandum of understanding and similar formal, even if not legally binding, documents may be needed to establish a basic framework for the work to follow.  Additional resourcing of data sharing projects is also likely to be a key mechanism for ensuring work committed to has been completed. |
|  |  |
| 1.4. IT work, narrowly construed  (“We’ve known you for so long”) | |
| Familiar health IT challenges, such as:   - multiplicity of non-interoperable clinical IT systems - Information Governance - mobile working - old hardware and software - key settings lagging behind with the implementation of comprehensive IT solutions - lack of basic IT infrastructure in some settings - pockets where paper notes persist - knowledge and skills: generic IT skills and record sharing-specific skills - high level of unpredictability of risks – resulting from the dynamics, complexity and unevenness around data sharing - IT projects are work in progress and may ultimately fail. | Standard recommendations for advancing health IT (e.g. work around interoperability, Information Governance, improvements in mobile working, training, etc.) confirmed as valid.  Data sharing is highly dependent on within-organisational improvements of their basic IT infrastructure. Much of its progress is almost entirely dependent on such improvements. Make sure, however, that data sharing across the local health economy is a key consideration in making IT decisions within individual organisations.  Map comprehensively local clinical IT systems.  Explore practices of applying IG rules. Organisational interpretations may be far stricter than the legal framework around data sharing demands. |
|  |  |
| 1.5. Rules, laws and algorithms in spaces also calling for humanity, flexibility and art  (“How to Repair a Broken Human. A Manual for IT Systems”)  Key features:  Contrast between   - the “orderly nature” of IT – of rules, algorithms, logic, rationality, 1s and 0s, clear boundaries, fixed choices that open up pre-determined pathways, etc. - the “messy nature” of healthcare – characterised by complexity, uncertainty, suffering, embodiment, humanity, intuition, creativity, “the art of medicine” in addition to medical science, etc.   Parallels outside of health IT: the application of laws, governance rules, bureaucratic processes, etc. into messier areas of life or its grey zones. | |
| 1.5.1. Mismatch between the IT representation and the clinical or practical reality  Examples: screens popping up in the clinical encounter that do not match its flow; codes in the clinical systems that do not represent accurately or fully all options; linear representations where there is significant iterativity and complexity. | Create easily accessible and reliable routes for feedback by users. Hold in acute awareness (without over-normalising) and communicate that the representation will never be the field. There will always be significant limits to the approximation of reality which IT tools aim to achieve. |
| 1.5.2. Use of unsafe workarounds | Elicit this type of “messiness” of the work as a priority. Create easily accessible and reliable routes for feedback by users. Prioritise work on removing the need for workarounds with safety implications or where A is represented as non-A (so that “the computer” allows one to move on). |
| 1.5.3. Mismatch of personality types  Examples: the stereotypical “geek” lacking social skills amongst some of the IT staff and the stereotypical people person among some of the healthcare staff. | Plan for working with a broad diversity of personalities. Create formats of interaction that improve understanding of each other’s roles and working styles and reduce stereotyping. Involve, as much as possible, staff with go-between roles and excellent boundary-spanning skills. |
| 1.5.4. Rationality of IT tools revealing irrationalities in the system | (also in Fragmentation of the health system) Seek streamlining but anticipate resistance and the need for significant negotiation. Irrationalities revealed are likely to be associated with valued services, well entrenched practices, and people’s jobs. |
|  |  |
| 1.6. Core tasks – peripheral tasks  (“Mixed Abilities Class dominated by a No Abilities Group”)  A family of challenges in projects which target both experts/specialists and non-experts (generalists, other types of specialists, and also the so called “low-skilled” workers).  Key features:   - specialists are expected to lead the way and provide support, but are too few; - non-specialists are numerous and of various professional backgrounds, various hierarchical levels, working in various services, with various levels of pre-existing knowledge, learning needs, ability to shape their role by themselves, and motivation to become engaged in professional development that is not (uniquely) crucial to their role; - in data sharing projects, the divide pertains both to IT skills and domain (e.g. end of life care) skills.   Parallels outside of health IT: any field where top specialists and non-specialists, experts and lay users, novices and advanced learners of various levels need to interact. Any field where individuals for whom an issue is of primary concern and a skill is a core skill are collaborating with users for whom an issue is of secondary concern and a skill is a peripheral or optional one. | |
| 1.6.1. Specialists duplicate work, as they still need to capture much more information for the needs of their service | Work on securing as close as possible integration of in-house tools for data capture and the shared tools for sharing data. |
| 1.6.2. Different types of non-specialists need different types of adaptation of the tools | Accept that high uptake will require tailoring to the needs of different services and professions. |
|  |  |
| 1.7. Work with highly sensitive, emotive, incendiary issues  (“The Daily Mail”)  Key features:   - minor missteps can grow into an unmanageable scandal that “kills” a project; - a rational, balanced response may not be possible and may need to give way to a strategy of appeasing fears and softening resistance; - parties who can benefit from the scandal will actively seek to do so; - most of the parties facing reputational damage will seek to distance themselves from a project, even if they have been close collaborators.   Examples outside of health IT: any issue that touches on conflicting views endorsed in one and the same society of right and wrong, good and bad, natural and unnatural (e.g. sexuality, abortions, welfare, immigration, euthanasia, individualism/ solidarity, etc.); any issue that touches on deep fears and high aspirations, often from the perspective of shared – but threatened – values within a society (death, money, identity, home, personal property, fundamental human rights, etc.). | |
| 1.7.1. A patient data sharing project is vulnerable to the impact of any external data sharing scandal | Possibly lie low during the external storm. Learn from work on managing scandal and responding to rumours. |
| 1.7.2. A data sharing project in a sensitive domain (here end of life care) is vulnerable to any external scandal in that domain | As above. |
|  |  |
| - 1. Technology – human users   (“Humans vs. Machines”)  Key features:   - technology can only fulfil its potential if handled by competent, well trained and responsible users; - technology needs to be adapted to what is most natural and intuitive for users as most will not go to great lengths to learn how to operate it well; - technology can be of little use, fail or even become dangerous if not operated well.   Parallels outside of health IT: engineering design; ergonomics; health and safety in the operation of dangerous equipment. | |
| 1.8.1. The quality of data sharing is irreducibly determined by the record keeping practices of users | Practically outside of the control of a single data sharing project. The community, however, can advocate for work in this direction, including in medical education.  The tension of seeing more patients/ spending more time with them as opposed to their notes will always remain. |
| 1.8.2. The quality of data sharing is irreducibly determined by the quality of classification systems and ontologies underpinning clinical IT systems | Practically outside of the control of a single data sharing project. The community, however, can support work on improving underpinning classification systems and biomedical ontologies.  Classification is an old philosophical chestnut. Some of its challenges are irresolvable. |
|  |  |
| - 1. Market forces and public good   (“*The Value(s) of Money*”)  Key features:  Tensions and overlaps between:   - the presence of powerful market forces in healthcare is problematic as motivations towards improving people’s health and well-being can come into conflict with profit-making motives; - the presence of market forces in healthcare can improve efficiency, value for money and accountability in public services; - pro bono work, social enterprises and university-led research projects can be positive balancing influences in public services that need to be more and more responsive to market forces; - practically any voluntary, pro bono or “free” work brings in demands and underlying agendas of its own.   Parallels outside of health IT: Any field where strong profit motives can skew the field’s primary goals and means (education, arts and culture, religion, etc.) although there is no in-principle contradiction between making a profit and the values of that field. Alternatively, any field where work that is normally highly paid is done for free. | |
| 1.9.1. Commercial data sharing projects can come into tension with the values of healthcare | Commit to practices of social and corporate responsibility. Align with and work to enhance ethical practices in the digital world. |
| 1.9.2. Publicly funded, not-for-profit data sharing projects can struggle with inefficiency – partly for reasons internal to the project and its accountability structures, partly because participating organisations do not pay to be part of them and, as a result, may not prioritise them. | Seek a more hybrid model – perhaps around a social enterprise – that has the best of both worlds. |
| 2. PURE DRIVERS  Descriptive name of type or subtype  (Suggested mnemonic/ metaphor) | **Proposed actions by health IT actors**  (partly derived from the data, partly involving new hypotheses) |
| 2.1. Pure drivers internal to IT solution   1. Ideal vision for the solution and its outcomes   (“Promise me the stars”)   1. Quality of the solution –functionalities, performance, user friendliness   (“Easy like a Sunday morning”)   1. Nature of the problem   (“Billion-people/ billon-dollar problem?”) | (Generic)  While these are the drivers most strongly within the control of a project team and, at first sight, most important to the success of a project (identifying an acute problem, developing and “selling” a compelling vision, developing and perfecting a solution), focusing on them at the expense of other drivers and challenges can sink a project.  (Actions in response to 1 opposite)  Positive visions, and expanding on them, is key. At the same time one also needs to draw bridges from current realities which may be (depressingly) far from the vision.  Rigorous evaluation projects need to be part of the work on developing data sharing solutions. The immense power of clear evidence in driving forward the implementation of innovation is well known, resulting in attempts to “evidence something” rather than conduct rigorous research and evaluation on it.  Importantly, negative evidence form early stages of the work, should not, in itself, result in discontinuation of promising project. Positive outcomes of innovation in complex contexts are difficult to capture. Moreover, they may take time to appear and accumulate, not least because the “final” version of the innovation is still emerging.  (Actions in response to 2 opposite)  A painfully well-known recommendation: simplicity and ease of use is paramount. Remember though that simplicity and ease of use can be a matter of excellent structure, layering, intuitiveness and visual decisions (and all the iterations, testing and high level of skills required for it) and not just of “keeping it simple” and “focusing on what matters the most”.  (Actions in response to 3 opposite)  Some conditions and specialities will, invariably, be associated with greater interest in associated data sharing solutions. Areas of significant healthcare costs and structured around multidisciplinary, multi-setting work will be amongst the most attractive. |
| 2.2. Pure drivers internal to the project development and implementation team  (“Dream team”)   - lead and leadership style - team composition - working style of team members and team as a whole | Recommendations around good leadership, good teams, and good team dynamics which are, so often, well known in theory and so hard to apply in practice. |
| 2.3. Pure drivers at the interface between the internal and external  (“Invisible threads”, or “weaving a spider’s web”)   1. Fit with and work on adapting features of the IT solution to a variety of dimensions of the external environment. 2. Embeddedness of the team (as a whole or as individual members) in relevant external structures. | (Actions in response to 1 opposite)  Data sharing projects need to plan for a number of sub-projects ensuring “good fit”. For instance, in the EoLC data sharing project, such work addressed:   - a national information standard for data sharing in End of Life Care - national and regional service monitoring and reporting requirements - local work on standardising data sharing - the local information management strategy - local incentive structures.   Such work requires further levels of adaptation, refinement and tailoring of a “ready” tool. Many of those requirements or good practices are not clear at the start of a project and come to it only gradually.  (Actions in response to 2 opposite)  Similarly, a data sharing project needs to be integrated in a variety of forums – digital health-related, healthcare services-related and ones at the intersection – that can inform its development and support its uptake. As above, many of the most relevant structures are not visible at the start of a project. |
| 2.4. Pure drivers external to the IT solution and team, but internal to the broader health IT ecosystem  (“Catching a wave”)   - other IT innovation that can be embedded in a data sharing project - trailblazing work of other data sharing projects - positive practices of individual organisations and health professionals around consenting patients to data sharing - the “Zeitgeist” – expectations and highly positive attitudes around certain types of data sharing. | Innovators have a natural instinct to use opportunities which the environment opens. Perhaps they have a lesser instinct in appreciating the importance of such contextual factors for the success of one’s work and seeing the truly co-created nature of it. |
|  |  |
|  |  |
| 3. OPPOSITIONAL AND/OR AMBIVALENT FORCES  Descriptive name of type or subtype  (Suggested mnemonic/ metaphor) | **Proposed actions by health IT actors**  (partly derived from the data, partly involving new hypotheses) |
| 3.1. Oppositional forces, temporary  (“The race is on”)  Cases where a driver is supposed to resolve a challenge once and for all:   - solution is internal to project - solution is external to project (e.g. single log-ins) - solution is at the interface between the internal and external (e.g. right timing). | This is the classic opposition of challenges and drivers where a discrete problem is identified and a discrete solution is developed. Unfortunately, while this is a major skill of project managers, it turns out to be a relatively minor way in which challenges and drivers appear and need to be handled in data sharing projects. |
| 3.2. Oppositional forces, recurring  (“The eternal battle of good and evil”)    Situations where a driver will need to be continuously available or periodically reinstituted so as to counterbalance a reoccurring challenge – e.g. in the case of training provision, communication, funding for support services, etc. | Data sharing projects cannot be time-limited projects, although they will naturally have different phases. They need to become and be adequately resourced as “business as usual”. |
| 3.3. Ambivalent forces, legitimate differences in clinical or paraclinical context  (“All in the open or skeletons in the cupboard”)  Situations where one and the same factor can be a driver and a challenge depending on the context:   - clinical context determined by the patient’s condition as marked by stigma or not (or other type of information that ends up in a patient record); - clinical context as degree to which the presenting situation (over-) determines clinical action (e.g. fractures and infections in otherwise healthy patients vs. long-term conditions); - context as the setting of care in the foreseeable future (e.g. cases where long-term hospital stay is envisaged and sharing with the community is not a priority); - context of perspective, skills and needs (compare, for instance, the case of sharing a patient’s record with the patient themselves or with staff at different levels of training, skills and seniority in the health service). | Consider the broad variety of contexts in which the records of a patient can be seen. To the extent to which it is possible, work to develop context-specific solutions.  Consider the patient safety challenges, as well as the numerous practical challenges, of selectively sharing and withholding information.  Accept that some of the tensions between different types of contexts are unavoidable. Be aware that this significantly affects opportunities to develop a satisfying data sharing solution when the data sharing is across a broad range of contexts. |
| 3.4. Ambivalent forces, divergence of values or complex entanglements  (“One man’s meat – another man’s poison”; “You don't find roses growin' on stalks of clover”^[[6]](#footnote-6)^)  Cases when one and the same factor appears as both a challenge and a driver.  This can be because of differences of perspective, framework of values, mindset, sets of needs and goals, etc. relative to which a factor is considered.  It can also be because some factors are exceptionally complex and manifest as inseparable entanglements of challenges and drivers.  Conflicting perspectives (and the same thing appearing as a challenge or driver) can be taken with regard to, for instance:   - *the nature of the data sharing tools along dimensions such as* more comprehensive vs. more concise; multifunctional vs. having a clear scope; more structured vs. more text-based; - *the foundations for the very possibility for data sharing* – dependent on whether the overarching preference is for greater independence or greater collaboration; - the *particular type of data sharing advanced –* e.g. in terms of who has editing rights and who does not; - perceptions of the project *progress and success;* - perceptions of the *support structures* in which the project is embedded or which it had set up – e.g. training programmes, collaborations with external stakeholders, supporting roles; - *the broader context* in which the project operates; - perceptions and self-perceptions of the work and working style of team members. | This parameter points towards one of the most important skills sets on a data sharing project – eliciting differences of opinion, finding creative solutions to them, resolving conflict, reaching a decision when consensus is not possible, i.e. a variety of aspects of effective communication in complex and/or highly charged contexts.  Create structures and processes which enable everybody on the project team or stakeholder group to be heard. Pay particular attention to team members, settings, parties to the project, etc. which have (or experience themselves as having) less authority.  Create an atmosphere that allows for challenges to the dominant or senior opinions, unpalatable concerns about the project, or personal struggles around the work to be aired.  At the same time create structures, processes and standards which enable a decision to be made, even if there is significant disagreement and consensus cannot be reached. |
| 3.5. Ambivalent forces, inequality considerations  (“Princes and paupers”) | Consider the inequality consequences of a data sharing project, particularly as arising in cases when the data sharing is organised around a dominant system and, by default, excludes or disadvantages patients of services that are using other systems. |
| 3.6. Ambivalent forces, unintended consequences  (“Dark side of the moon”)  Cases when a factor is overall a driver of positive outcomes but has unintended consequences or elusive but important negative aspects. For instance:   - greater access to colleagues’ records can result in more clinicians feeling their work is interfered with or their judgement questioned; - more informational support could mean that some clinicians experience greater pressures to make decisions they feel uncomfortable with; - more informational support could mean that some clinicians become unjustifiably confident in their ability to make decisions they are not fully prepared to make; - informing colleagues that one has initiated an aspect of care (e.g. conversations around the end of life) could result in recipients considering such tasks as “addressed”; - access to a well-established clinical history could prevent the fresh perspective where the solution resides; - acting on outdated external information could be far more dangerous than acting from the situation at hand. | Be acutely aware of the possibility for and evaluate the presence and extent of unintended consequences. |
| 3.7. Ambivalent forces, reversals of the negative  (“From defect to effect”, “Rainbow after the rain”)  Cases where:   - a negative experience brings about a positive learning – e.g. when people strongly opposed to data sharing experience the negative consequences of not sharing; - a negative expectation does not materialise – e.g. when a feared conversation ends up welcomed and positive; - a flaw can be used to one’s advantage – e.g. GPs can be fiercely independent, but when their competitive streak is pricked, they can easily join the crowd in order to compete with it. | There are some challenges that take a turn for the positive, even if no specific action is taken. There are also challenges that can be turned on their head through lateral thinking and creativity. |
| 3.8. Ambivalent forces, conflicting or unclear evidence  (“Unknown quantities”)  Factors which appear as challenges or drivers due to insufficient reliable knowledge/evidence. | Create a culture of independent evaluations.  The majority of data sharing projects will be either service development or commercial projects. They will not have the capacity for academic evaluations and research. Create alternative structures that enable aspects of their data and learning to be captured and shared. Facilitate collaborations with academia. |
| 3.9. Ambivalent forces, vicarious learning or feeding on the failures of others  (“Digital vampires or digital sages”) | The success of a data sharing project (and not only) sometimes passes through the “corpses” of other data sharing projects. It is important to learn from the failures of others; preferable not to rejoice in them, but, realistically, this too will happen in much of the work on digital health. |
|  |  |

1. Mnemonics/ metaphors are offered for factors that are rarely formulated in the health IT literature (e.g. “resource limitations” in the health service is not accompanied by a mnemonic, as it is hard to forget) or at the level where most of the preparation aimed at addressing a factor is likely to occur (e.g. we use “Daily Mail” for work with sensitive, emotive, incendiary issues and do not offer metaphors at the lower levels). [↑](#footnote-ref-1)
2. As the data sharing project under investigation had the in-principle (at least) commitment of the local health economy from the start, we do not cover actions needed by “complete outsiders” to the health service. [↑](#footnote-ref-2)
3. Some of the discourse on radical innovation goes along the lines of a well-known Eurythmics song: *Hey, Hey, I saved the world today/ Everybody's happy now/ The bad thing's gone away/ And everybody's happy now/ The good thing's here to stay/ Please let it stay* (Lennox and Stewart, 1999). The complete lyrics, music and video speak of a far greater complexity though. [↑](#footnote-ref-3)
4. “Banality of evil” is a phrase coined by the philosopher and political theorist Hannah Arendt to describe some of the causes of Nazi atrocities. It is used to convey the idea that the perpetrators of some of the most evil acts in history were not monsters, but bureaucrats who dutifully and uncritically obeyed orders. The use here deviates from this meaning but aims to capture the idea of significant challenges, deep frustration, and “death” of many data sharing projects resulting from the need for numerous repetitive, simple, bureaucratic acts and associated obstacles. [↑](#footnote-ref-4)
5. Phrase suggested by Professor Mary Dixon-Woods, 06 Apr 16 presentation, Clinical School, University of Cambridge. [↑](#footnote-ref-5)
6. From the song (I Never Promised You a) Rose Garden (original of Joe South, 1967): “But you don't find roses growin' on stalks of clover/ So you better think it over” [↑](#footnote-ref-6)
