# Supplementary material for: From “wading through treacle” to “making haste slowly”: A comprehensive yet parsimonious model of drivers and challenges to implementing patient data sharing projects based on an EPaCCS evaluation and four pre-existing literature reviews
Source: PLOS Digit Health. 2024 Apr 1;3(4):e0000470. doi: 10.1371/journal.pdig.0000470 (PMC10984410; doi:10.1371/journal.pdig.0000470)
Supplement: S4 File — (DOCX) [file pdig.0000470.s004.docx]

**S4 File: Conceptualising “challenges” and “drivers”**

**Box 1:** **Considerations around defining “challenges” and “drivers”**

**Starting points in the analysis (working definitions):**

- **Challenges**: factors that slow down, hamper or completely block the progress of data sharing projects (or more diffuse work around data sharing), their uptake by users or the achievement of their outcomes.
- **Drivers:** factors that help progress, speed up or make easier the progress of data sharing projects (or more diffuse work around data sharing), their uptake by users or the achievement of their outcomes.

**Considerations and caveats against an “intuitive” understanding of challenges and drivers:**

- it is not the case that drivers are always “good” and challenges always “bad”;
- it is not the case that challenges and drivers tend to come in pairs where one polarity negates the other;
- it is not the case that drivers and challenges fall into clear-cut, distinct categories. There can be significant grey zones between challenges and drivers;
- it is not the case that challenges and drivers, if identified in a reliable way, are “real” or “objective” or that it necessarily matters. “Imaginary” and “subjective” challenges and drivers can also cause action or inaction.

**Considerations and caveats informed by the empirical work** (largely specifying the above)**:**

- One and the same complex factor is often both a driver and a challenge – typically in terms of different aspects of it and/or relative to different goals. This complexity is not difficult to see once pointed out, yet in the discourse of a single individual, in groups of similar individuals or in aspirational documents it is practically missing. Factors appear as non-ambivalent drivers or challenges unless a person or a group have been forced to question their perspectives.
- Some drivers and challenges are misinformed (and in this sense “not real”, “imaginary”) but still advance or hamper the progress of a project. For instance, a misguided belief that having an EPaCCS (Electronic Palliative Coordination System) was a national requirement was helpful to the EoLC data sharing project in its early stages (the actual national requirement was that if an area had an EPaCCS, it had to be consistent with the national information standard). To complicate matters further, what was once a misperception became a reality: EPaCCSs became mandatory in England.
- A driver is not necessarily collaborative and harmonious (i.e. a driver can be “bad”). For instance, several interviewers mentioned how strongly negative attitudes towards a contemporaneous data sharing project helped advance the EoLC data sharing project.
- A driver can support patient data sharing, but otherwise be problematic in our broader lives. For instance, having “everything … less boundaried and more accessible in everyday life” can drive forward patient data sharing but, from a broader perspective, be both a driver and a challenge.
- At times, challenges can reflect unrealistic expectations. The primary challenge can thus be the perception of data sharing as presenting us with a challenge rather than data sharing presenting us with a challenge that is within its power to resolve*.* For instance, the quality of data sharing is strongly dependent on the quality of the source records, the underpinning classification systems, and the ways codes from the latter are ascribed by users to the former. Advances in data sharing alone cannot resolve such fundamental challenges.
- It is difficult, at times impossible, to rank challenges and drivers in terms their importance or power and prioritise work on this basis. For instance, apparently minor challenges or drivers could contribute to reaching a “tipping point” and either lead to a step change or “break the camel’s back”. Similarly, serious challenges can be resolved by concerted effort and thus end up taken care of, unlike far more trivial but enduring matters that block progress.

**Box 2: Key categories of challenges, drivers and ambivalent forces from the four literature reviews**

*“Key categories” has been operationalised as drivers and challenges highlighted in the abstracts of the reviews and/or accompanied by the largest number of references in the results section or main results table of the review article.*

**Key challenges from the literature reviews which fit our “pure challenges” category:**

- **technical issues** (key in 3 reviews);
- **privacy and security issues** (key in 2 reviews);
- **the existence of alternative data sharing solutions** (key in 1 review);
- **generic challenges of the IT infrastructure and systems interoperability** (1);
- **the need for culture change** (1);
- **chronologically, the disruption at initial implementation** (1).

**Key drivers from the literature reviews which fit our “pure drivers” category:**

- **high quality leadership, management and clinical champions** (3);
- **users’ technological skills** (2);
- **ongoing training** (1);
- **positive attitudes** (1);
- **time availability and good timing** (1);
- **awareness of the intertwining of technical, social and organisational factors and allowing for new ways of working to emerge** (1).

**Key challenges and drivers which appear in dyads and thus fit one subtype (out of 9) of our “oppositional and/or ambivalent forces” category:**

- **inefficient workflows** (key in 3 reviews) **vs. thoughtful workflows** (key in 1 review);
- **user needs not met or sensitivities accounted for, often resulting from limited involvement** (3) **vs. user involvement** (2)
- **costs and financial sustainability challenges** (1) **vs. financial support** (2)
- **inadequacies of the information provided** (1) **vs. better patient information** **(more complete or adaptive)**.

Accompanies manuscript: Petrova M and Barclay S. *From “wading through treacle” to “making haste, slowly” in patient data sharing:* *A comprehensive yet parsimonious model of drivers and challenges to implementing patient data sharing projects based on an EPaCCS evaluation and four pre-existing literature reviews. PLoS Digital Health 2024.*

Accompanies manuscript: Petrova M and Barclay S. *From “wading through treacle” to “making haste, slowly”:* *A comprehensive yet parsimonious model of drivers and challenges to implementing patient data sharing projects based on an EPaCCS evaluation and four pre-existing literature reviews. PLoS Digital Health 2024.*
