# Supplementary material for: From “wading through treacle” to “making haste slowly”: A comprehensive yet parsimonious model of drivers and challenges to implementing patient data sharing projects based on an EPaCCS evaluation and four pre-existing literature reviews
Source: PLOS Digit Health. 2024 Apr 1;3(4):e0000470. doi: 10.1371/journal.pdig.0000470 (PMC10984410; doi:10.1371/journal.pdig.0000470)
Supplement: Attachment — Submitted filename: Review_PDIG-D-21-00135.pdf [file pdig.0000470.s008.pdf]

Manuscript Number: PDIG-D-21-00135

Title: From “wading through treacle” to “making haste, slowly” in patient data sharing: A comprehensive yet parsimonious model of drivers and challenges to data sharing based on an EPaCCS evaluation and four pre-existing literature reviews

Short Title: Drivers and challenges to patient data sharing

This manuscript proposed a model with >500 challenges and >300 drivers for implementing Electronic Palliative Care Coordination System (EPaCCS) and other data sharing projects. Also, authors have concluded that one should focus less on refining their IT tools and more on shaping the social interactions and structural and contextual parameters for data collection.

I would like to summarize my comments in few points as:

1. Entire manuscript is written in a very subjective way or “narrative style” for data collection process, still reader will not find any glimpse of data, or any data processing methods, and outcome in table or figure.
2. The primary data source, which is 40 in-depth interviews, approximately 300,000 words is quite ambiguous and not suffice to make any conclusion.
3. In this modern era, where IoT devices are gaining enormous popularity, in my opinion (unlike the authors) one should give more importance to data collection mechanisms, devices, and IT tools for seamless, accurate and precise data collection. Also, nowadays remote, contactless methods of data collection are easier, cheaper, convenient, etc., so social interactions and other things are becoming secondary.
4. This manuscript could be written in simpler English, but abundant use of idiom, phrases, and words from classical English literature makes it hard to understand and may be not a good choice for a technical journal article.

Overall, I will not recommend this manuscript.
