# Supplementary material for: From “wading through treacle” to “making haste slowly”: A comprehensive yet parsimonious model of drivers and challenges to implementing patient data sharing projects based on an EPaCCS evaluation and four pre-existing literature reviews
Source: PLOS Digit Health. 2024 Apr 1;3(4):e0000470. doi: 10.1371/journal.pdig.0000470 (PMC10984410; doi:10.1371/journal.pdig.0000470)
Supplement: Attachment — Submitted filename: Review_PDIG-D-21-00135_R3.pdf [file pdig.0000470.s010.pdf]

Manuscript Number: PDIG-D-21-00135R3

Title: From “wading through treacle” to “making haste slowly”:A comprehensive yet parsimonious model of drivers and challenges to implementing patient data sharing projects based on an EPaCCS evaluation and four pre-existing literature reviews

Short Title: Drivers and challenges to patient data sharing

I thank authors for considering my comments positively, answered the comments, and improved the manuscript accordingly. After revision of the manuscript, my comments are:

1. Thank you for answering my comment, and now the type of the paper is clear to me after including the paragraph – “As the case study project… …. Interested in high-level technological innovation.”
2. After clarifying the scope and conceptual nature of this work, the goal of this conceptual framework is understood. Therefore, previous comment is nullified now.
3. After adding the paragraph – “Most broadly,…… on perfecting them.” – it is clear that this work focuses on data sharing projects/ platforms rather than actually collecting the data.
4. In the revised manuscript, after following suggestions and changes made, I would recommend language of the manuscript is an important way to express the goal, methods, and results that will help attract the readers who will most benefit from this paper. Also, all quotes into boxes and make their reading more optional is a good approach.

Now, I find that this paper has a great potential to contribute on concept level of patient data sharing in an interesting narrative style.
