# Supplementary material for: From “wading through treacle” to “making haste slowly”: A comprehensive yet parsimonious model of drivers and challenges to implementing patient data sharing projects based on an EPaCCS evaluation and four pre-existing literature reviews
Source: PLOS Digit Health. 2024 Apr 1;3(4):e0000470. doi: 10.1371/journal.pdig.0000470 (PMC10984410; doi:10.1371/journal.pdig.0000470)
Supplement: Attachment — Submitted filename: Petrova_Barclay_Response_Reviewers_22Sep22-R.docx [file pdig.0000470.s005.docx]

PDIG-D-21-00135
Petrova M and Barclay S. From “wading through treacle” to “making haste, slowly” in patient data sharing:
A comprehensive yet parsimonious model of drivers and challenges to data sharing based on an EPaCCS evaluation and four pre-existing literature reviews

*Dear Dr Mathur, Dear Editorial team members,*

Thank you for soliciting such helpful reviews to our paper and for giving us the opportunity to revise it. Our responses to the reviewers’ comments are below. We hope we have addressed all concerns adequately.

We look forward to hearing back from you in due course.

Yours sincerely,

Dr Mila Petrova, and on behalf of Prof. Stephen Barclay

| **Comment No** | **Reviewer’s comment** | **Authors’ response** |
| --- | --- | --- |
| **Reviewer 1** | This is an interesting paper (although quite long). I do have some remarks and suggestions for improvement or further discussion: | Thank you for taking the time to review our paper, for the positive feedback and helpful criticisms/ suggestions. We hope we have addressed them adequately and that by reducing the paper by about 2,500 words and restructuring it further we have made its length less of an issue. |
| 1.1. | Data availability: I understand you cannot share data on an individual level because of privacy concerns, but are you able to share any aggregated data somehow? Perhaps text can be processed by NLP and summarized? | We have provided almost 30,000 words of additional quotes in Supplementary file 2. While this is not “all the data” which, as we agree, cannot be openly shared because of its sensitivity, it is close to being most of the relevant “signal” in the data, excluding over-represented, repetitive signal.  We really liked the idea of using NLP methods to present a different perspective towards the data, but we did not have the capacity to set up such a project in the time we had for revisions. One of us has used NLP methods in the past, but we are not versed in current approaches. We would also need to file a study amendment with the ethics committee, as it would be new use of the data. We are, however, really grateful for the suggestion and will consider using this dataset to explore the utility of current NLP approaches for our work with large qualitative datasets. |
| 1.2. | Introduction: data sharing in the UK might be much different from other countries. Can you provide some introduction as to how healthcare and data sharing are setup in the UK? | We have now provided a new paragraph on the setup of UK healthcare and data sharing within it, right before the Methods and Materials section. Thank you for this suggestion. |
| 1.3. | General comment: I noticed that GDPR (or the UK implementation of it) is not mentioned at once in the paper. Does GDPR influence data sharing in any way? | GDPR had not come into force by the time we finished data collection. We have now added a sentence to the Limitations section to acknowledge that this element of the big picture is missing. |
| 1.4. | General comment: There are some expressions that are perhaps clear to UK citizens but unclear to a global public. Examples: '1001 nITes', 'In the deep waters – toes in the water – high and dry', etc. (but many more!) Please explain or leave out. | Thank you for offering a recommendation to this effect. We were hesitant about including those metaphors/mnemonics – which were one of our solutions to facilitate retention (and, also, to make reading such a long document a bit more engaging). We have now removed all of them from the main text. |
| 1.5. | General comment: (lack of) interoperability is mentioned. Would following the FAIR principles (<https://www.nature.com/articles/sdata201618>) help solve some of the data sharing problems? | It certainly will help but, in our framework, interoperability was just one of 800+ challenges and drivers, using figures from our intermediary analysis. We would like to preserve the integrity of the framework and offer solutions at this broader level, which we have done in the recommendations for action. Previously these were in the 19 pages of Supplementary file 3. We have now also included shortlists of recommendations as boxes (3 of them) in the text. We have pointed to the lack of data on new initiatives in interoperability in the Limitations section. |
| 1.6. | 1.2.2. Fragmentation: If even in the UK (with its NHS) data there is a high level of fragmentation, how bad will this be in other countries? | We agree but have refrained from discussing it, as it is an empirical question if having a national health system of the type of the UK NHS results, necessarily, in a more unified healthcare system than other national models. |
| 1.7. | Discussion: Implications for research: there are many papers about data sharing in research, which would help to put this section in context, e.g. <https://pubmed.ncbi.nlm.nih.gov/32349396/> and <https://pubmed.ncbi.nlm.nih.gov/27096325/> | Thank you for those suggestions. We read the papers with interest; they were rich in detail and thought-provoking. Reading them, however, made us realise that there are further meanings of data sharing for individual care which have been masked by the definitions we were using, as how we use data sharing for individual care is different to how those papers use it, and outside the scope of our study. We have now made the following clarification:  *The above distinction also masks differences between sharing data to be used more or less directly by other health professionals as opposed to first subjecting it to computational analysis as in precision medicine [3] and between sharing data collected in routine clinical practice as opposed to generated in research studies.*  This is followed by a further clarification of the type of data sharing this study addresses: *data sharing for individual care where the shared data are collected in routine clinical practice and where they are (to be) used directly by other health professionals.*  We believe that the reviewer was pointing towards discussions of data sharing where individual data were typically collected for research purposes and/or analysed within the context of precision medicine. This is beyond the scope of our study, but we are grateful for the opportunity to clarify the object of our study further. |
| 1.8. | Line 1334-1344: As you state yourself, interviews were done nine-seven years ago. In the meantime, there have been many developments in AI, taking over human tasks. Do you see any use of AI in data sharing? | We have now added a comment to this effect in the Limitations section. There are definitely opportunities to use AI in data sharing, but this is not an issue we have data on. |
| 1.9. | Line 295: "though" -> "through" | Thank you for noticing this: we have amended the text. |
|  |  | Once again, thank you for taking the time to review our paper and for the helpful suggestions, especially in terms of specifying its scope and limitations better. We hope we have addressed your comments satisfactorily. |
|  |  |  |
| **Reviewer 2** | Thank you for the opportunity to review this paper. The paper aimed to identify the challenges and drivers encountered by Electronic Palliative Care Coordination Systems (EPaCCS) in comparison to those in other projects on data sharing for individual care. It aimed to propose a framework of recommendations for steps forward. The focus of this paper is important and timely within palliative care, and the manuscript contains some important messages on considerations for the implementation of EPaCCs (and other similar electronic systems) into routine practice. | Thank you for reviewing our paper. We really hope it will help to advance work on EPaCCS, even if, potentially, in new formats where they are no longer standalone systems. |
| 2.1. | The authors are to be commended for a large in depth piece of work. However, at 13,500 words, the manuscript is too long/unmanageable to be practically useful for those interested in the topic area. | We have sought to condense the paper further and have now lost about 2,500 more words (even if we added clarifications as requested by both reviewers, i.e. our original text was shortened by about 3,000 words). As it is, however, a comprehensive model of a very complex issue – we started form 1,800 parameters – we will not be able to bring it down to a standard 4,000-word paper, unfortunately. We have tried to develop much shorter versions, with the result that the appendices carried too much of the substantive contents.  We are envisaging some simpler dissemination formats, such as a blog on *“50 recommendations for teams working on data sharing projects”*, but we need to also have a format which presents the underpinning data as succinctly as possible yet without losing contents.  We hope that the reduction in length and some of the restructuring makes it a more attractive paper. |
| 2.2. | While at a 'micro' level the writing is clear, the bigger picture of the paper is extremely difficult to follow. | We have tried to clarify our goals even better in the Introduction and Methods section. We have also added 3 boxes of recommendations within the text. Hopefully, this helps see the bigger picture more clearly. |
| 2.3. | The research team have gone to considerable lengths in pulling together large amounts of data. However, the study design is confusing, particularly the way in which the qualitative analysis (primary data collection) links to the existing literature analysis. | We have further revised the Methods section to justify and clarify the approach. While it is an unusual approach, not least because it is very time consuming, we would argue that it is a highly robust approach that adds a further test to the data and its interpretations, seeking to link current knowledge to pre-existing knowledge parameter by parameter. The previous literature expanded our analysis framework and helped test our findings against pre-existing findings. This meets scientific requirements for cumulation of knowledge in far more stringent ways than simple comparisons with the literature in background or discussion sections. |
| 2.4. | The real value of this paper lies in the primary qualitative data, with an impressive number of interviews collected, and how this informs recommendations for ways forward. The authors might consider if the paper could be improved and made more manageable by integrating findings of the literature review into the introduction / discussion. | Thank you for the positive words about our primary data. We were extremely grateful to our participants for the depth and honesty of the interviews. Please see above our comment on the integration between the interview findings and literature review findings. |
| 2.5. | The recommendations for action (S3) are important, but felt lost in the supplementary data. | Thank you for this comment and the encouragement to come up with a creative solution. An earlier reviewer made a very similar comment, and we have wrestled with the challenge of responding to comments that the paper is too long while also being advised that some of our supplementary material is too valuable to externalise. We now offer three boxes of shortlists of recommendations (one for each of the high level parameters – pure challenges, pure drivers and ambivalent forces) to include as part of the main text, with the complete list of recommendations preserved in Supplementary file 3. |
| 2.6. | The authors may also wish to consider dropping the use of popular culture metaphors and/or mnemonics; these were confusing and detracted from the key points being made. | We have now dropped them; thank you for your recommendation. We have only left them in one of the appendices as we feel they (or similar mnemonics they might prompt users to develop) may help with memorising the elements of the framework. |
| 2.7. | Finally, for reader to be clear on how the research was conducted, alongside make judgements of its methodological rigour, it may also be beneficial for the methods section to be integrated into the main body of the article (depending on journal styles). | We have condensed the methods section, partly through editing, partly through externalising some of its contents fully to the Methods appendix (e.g. about Patient and Public Involvement) and have restored it to its more natural place, before rather than after the Results. In the previous version, we had used an opportunity allowed for by the journal so as to address the challenge of the length of the paper by “downgrading” a section which interests researchers, but not practitioners, who often skip it. |
|  |  | Thank you once again for the time you have taken to review our paper and the helpful suggestions. We hope we have addressed your concerns effectively. |
