# Supplementary material for: From “wading through treacle” to “making haste slowly”: A comprehensive yet parsimonious model of drivers and challenges to implementing patient data sharing projects based on an EPaCCS evaluation and four pre-existing literature reviews
Source: PLOS Digit Health. 2024 Apr 1;3(4):e0000470. doi: 10.1371/journal.pdig.0000470 (PMC10984410; doi:10.1371/journal.pdig.0000470)
Supplement: Attachment — Submitted filename: Petrova_Barclay_Response_to_Reviewers.docx [file pdig.0000470.s006.docx]

Dear Dr de la Hoz, dear Editorial Team members,

Thank you for the re-review of our paper “From “wading through treacle” to “making haste, slowly” in patient data sharing: A comprehensive yet parsimonious model of drivers and challenges to data sharing based on an EPaCCS evaluation and four pre-existing literature reviews” (PDIG-D-21-00135R1). We were grateful to the reviewers for having taken the time to consider our manuscript again. In the table below, we address the outstanding comments of Reviewer 3.

We hope our responses satisfy the reviewer’s and editorial expectations and will be looking forward to hearing back from you in due course. Thank you once again for soliciting such helpful, constructively critical reviews.

Yours sincerely,
Mila (Dr Mila Petrova), and on behalf of Professor Stephen Barclay

| **Reviewer 3 comments** | **Authors’ responses** |
| --- | --- |
| The manuscript is excessive long and at times difficult to read, convoluted and without focus. The subject was EPaCCS but even arguments like insufficient syringe drivers are part of the results. | Thank you to the reviewer for re-reviewing our manuscript. We are sorry that our previous revisions have not been enough to make our paper more enjoyable to read. We have edited and shortened the paper further, trying to minimise marginal points and complex expressions and sentences. We have, however, had different goals to the ones the reviewer seems to have deduced and there are limits to which we may be able to meet their expectations.  This is a “big picture” paper. We started the model from 1,800 challenges and drivers to data sharing and reduced it to 22 high level categories, having gone through numerous revisions, across several years, aiming at crystallising key messages. As stated on p.6, we sought to “generate a comprehensive yet parsimonious model of the challenges they [data sharing projects] face and the drivers that enable them.” As stated there again, with some new revisions to the sentence to make sure it’s clear that our goal is not EPaCCS-specific, “We also had a strong applied goal: to derive specific and non-trivial recommendations for advancing data sharing projects, particularly ones which are in relatively early stage of their development and implementation.”  We hope we have made it clearer that our goals were not-EPaCCS specific. Unfortunately, a big picture paper is never going to be brief and easy to read.  We excluded the reference to syringe drivers. |
| -when comparing data sharing (UK concept) with Health Information Exchange, with the term more popular in the US why is not just one short paragraph ? | Related to the point above, data sharing is, ultimately, the core concept of the study. We needed to clarify alternatives in the terminology used as well as different uses of the same concept, at times vastly different. We have edited this paragraph further to improve its clarity. |
| -when describing the NHS, why so much? the important fact is not that every big organisation is divided in sections and subsections, but the fact each one is free to choose their own software, causing interoperability issues. | Thank you for this query. The information about the NHS was added on recommendation of an international reviewer. We have now shortened the paragraph but agree fully with the reviewer who made the recommendation that such background information is helpful to international readers. |
| -As pointed, a lot has changed since the pandemic, even EPaCCS and Respite templates. So much detail for information that can be considered too old and not reflecting fully the current environment | As acknowledged on p.8, some aspects of the data are, indeed, old and no longer relevant post-COVID, and we have adjusted reporting accordingly. If we are guessing correctly the main section the reviewer is referring to here, it concerns the multiplicity of alternatives to EPaCCS, many of which have changed. In this case, we have left this detail as it illustrates the multiplicity and types of alternatives. Even if their specifics have changed, the types of alternatives remain. |
| -even table 1, so much information, like recruitment details, what's its purpose? | We have reported recruitment in so much detail as a matter of research transparency, to enable readers interested in the methodological aspects of the study to evaluate the particular strengths and weaknesses and potential for bias in our sample. We understand that this level of reporting is of interest primarily to methodologists, but it is a matter of research transparency and integrity to include it. We have already moved multiple aspects of the methodology in an appendix to simplify the presentation of the method in the text. |
| -some of the respondents comments have no interest, and they are confusing. | Thank you for the challenge to shorten the paper further. We have now deleted a range of quotes. (Whenever there is “object deleted” in the manuscript, this is a box of quotes lost or reduced. Apologies it does not show more clearly. The track changes function loses “the object” only; it does not show the text lost. The deletions are over and above the 600+ words reduction of word count of the main text.) |
| -1.2.3. Constant transformation, 1.2.4. Environment deprioritising “extras” and 1.3. Work in large impermanent teams are part of the same, indirect factors. Software is always evolving, as pointed, staff is always changing (A very large training institution). The focus should be on how to make progress on EPaCCS easily accessible and understood by those who could benefit from using it. | We agree that these are broad, one can say “indirect” factors, but we argue that they are crucial for the success or otherwise of a project, which, in turn, makes them directly relevant. Strategies to address them need to be factored in systematically in setting up data sharing projects and we have identified a range of these in the appendix on recommendations and, more briefly, in the boxes on recommendations.  Again, as mentioned in our response to the first point, our focus is not on EPaCCS only. If anything, in the discussion we propose that relative to our findings, EPaCCS may need to be phased out. Our goal is to support the set-up and implementation of any type of data sharing project through offering a comprehensive yet economical model of associated challenges and drivers. |
|  | Thank you once again for reviewing our paper. We hope that, with its newly introduced changes, more readers will find it easier, clearer and more enjoyable to read. |
