# Supplementary material for: From “wading through treacle” to “making haste slowly”: A comprehensive yet parsimonious model of drivers and challenges to implementing patient data sharing projects based on an EPaCCS evaluation and four pre-existing literature reviews
Source: PLOS Digit Health. 2024 Apr 1;3(4):e0000470. doi: 10.1371/journal.pdig.0000470 (PMC10984410; doi:10.1371/journal.pdig.0000470)
Supplement: Attachment — Submitted filename: Review Letter_HIE_2023.7.14.docx [file pdig.0000470.s007.docx]

Dear Dr. Petrova and Dr. Barclay,

Thank you for the opportunity to review your work. I think the paper addresses an important topic and is commendable for its work to bring organization and clarity to extremely difficult to define concepts and observations. In particular, the findings resonated with my own lived experiences in attempting to innovate healthcare systems and I do believe your research advances the field of health information exchange. My key takeaway is this paper is answering in detail what it is that makes the actual implementation and use of these highly promising tools so challenging.

While the findings in this paper are worthy of publication, I do express similar concerns as the previous reviewer. I found the paper’s writing or communication style needs to continue to be edited for clarity and further polish, particularly in the Results and Discussion section. I hope my comments and feedback below will help to support the further advancement of this paper.

Paper Title and Description – A recommendation to consider is to shift the framing of this paper from an HIE paper to be about the incentives and challenges of digital health innovation more generally (utilizing as EPaCCS as the case study/source materials). Based on the title and “Aims” statement, I expected the paper’s content to be more on the technical specifics of data-sharing itself and adjustments of practice operations to incorporate new data sources into the day-to-day workflows. As currently described in the title, this paper will likely attract the attention of technical HIE experts who are seeking that highly tactical guidance. I would consider ways to ensure the title and abstract contain key words (implementation science, project management, healthcare innovation, digital health, etc.) that will draw the attention of a broader audience of healthcare innovators and digital health leaders – those who would benefit the most from the studies findings.

Author Summary – I find the Author Summary clear, compelling, and concise.

Minor Revision: Please add a source to the sentence “ the evidence in their favour is uncertain.”

**Introduction**

Comment: I find the Introduction clear and well-written.

Recommendation: Consider retitling this section “Background” as oppose to “Introduction”. Per the level of detail given on HIE, NHS, and EPaCCS given, the word “Background” helps indicate to the reader that we are out of overview descriptions and getting into details. This is a personal preference though.

Minor Revision: Please add a source to statement “which are only partially supported by inconclusive and generally low-quality evidence”.

Minor Revision: You have a wonderful aim statement written within the introduction: “We also had a strong applied goal: to derive specific and non-trivial recommendations for advancing data sharing projects, particularly ones which are in relatively early stage of their development and implementation.” I would recommend working this language into your Aims statement within the Abstract. I think it is an important goal that will help attract the readers who will most benefit from this paper.

Minor Revision: Please relocate the paragraph labeled “Context of the broader study and service development project” to the Introduction section directly after the paragraph describing NHS. I think the description of EPaCCS better lends itself to the Introduction section, would flow nicely after the description of NHS, and creates a more organic ending to this section of the paper.

Minor Revision: Please give 1-2 paragraphs to describe the type of patient data shared via EPaCCS and across the number and type of healthcare organizations involved (home health nursing, etc.). Also, any known successes/outcomes of EPaCCS for advancing care. How much data is regularly moving across the system and/or how often is it accessed by providers? Specifically, this would be a good point to describe the template that is later referred to in the paper. Noting this information may be available in S1, the likelihood of a reader stopping to review that document is slim and such information at the outset will help to provide the additional context needed for understanding the results.

**Materials and Methods**

Revision: My major concern is the 8 - 10-year timelapse of the data collected that provides the foundation of the findings for this paper. As a reader, upon learning the age of the dataset, one immediately starts to discount the potential relevance of the findings given a) the advancements in data sharing technology b) the increase user savviness with technology and b) the massive shifts in digital health fueled by the pandemic. Please include a justification (maybe in a text box cutout) to give more rational for use of this dataset. Why this dataset as oppose to conducting a new round of interviews? What indicators do you have that the dataset is still relevant to today? My suspicion is that this is an extremely high quality/hard won dataset that had more to give and deserved to be further analyzed despite its age. The reader needs more justification here than solely acknowledgement of the datasets age in order to continue reading with the confidence that there are valuable findings to come.

Revision: You write “We acknowledge the length of time which has elapsed since data collection and have deprioritized the reporting of dated issues (e.g., claims around the need for a “culture change”, which the COVID-pandemic achieved).” Per reproducibility of this research and to build confidence in the dataset, can you provide more detail on how dated issues were identified and eliminated from the dataset?

Minor Revision: Please provide more detail on the interview time period and the stage in the implementation at which the interviews were conducted. Per the aim to support projects in their early development, it would be helpful to know if the interviewees are responding based on the initial implementation time period or several years into a fully mature project? Also, if the interviews were spread out over a long period of time, were interviewees responding based on their experiences at different points in the work.

Minor Revision: Were all the interviews conducted in English? Do you have any demographic data of the interviewees themselves (particularly the patients/carers)? Per the increased prioritization of Diversity, Equity, and Inclusion (DEI) across healthcare, it is helpful to know if this data set represents a broad or more niche segment of healthcare. If highly niche (a healthcare organization that serves primarily high-income individuals or primarily non-English speaking patients), then I would address the need to gain perspectives from a wider variety of organizations in the limitations section of the paper as well.

Revision: Please provide more detail on how the literature reviews were selected, the publication dates of the literature reviews themselves, and the range of publication dates of the 135 source studies reviewed. As a reader, I’m curious to know if the literature reviews cover material written in and around the interview time period or if they cover more recent literature between 2015-2023. If more recent literature is included in the literature review, I would include a note that the literature review was conducted to ensure the applicability of the interview content and further strengthen the relevancy of this paper. If the literature review is only up to 2015, I would provide justification for this decision.

Revision: Please provide the time period when the interview analysis and synthesis of interview and literature review data was conducted (page 12).

Results

Overall Comment: The results section is where the paper’s writing - at times - degrades in quality, efficiency of language, clarity, and structure. I would encourage that the author’s keep editing this section to ensure the writing is as solid as the two previous sections. Further, I would rethink the aim of this section from covering every detail uncovered in the research to instead focus on how to “teach” the reader the overview of each challenge/driver/ambivalent force. For the details, I would shift the text to supplemental documents when needed to provide immense detail on a framework component. I recommend this approach in order to make a more manageable and approachable paper for a reader to cover in one-sitting. The additional materials are there for the person who needs to study the content at its most nuanced level.

Recommendation: I would consider listing the “pure drivers” before the “pure challenges” in the paper. This order better matches the flow of an implementation project as the incentives for conducting such work come before the challenges reveal themselves during the actual implementation. This is a personal preference though per how my brain is wired to order drivers vs. challenges.

Revision: Given the overall length of the “results” section, I would include a short bullet point list or a table listing out the titles of each of the framework components and potential a 1-sentence description of each component. This addition will help prepare the reader for the content to come and also give them a chance to skip forward to the section of most relevance if needed.

Revision: The section 1.1 “Radical Innovation Challenges” is a difficult read. The reader is set up for 9 challenges, 4 drivers, and 9 ambivalent forces. Then the first challenge covers 6 pages with the one challenge broken into 8 further categories further broken into further lists and individual comments. The current write-up of this first challenge is unexpected per the promise of parsimony, very weighty, and becomes immediately discouraging per the reader thinking every section is going to be broken apart this extensively (which they are not). I would encourage re-writing this section as an overview of this challenge (try to keep it to 2 pages) and then put this highly detailed text into a supplemental document dedicated to fully unwrapping this driver.

Revision: I have a similar reaction for section 1.2. I would break these challenges down into further segments. Instead, craft an overview and put into its own supplemental section.

Minor Revision: For the quotes, it’s occasionally difficult to tell where one stops and another begins. Please add a demarcation such as “Quote 1:…” to clarify.

Minor Revision: For some quotes, you include a description of speaker’s role which is helpful for context. Please include the role for all quotes.

Revision: While I like their inclusion, there are a lot of quotes included in the piece. I would consider continue to edit out some (limit to 2 per discussion point). My key recommendation is to target the quotes that are easiest to read both from a grammatical standpoint and don’t require significant additional context to understand. I find that some of the quotes are difficult to read without having to go over them multiple times. This makes for more frustration as a reader then benefit.

Minor Revision: Please include definitions of “internal” and “external” as they are referred to throughout the paper. Internal is referred to as the “IT solution itself”, but does that include the implementation team, the healthcare organization’s administrators, the providers, and/or extend to the patients?

Revision: I would encourage the authors to potentially bring in a copy editor to tighten up the language. At times, the writing feels too loose/first draft for an academic paper. Particularly given the length of the paper, every sentence needs to be as efficient as possible. For example, on page 26:

Current version: “With minor exceptions, the discussion remained at a highly specific level – as frustration with specific people or organisations rather than as something that might be driven by the structure everybody was working in. At a most fundamental level, issues included:”

Edited version: “With minor exceptions, the discussion focused on frustrations with specific people and organizations rather than the structure of the healthcare system. Issues included:”

Revision: As the results section includes the quotes, often refers to what was “discussed”, and focusing so specifically on the EPaCCS project, it does not read as if this content also is derived from the literature reviews or a broader set of projects. I would include quotes and references to the literature reviews throughout the Discussion section.

Recommendation: I’m curious to learn what the patients/carers thought of as key drivers and challenges to HEI given, they often are a step removed from the data sharing process and/or implementation projects. Did their opinions significantly differ from the staffs? As the patient/caregiver experience is growing as an organizational priority in many healthcare centers, I would recommend a call-out box in either the results or discussion section to report on the patient/caregivers specifically.

Discussion

Overall: I also find the discussion section to be difficult and frustrating to read. The Discussion section currently reads as if someone is speaking to me about their thoughts on the material more casually. For instance, there’s lots of extra phrases such as “Very importantly”, “for now”, “however”, “thus”. There are sentences that read as highly complex, but I believe end up clouding the major point (example on page 61 “It is a genuine transformation of the object of study in the process of it being studied.”) There’s also strong use of the passive voice. This study is full of great material and I want the discussion section to fully serve the reader with very clear and crisp conclusions. I would recommend a strong copyediting with the goal to reduce the number of long lists, use of intermittent phrases, and simplification of sentences to help clarify the major points.

Minor revision: Add the word “were” to sentence on page 51. “The framework was derived from a rich dataset, consisting of in-depth interviews with 45 maximum variation participants, many of whom [were] “key informants”.

Revision: Another example where the writing of the paper needs editing is on page 51.

Current version: “Findings from the interviews were also mapped against findings from four literature reviews on health information technology and health information exchange, covering 135 studies (the inclusion of one and the same study across reviews was explored and the number of unique studies is likely to be lower; yet a level of originality of interpretation is expected to characterise each review.)

Comments: The second half of the sentence in the parathesis doesn’t make sense to me as written and also appears to be a level of detail that feels out-of-place in a summary paragraph that opens the Discussion section.

Revision: I don’t support inclusion of the paragraph on page 53 about the findings indicating that EPaCCS needs to be phased out. First, the conclusion does not fit the aim of the paper. Further, it feels like a sizable leap to assess interviews that are 8-10 years old and come to such a sizable recommendation without the consideration of more recent data. It may be a true or obvious statement for those actively working on EPaCCS, but as a reader without any further context, such a statement does not build confidence in the author’s remarks.

Revision: On page 55, I appreciate the acknowledgement of the study representing a singular case study. Two items to address though are the literature review helped to mitigate that narrowness by bringing in content from many other studies. Could you call that out as a strength? I would also include a comment on the paper’s strength and/or limitations in regards to inclusiveness? Were the people interviewed and organizations they were affiliated with a generalizable sample of the healthcare system? If not, what other groups and/or organizations would you recommend be studied to help add to your findings?

Recommendation: On page 56, I appreciate the write-up on limitations of the study data based on when the interviews were conducted.

Thank you again for the opportunity to review your paper. I learned a lot from and hope my review is helpful.
