# Supplementary material for: From “wading through treacle” to “making haste slowly”: A comprehensive yet parsimonious model of drivers and challenges to implementing patient data sharing projects based on an EPaCCS evaluation and four pre-existing literature reviews
Source: PLOS Digit Health. 2024 Apr 1;3(4):e0000470. doi: 10.1371/journal.pdig.0000470 (PMC10984410; doi:10.1371/journal.pdig.0000470)
Supplement: Attachment — Submitted filename: Response_to_reviewers_14Dec23.docx [file pdig.0000470.s009.docx]

PDIG-D-21-00135R2
From “wading through treacle” to “making haste, slowly” in patient data sharing:
A comprehensive yet parsimonious model of drivers and challenges to data sharing based on an EPaCCS evaluation and four pre-existing literature reviews
PLOS Digital Health

Dear Dr Miguel Ángel Armengol de la Hoz,

Thank you for overseeing such thorough and helpful reviewer feedback. We have taken the comments very seriously and radically revised the paper. See our point-by-point responses in the table below. We hope we have addressed the reviewers’ concerns adequately and will be looking forward to a hopefully final decision in due course.

With very best wishes,

Dr Mila Petrova and Professor Stephen Barclay

| **No** | **Reviewer’s comment** | **Authors’ response** |
| --- | --- | --- |
| **Reviewer 4** | | |
| 1. | Thank you for the opportunity to review your work. I think the paper addresses an important topic and is commendable for its work to bring organization and clarity to extremely difficult to define concepts and observations. In particular, the findings resonated with my own lived experiences in attempting to innovate healthcare systems and I do believe your research advances the field of health information exchange. My key takeaway is this paper is answering in detail what it is that makes the actual implementation and use of these highly promising tools so challenging. | Thank you for this reassuring feedback. It is really valuable for us to hear that the conceptualisation resonates with practitioners who work on innovation implementation. |
| 2. | While the findings in this paper are worthy of publication, I do express similar concerns as the previous reviewer. I found the paper’s writing or communication style needs to continue to be edited for clarity and further polish, particularly in the Results and Discussion section. I hope my comments and feedback below will help to support the further advancement of this paper. | Thank you for yet another stimulus to improve the clarity of the paper further. We have made one of its most significant revisions so far. The reviewing tool identified over 1100 revisions, of which 555 deletions and 488 insertions. We provide further detail below. |
| 3. | Paper Title and Description – A recommendation to consider is to shift the framing of this paper from an HIE paper to be about the incentives and challenges of digital health innovation more generally (utilizing as EPaCCS as the case study/source materials). Based on the title and “Aims” statement, I expected the paper’s content to be more on the technical specifics of data-sharing itself and adjustments of practice operations to incorporate new data sources into the day-to-day workflows. As currently described in the title, this paper will likely attract the attention of technical HIE experts who are seeking that highly tactical guidance. I would consider ways to ensure the title and abstract contain key words (implementation science, project management, healthcare innovation, digital health, etc.) that will draw the attention of a broader audience of healthcare innovators and digital health leaders – those who would benefit the most from the studies findings. | Thank you for this suggestion and highlighting the likelihood that the paper creates expectations of a technical HIE paper offering highly tactical guidance.  We added ‘implementation’ to the title. We refrained from broadening to digital health innovation for word count reasons, as we would have wanted to keep the data sharing aspect in the title too.  We also added the following paragraph early on to clarify the nature of the paper and its intended audience:  *As the case study project involved primarily adapting pre-existing functionalities of locally used clinical IT systems – selecting and harnessing relevant elements in innovative ways as opposed to designing a solution “de novo” – the paper is more relevant to project managers, policy makers, clinicians and other implementers and early adopters as opposed to IT experts interested in high-level technological innovation.* |
| 4. | Author Summary – I find the Author Summary clear, compelling, and concise. | Thank you for the positive feedback. We made some further minor revisions for clarity and hope that they have not changed this perception. |
| 5. | Minor Revision: Please add a source to the sentence “ the evidence in their favour is uncertain.” | We believe that the Author Summary should not contain references as per the journal style. We will request the editorial team’s advice. The relevant references are in the introduction. |
| 6. | Introduction  Comment: I find the Introduction clear and well-written. | Thank you. |
| 7. | Recommendation: Consider retitling this section “Background” as oppose to “Introduction”. Per the level of detail given on HIE, NHS, and EPaCCS given, the word “Background” helps indicate to the reader that we are out of overview descriptions and getting into details. This is a personal preference though. | We changed to Background, as suggested, thank you. |
| 8. | Minor Revision: Please add a source to statement “which are only partially supported by inconclusive and generally low-quality evidence”. | This was our claim, which we had then illustrated with over 35 references on the basis of which it was made. We have revised the claim slightly to make it more neutral:  *Multiple benefits of data sharing have been asserted and partially supported by evidence: …* |
| 9. | Minor Revision: You have a wonderful aim statement written within the introduction: “We also had a strong applied goal: to derive specific and non-trivial recommendations for advancing data sharing projects, particularly ones which are in relatively early stage of their development and implementation.” I would recommend working this language into your Aims statement within the Abstract. I think it is an important goal that will help attract the readers who will most benefit from this paper. | Thank you so much for this helpful suggestion. The statement has now been weaved into the Abstract. |
| 10. | Minor Revision: Please relocate the paragraph labeled “Context of the broader study and service development project” to the Introduction section directly after the paragraph describing NHS. I think the description of EPaCCS better lends itself to the Introduction section, would flow nicely after the description of NHS, and creates a more organic ending to this section of the paper. | Thank you for the suggestion. We see the logic and agree with it but have now shortened the paragraph in question and deleted the subheading of “Context of the broader study and service development project”. In its current briefer version, we believe that the section works better under Methods, but are happy to reconsider. |
| 11. | Minor Revision: Please give 1-2 paragraphs to describe the type of patient data shared via EPaCCS and across the number and type of healthcare organizations involved (home health nursing, etc.). Also any known successes/outcomes of EPaCCS for advancing care. How much data is regularly moving across the system and/or how often is it accessed by providers? Specifically, this would be a good point to describe the template that is later referred to in the paper. Noting this information may be available in S1, the likelihood of a reader stopping to review that document is slim and such information at the outset will help to provide the additional context needed for understanding the results. | We considered this proposal carefully but ultimately decided against it. Since the paper has consistently attracted criticisms of its length, including in this round of reviews, we cut it extensively, yet we also needed to add new text to respond to reviewers’ concerns. We have published (and referenced here) other papers where this EPaCCS-specific information is available.  We have, in fact, recently reduced some of the EPaCCS-related detail. An earlier reviewer criticised the paper for providing high level of detail that was irrelevant to EPaCCS. They were of the impression that this was an EPaCCS-focused paper. We reduced the EPaCCS-detail and clarified that while the case study is of an EPaCCS project, we are aiming to inform conceptualisations of data sharing more broadly. |
| 12. | Materials and Methods  Revision: My major concern is the 8 - 10 year timelapse of the data collected that provides the foundation of the findings for this paper. As a reader, upon learning the age of the dataset, one immediately starts to discount the potential relevance of the findings given a) the advancements in data sharing technology b) the increase user savviness with technology and b) the massive shifts in digital health fueled by the pandemic. Please include a justification (maybe in a text box cutout) to give more rational for use of this dataset. Why this dataset as oppose to conducting a new round of interviews? What indicators do you have that the dataset is still relevant to today? My suspicion is that this is an extremely high quality/hard won dataset that had more to give and deserved to be further analyzed despite its age. The reader needs more justification here than solely acknowledgement of the datasets age in order to continue reading with the confidence that there are valuable findings to come. | We fully share the concern. We had analysed the data in 2016-2017. We did a further radical reconceptualization in 2019 (reflected as stage 4 of the analysis here), after three journal rejections. The paper has been consistently in the peer review pipeline since 2019.  We have reflected timelines better in the paper.  We have also clarified in the discussion that the level of generality at which we consider findings makes them more enduring. We have made clearer suggestions under Implications for Research on what the effects of the datedness might be.  We too would have wanted to collect new data and make a comparison, but this project has long exhausted its funding.  Here is the current version of the paragraphs which discuss the datedness in most detail:  *By now, the data of the study are dated, even if the framework we propose is at a level of generality where most issues are persistent, recurrent and will remain so for decades to come. The big picture we have drawn misses potentially crucial new elements, such as the opportunities for data sharing which developments in AI may be offering; the impact of more recent legislation, such as the GDPR; new expectations for interoperability [72, 73] and, crucially, the effects on digital health of the COVID-19 pandemic. Such changes may have, for instance, substantially reduced the number of “pure challenges” we formulated and this needs to be explored. The framework needs further conceptual specification, including through case studies from other clinical domains. Its practical utility also needs testing and evaluation. Whether our proposal has the potential to grow into a credible standalone implementation framework, including beyond data sharing, or can only serve to support other frameworks is, ultimately, an empirical question.*  And later on:  *As noted on several occasions and discussed in more detail under Implications for Research, the datedness of the data is also a key study weakness. The data (both interviews and literature reviews) were analysed in 2016-2017, with further conceptual analysis conducted in 2019. Since then, the paper has travelled the peer review system of several journals. It was beyond the capacity of the authors and the funding available to them to strengthen the study through the analysis of more recent data. Nonetheless, this was a rich dataset from a sensitive period in the development of data sharing solutions, complemented by literature reviews spanning publications from over two decades. Conceptual frameworks are also less prone to the detrimental effects of time and, if good enough, will both withstand new empirical tests and allow for further development and refinement.* |
| 13. | Revision: You write “We acknowledge the length of time which has elapsed since data collection and have deprioritized the reporting of dated issues (e.g. claims around the need for a “culture change”, which the COVID-pandemic achieved).” Per reproducibility of this research and to build confidence in the dataset, can you provide more detail on how dated issues were identified and eliminated from the dataset? | We have now added the following statement:  *We are not reporting on dated issues, such as: challenges around local organisational entities which no longer exist (e.g. Local Commissioning Groups); claims for the need of a “culture change”, with the latter achieved by the COVID-pandemic; IT-specific challenges which are likely to have been resolved since data collection due to the level of infiltration of technology in everyday life and COVID-related digital gains (e.g. lack of basic IT skills in some staff groups); and claims associated with events which had prominence in the then social discourse but have now faded away (e.g. the Liverpool Care Pathway scandal in palliative and end of life care).* |
| 14. | Minor Revision: Please provide more detail on the interview time period and the stage in the implementation at which the interviews were conducted. Per the aim to support projects in their early development, it would be helpful to know if the interviewees are responding based on the initial implementation time period or several years into a fully mature project? Also, if the interviews were spread out over a long period of time, were interviewees responding based on their experiences at different points in the work. | Thank you for this comment. We have now added the following clarification:  *Interviews were conducted by MP between October 2013 and October 2015. Data were collected over four periods, reflecting distinct stages of the service development project:*   - *initial development, when the project tools and systems were still a work in progress: Oct 2013 – Mar 2014, 10 interviewees, all of whom project team and steering group members.* - *several months after the formal launch of the project (Apr 2014) when GP and specialist services uptake was expected: Aug – Nov 2014, 13 interviewees, 12 of whom GPs and palliative care specialists.* - *stage at which patients and carers were expected to have experienced care involving the sharing of data: Dec 2014 – Apr 2015, 10 interviewees, 9 of whom patients and carers.* - *stage at which the urgent and emergency services were expected to have integrated the data sharing tools into their workflows: Jun 2015 – Oct 2015, 10 interviewees, 7 of whom working in urgent and emergency services.*   *We did not observe stage-related differences of perceptions of challenges and drivers. Further detail on interviewees whose roles did not fit the stage descriptions (5) or were interviewed outside of the above campaigns (1) can be found in S1.* |
| 15. | Minor Revision: Were all the interviews conducted in English? Do you have any demographic data of the interviewees themselves (particularly the patients/carers)? Per the increased prioritization of Diversity, Equity, and Inclusion (DEI) across healthcare, it is helpful to know if this data set represents a broad or more niche segment of healthcare. If highly niche (a healthcare organization that serves primarily high-income individuals or primarily non-English speaking patients), then I would address the need to gain perspectives from a wider variety of organizations in the limitations section of the paper as well. | Thank you. A claim to this effect has now been added to the limitations (we only used the UK convention to abbreviate to EDI).  *We achieved the maximum variation we aimed for in terms of roles; contexts (including of healthcare settings and organisations but also of rurality and deprivation); clinical IT systems; types of use of the data sharing tools; background and levels of experience of health professionals; age groups, gender, conditions and type of relationship for patient and carers, etc. (see Table 1). However, we did not have as an explicit aim to achieve maximum variation in terms of aspects of Equality, Diversity and Inclusion (EDI) such as race and ethnicity, gender non-conformity, or sexual orientation. The study was set up in 2013, when such parameters of diversity could be overlooked in favour of maximising variability along other parameters.* |
| 16. | Revision: Please provide more detail on how the literature reviews were selected, the publication dates of the literature reviews themselves, and the range of publication dates of the 135 source studies reviewed. As a reader, I’m curious to know if the literature reviews cover material written in and around the interview time period or if they cover more recent literature between 2015-2023. If more recent literature is included in the literature review, I would include a note that the literature review was conducted to ensure the applicability of the interview content and further strengthen the relevancy of this paper. If the literature review is only up to 2015, I would provide justification for this decision. | Thank you for this comment. We have now expanded the section as follows:  *Data from four literature reviews on the implementation, uptake (adoption, usage) and effectiveness of health IT were used to query, expand and strengthen the knowledge gained through the analysis of primary data. Two of the reviews – the systematic reviews of Rudin et al. (2014) [33] and Eden et al. (2016) [38] – were on health information exchange (HIE), respectively of its usage and effects [33] and barriers and facilitators [38]. The two other reviews – of Rippen et al. (2013) [65] and Cresswell and Sheikh (2013) [66] – were on health information technology. The former was a “targeted review” which offered an “organizational framework for health information technology”. The latter was an “interpretive review” on “organizational issues in the implementation and adoption of health information technology innovations”. The literature reviews were selected as the most relevant and detailed reviews from the authors’* *collection of HIE and HIT papers at the time and extensive, but not systematic, literature searches on HIE. The total set from which the four reviews were chosen was of over 250 records, either previously saved or kept post-screening of the HIE search results. We did not have the capacity to conduct an accompanying systematic review.*  *The reviews covered 135 source studies. The publication years for the included studies were: 1990-2015 (Eden et al.), 1997-2010 for HIT implementation literature and 1983-2010 for health IT related theories (Rippen et al.), 1997-2010 (Cresswell et al.) and 2003-2014 (Rudin et al.). Both the text of the literature reviews and tables representing source studies were coded line-by-line.* |
| 17. | Revision: Please provide the time period when the interview analysis and synthesis of interview and literature review data was conducted (page 12). | Done, thank you. |
| 18. | Results  Overall Comment: The results section is where the paper’s writing - at times - degrades in quality, efficiency of language, clarity, and structure. I would encourage that the author’s keep editing this section to ensure the writing is as solid as the two previous sections. Further, I would rethink the aim of this section from covering every detail uncovered in the research to instead focus on how to “teach” the reader the overview of each challenge/driver/ambivalent force. For the details, I would shift the text to supplemental documents when needed to provide immense detail on a framework component. I recommend this approach in order to make a more manageable and approachable paper for a reader to cover in one-sitting. The additional materials are there for the person who needs to study the content at its most nuanced level. | Thank you for this suggestion. We have revised this part of the paper radically, both in terms of structure and language. We have also reduced significantly the level of detail and, indeed, shifted much of it to boxes and appendices. |
| 19. | Recommendation: I would consider listing the “pure drivers” before the “pure challenges” in the paper. This order better matches the flow of an implementation project as the incentives for conducting such work come before the challenges reveal themselves during the actual implementation. This is a personal preference though per how my brain is wired to order drivers vs. challenges. | Thank you for this suggestion. We considered it seriously, not least because the challenges are closer in nature to the ambivalent forces and thus, logically, it might have been better if the two higher-level categories were side-by-side. We did, however, retain the current order as the subsection on challenges is richer, its findings are more unexpected, and we feel it takes more attention to read. The drivers are easier to skim through. For a long paper, we wanted to benefit from the sharper early attention of readers and also set the inner expectations for a paper that requires some thinking through. |
| 20. | Revision: Given the overall length of the “results” section, I would include a short bullet point list or a table listing out the titles of each of the framework components and potential a 1-sentence description of each component. This addition will help prepare the reader for the content to come and also give them a chance to skip forward to the section of most relevance if needed. | Thank you. We have now added such anticipatory sections. |
| 21. | Revision: The section 1.1 “Radical Innovation Challenges” is a difficult read. The reader is set up for 9 challenges, 4 drivers, and 9 ambivalent forces. Then the first challenge covers 6 pages with the one challenge broken into 8 further categories further broken into further lists and individual comments. The current write-up of this first challenge is unexpected per the promise of parsimony, very weighty, and becomes immediately discouraging per the reader thinking every section is going to be broken apart this extensively (which they are not). I would encourage re-writing this section as an overview of this challenge (try to keep it to 2 pages) and then put this highly detailed text into a supplemental document dedicated to fully unwrapping this driver. | Thank you for this recommendation. We have reduced this section from 2099 words, including quotes, to 934 words. |
| 22. | Revision: I have a similar reaction for section 1.2. I would break these challenges down into further segments. Instead, craft an overview and put into it’s own supplemental section. | Thank you for the specificity once again. We have now reduced this section from 1076 words, including quotes, to 408. |
| 23. | Minor Revision: For the quotes, it’s occasionally difficult to tell where one stops and another begins. Please add a demarcation such as “Quote 1:…” to clarify. | Wе have now moved key quotes to Boxes within the manuscript and have demarcated them as separate by consistently indicating a source. |
| 24. | Minor Revision: For some quotes, you include a description of speaker’s role which is helpful for context. Please include the role for all quotes. | The reason for the discrepancy (and we had mentioned it previously but perhaps not clearly enough) is that some roles make a person uniquely identifiable in the locality of the study. The decision was an aspect of protecting confidentiality. |
| 25. | Revision: While I like their inclusion, there are a lot of quotes included in the piece. I would consider continue to edit out some (limit to 2 per discussion point). My key recommendation is to target the quotes that are easiest to read both from a grammatical standpoint and don’t require significant additional context to understand. I find that some of the quotes are difficult to read without having to go over them multiple times. This makes for more frustration as a reader then benefit. | Thank you for this recommendation. It was very helpful for our decision to move all quotes into boxes and make their reading more optional. |
| 26. | Minor Revision: Please include definitions of “internal” and “external” as they are referred to throughout the paper. Internal is referred to as the “IT solution itself”, but does that include the implementation team, the healthcare organization’s administrators, the providers, and/or extend to the patients? | Thank you for this comment. We have now clarified as follows:  *The 4-component framework of pure drivers we propose is defined in terms of whether the driver is internal or external to the data sharing solution and the team developing and implementing it. The degree of internality/ externality is associated with a greater or lesser control of the project team over a driver as well as greater or lesser importance of the quality of the IT solution for its broad uptake. “Internal” in relation to the IT solution was conceptualised as pertaining not only to its functionalities, user interface and technical specifications, but also to its clinical domain and goals (what you can do and achieve with it). “Internal” in relation to the project team concerned its leadership (a project manager and a clinical lead) and core team members (further five of them).* |
| 27. | Revision: I would encourage the authors to potentially bring in a copy editor to tighten up the language. At times, the writing feels too loose/first draft for an academic paper. Particularly given the length of the paper, every sentence needs to be as efficient as possible. For example on page 26:  Current version: “With minor exceptions, the discussion remained at a highly specific level – as frustration with specific people or organisations rather than as something that might be driven by the structure everybody was working in. At a most fundamental level, issues included:”  Edited version: “With minor exceptions, the discussion focused on frustrations with specific people and organizations rather than the structure of the healthcare system. Issues included:” | We hope that our edits have changed this perception. Every sentence in the previous version was dozens of times away from a first draft. Ironically, thinking each word through and aiming for maximum precision seem to have created too often the impression that the language is too complex and unclear. We hope that by losing a lot of detail we have made the paper more reader friendly.  As far as the specific sentence you mentioned, this paragraph was now extensively edited to become:  *With minor exceptions, the discussion of this subtype of challenge remained at a highly specific level. Interviewees expressed frustration with specific people or organisations, without considering the possibility that these were presenting as “difficult” because of deep-seated, structural problems of interaction amongst elements of the health system. Issues discussed most often included: “politics”; the importance of watching out for the interests of one’s own team and organisation; the lack of action to match promises given; and differences in language. The language of IT was, expectedly, perceived as a particular challenge.* |
| 28. | Revision: As the results section includes the quotes, often refers to what was “discussed”, and focusing so specifically on the EPaCCS project, it does not read as if this content also is derived from the literature reviews or a broader set of projects. I would include quotes and references to the literature reviews throughout the Discussion section. | As mentioned, we have now decided to move key quotes into boxes within the manuscript, with the remainder in S2 (Further quotes).  Overall, the quotes from the primary data were much richer than those from the literature reviews. The reviews were abstracting and synthesising findings, so the level of detail, nuance and depth was noticeably lower. The review findings ended up serving a confirmatory and supporting function. As the reviews are published and available in their entirety, we did not feel it was particularly helpful to include elements of them in our quotes appendix, which also serves as a way to share our primary data.  We provide further detail under point 59 below. |
| 29. | Recommendation: I’m curious to learn what the patients/carers thought of as key drivers and challenges to HEI given they often are a step removed from the data sharing process and/or implementation projects. Did their opinions significantly differ from the staffs? As the patient/caregiver experience is growing as an organizational priority in many healthcare centers, I would recommend a call-out box in either the results or discussion section to report on the patient/caregivers specifically. | The previous version of the paper had this note in Table 1:  *Recruitment of patients and carers was discontinued after having interviewed 9 in 5 interviews (original intention was for 24 participants, single person interviews). Data sharing was showing to be a minor and vague consideration relative to their broader experience of illness, healthcare in general and end of life care in particular, with strongly positive opinions expressed in its favour.*  We considered whether to include a callout box, as you suggested, but since we already have 8 boxes, some of them 2 pages long, 1 table and 4 really lengthy appendices, we decided not to go beyond this mention in the table. |
| 30. | Discussion  Overall: I also find the discussion section to be difficult and frustrating to read. The Discussion section currently reads as if someone is speaking to me about their thoughts on the material more casually. For instance, there’s lots of extra phrases such as “Very importantly”, “for now”, “however”, “thus”. There’s sentences that read as highly complex, but I believe end up clouding the major point (example on page 61 “It is a genuine transformation of the object of study in the process of it being studied.”) There’s also strong use of the passive voice. This study is full of great material and I want the discussion section to fully serve the reader with very clear and crisp conclusions. I would recommend a strong copyediting with the goal to reduce the number of long lists, use of intermittent phrases, and simplification of sentences to help clarify the major points. | We are sorry to hear that the previous version was so frustrating to read. We have radically revised the Discussion and hope it is now clearer, with key points that are “clear and crisp”, as recommended. |
| 31. | Minor revision: Add the word “were” to sentence on page 51. “The framework was derived from a rich dataset, consisting of in-depth interviews with 45 maximum variation participants, many of whom [were] “key informants”. | Thank you for the point. The “key informant” element has now been deleted. We didn’t feel it was crucial and also begged the question of how a “key informant” was defined. |
| 32. | Revision: Another example where the writing of the paper needs editing is on page 51.  Current version: “Findings from the interviews were also mapped against findings from four literature reviews on health information technology and health information exchange, covering 135 studies (the inclusion of one and the same study across reviews was explored and the number of unique studies is likely to be lower; yet a level of originality of interpretation is expected to characterise each review.)  Comments: The second half of the sentence in the parathesis doesn’t make sense to me as written and also appears to be a level of detail that feels out-of-place in a summary paragraph that opens the Discussion section. | Thank you for this point. It is, indeed, a comment more suitable for a methods section. Contents in parenthesis deleted. |
| 33. | Revision: I don’t support inclusion of the paragraph on page 53 about the findings indicating that EPaCCS needs to be phased out. First, the conclusion does not fit the aim of the paper. Further, it feels like a sizable leap to assess interviews that are 8-10 years old and come to such a sizable recommendation without the consideration of more recent data. It may be a true or obvious statement for those actively working on EPaCCS, but as a reader without any further context, such a statement does not build confidence in the author’s remarks. | The paragraph has now been deleted. You were right that we have perhaps added too much of our broader knowledge of EPaCCS, which has not been reflected in this paper. |
| 34. | Revision: On page 55, I appreciate the acknowledgement of the study representing a singular case study. Two items to address though are the literature review helped to mitigate that narrowness by bringing in content from many other studies. Could you call that out as a strength? I would also include a comment on the paper’s strength and/or limitations in regards to inclusiveness? Were the people interviewed and organizations they were affiliated with a generalizable sample of the healthcare system? If not, what other groups and/or organizations would you recommend be studied to help add to your findings? | Thank you for both comments. We have now added the following points to the strengths and limitations section:  *This was, however, a single case study in a single locality in England, concerning a particular type of data sharing – data sharing for individual care at the end of life. Some of its findings are unlikely to be generalisable, even if we aimed to increase generalisability through including data from pre-existing literature reviews. As the depth and richness of the interview data was much higher than that of the reviews, the primary data ended up retaining a dominant role.*  And further down:  The study also had both strengths and limitations of sampling:   - We achieved the maximum variation we aimed for in terms of roles; contexts (including of healthcare settings and organisations but also of rurality and deprivation); clinical IT systems; types of use of the data sharing tools; background and levels of experience of health professionals; age groups, gender, conditions and type of relationship for patient and carers, etc. (see Table 1). However, we did not have as an explicit aim to achieve maximum variation in terms of aspects of Equality, Diversity and Inclusion (EDI) such as race and ethnicity, gender non-conformity, or sexual orientation. The study was set up in 2013, when such parameters of diversity could be overlooked in favour of maximising variability along other parameters. - The narrow IT and IG specialists in the sample were six (respectively 4 and 2), even if many participants were clinicians or commissioners with notable interest in health IT. Some of them also had associated formal or informal roles. From the perspective of elucidating IT-specific challenges, the study may not have had enough interviewees of the right technical background. On the other hand, the maximum variation sampling approach, which meant that IT specialists were only one of the subgroups of interest, allowed for a more comprehensive picture of challenges and drivers to emerge. |
| 35. | Recommendation: On page 56, I appreciate the write-up on limitations of the study data based on when the interviews were conducted. | Thank you. We have now moved the detailed description of the limitations in terms of datedness to the Implications for research section so as to expand it with further recommendations. None of the contents was lost though. |
| 36. | Thank you again for the opportunity to review your paper. I learned a lot from and hope my review is helpful. | Thank you for the close and careful reading of our paper and for all the insightful recommendations. We hope we’ve managed to address them effectively. We definitely feel that the paper has improved as a result. |
|  |  |  |
| **Reviewer 5** | | |
| 37. | Reviewer #5: This is an interesting paper which indicates the need for data sharing projects to focus less on refining their IT tools and more on shaping the relational and structural contextual parameters in the midst of which they are implemented. | Thank you for taking the time to review our paper. We are pleased you found it interesting. |
|  |  |  |
| **Reviewer 6** | | |
| 38. | This manuscript proposed a model with >500 challenges and >300 drivers for implementing Electronic Palliative Care Coordination System (EPaCCS) and other data sharing projects. Also, authors have concluded that one should focus less on refining their IT tools and more on shaping the social interactions and structural and contextual parameters for data collection.  I would like to summarize my comments in few points as: | Thank you for taking the time to review our paper. |
| 39. | 1. Entire manuscript is written in a very subjective way or “narrative style” for data collection process, still reader will not find any glimpse of data, or any data processing methods, and outcome in table or figure. | Thank you for this comment. It made us realise that perhaps we have not clarified sufficiently the type of paper we had written. We fear that, unintentionally, the opening paragraphs of the paper have created expectations that this paper would be about technicalities of data sharing or about its effectiveness. We have now added this paragraph, also in response to comments made by Reviewer 4 (see point 3 above):  *As the case study project involved primarily adapting pre-existing functionalities of locally used clinical IT systems – selecting and harnessing relevant elements in innovative ways as opposed to designing a solution de novo – the paper is more relevant to project managers, policy makers, clinicians and other implementers and early adopters as opposed to IT experts interested in high-level technological innovation.* |
| 40. | 2. The primary data source, which is 40 in-depth interviews, approximately 300,000 words is quite ambiguous and not suffice to make any conclusion. | Relative to the goals of the paper and the type of study, this was a rich and highly informative dataset, which we have also expanded with findings from 4 literature reviews. Our goal was not to explore if EPaCCS are effective or not. We aimed to explore the perceived challenges and drivers for their development and implementation and organise them in a conceptual framework. |
| 41. | 3. In this modern era, where IoT devices are gaining enormous popularity, in my opinion (unlike the authors) one should give more importance to data collection mechanisms, devices, and IT tools for seamless, accurate and precise data collection. Also, nowadays remote, contactless methods of data collection are easier, cheaper, convenient, etc., so social interactions and other things are becoming secondary. | We too were very surprised by the finding that three-quarters of the drivers and challenges identified were unrelated to the IT solution. This is a point entirely grounded in the data.  We have added, however, a statement which makes it clearer that our sample may have affected the nature of the findings (see below). Thank you for helping us recognise this. We also softened some of our original claims.  *Most broadly, this study underscored how important it is for data sharing project teams to look outside of the IT tools they are building and refining. Over three-thirds of the drivers and challenges we identified at the level of the 800-component intermediary framework did not pertain to the IT solution at all. While the nature of the study sample is likely to have affected the ratio observed (users and implementers were more than IT developers), the number and magnitude of non-IT challenges and drivers identified was substantial. This seems to justify a recommendation that teams working on data sharing project need to focus persistently and intentionally on shaping the social interactions and structural and contextual parameters in the midst of which their IT tools are implemented, not only or primarily on perfecting them.* |
| 42. | 4. This manuscript could be written in simpler English, but abundant use of idiom, phrases, and words from classical English literature makes it hard to understand and may be not a good choice for a technical journal article. | Thank you for the recommendation. We have radically revised the language of the manuscript, hopefully making it much simpler and less idiomatic. |
| 43. | Overall, I will not recommend this manuscript. | We appreciate that every type of paper has its readers and we hope to have clarified the type of paper we have aimed to write. Thank you for taking the time to review our paper and to articulate your criticisms. They helped us against overclaiming. |
|  |  |  |
| **Reviewer #7** | | |
| 44. | The authors present a framework including challenges, drivers, oppositional and and ambiguous parameters that were identified while implementing an end-of-life data sharing platform, accompanied with recommendations to move forward. Thematic analysis based on project team interviews yielded an initial framework which was used in subsequent framework analysis with remaining interviewees, using a realist approach. The authors discuss the different parameters in detail, accompanied with quotes from the interviewees. A series of practical and research-related implications are discussed. For instance, the authors propose a larger focus on social interactions and contextual parameters; as well as eliciting assumptions / perspectives from stakeholders as multiple factors can be easily seen as both drivers and challenges ("ambivalent"). | Thank you for the helpful summary. |
| 45. | Overall, I find that this paper has a great potential to contribute to both the practical implementation and academic literature on patient data sharing. It contains many interesting discussions on pros and cons, both expected and unexpected. However, I believe there are serious issues with presentation and lack of literature consideration. Although there are many issues listed, I believe that, once they are fixed, this paper has the potential to be a large contribution to the state of the art. | Thank you for the encouragement. We hope we have addressed your concerns adequately. |
| 46. | (1) To an extent, I agree with reviewer 3's first comment on the paper being "difficult to read". In general, the prose could be made a lot more concise. This seems to be an issue of writing style as it occurs throughout the paper. Note that many researchers are used to scanning papers for parts of interest - this is much more difficult if the text is very narrative and dense. | Thank you for the recommendation. As mentioned in response to other reviewers, we have radically revised the paper, not least by losing much detail and moving quotes to boxes, hopefully making its structure and language far simpler and clearer. |
| 47. | It's hard to pinpoint one representative example, but I make an effort below:  - A small example, but "The above sidelines at least two further dichotomies." One can simply say there are other dichotomies/contrasts - it makes one think why the former "sidelines" the latter (this seems irrelevant), breaking the flow of the text. | Removed, thank you for the specific point. |
| 48. | - "Expectedly, some interviewees saw an issue from the perspective of the problem" I assume the authors mean that some only look at the problems, and others tend to be more positive (?) The latter language is a lot easier to grasp. | Thank you for highlighting. This sentence has now been deleted. |
| 49. | - "One of the richest subtypes of “ambivalent forces” had its roots in differences, at times radical, of perspective, values, mindset, needs and goals, etc. relative to which a factor was considered" A factor could be seen as positive or negative depending on the perspective, thus making it ambivalent (?) | Thank you for highlighting. We have now expanded this section and hope our meaning is clearer but are prepared to revise further if not. The formulation of this subtype is informed by a body of work on Values Based Practice pioneered by KWM Fulford, yet it will only add to the complexity of an already complex paper if we introduce this background.  Here is how we have tried to add some clarity:  *One of the richest subtypes of “ambivalent forces” had its roots in differences of perspective, values, evaluation criteria, views, preferences and similar value-laden phenomena of which most people would argue, from a rational stance, that they vary legitimately from person to person and are a matter of personal choice. Yet most people again, when emotionally involved, would find it hard to countenance values, perspectives, views, etc. which are polar opposite to theirs without experiencing them as wrong or misguided. Such diversity of values and associated conflict was relevant to core features of the EoLC data sharing project.* |
| 50. | - "whether planning future care and consulting existing care plans has sufficient advantages over the immediate, adaptive response to what is, essentially, always a new clinical situation" | Thank you for highlighting. We changed this to:  *For instance, some interviewees argued for the benefits of planning future care and consulting existing care plans, while others prioritised the immediate, adaptive response to the clinical situation at hand (with consequences for whether a care plan was included as a data sharing field or not).* |
| 51. | - "To an extent, such self-reflective comments aimed for a narrative resolution of something that might have required practical solutions" | Section now removed. |
| 52. | Many of the paragraphs are quite interesting but very narrative. I often found myself looking back for the actual main points hidden in the narration, and having to write down major points to keep track. | Wе hope that the significant editing (as mentioned in a previous response, we made over 1100 edits) has made the main points clearer. |
| 53. | In this vein, consider structuring Sections 1.3, 1.4 around the major concepts discussed - they are currently 1 long text, making it difficult to quickly get the main points. Similarly, for each type of ambivalent force, please summarize in the beginning how they pertain to both drivers and challenges - it currently has to be inferred from the text. | We have revised significantly both 1.3 and 1.4. The former was shortened from 890 words, including quotes, to 347 words. The latter was edited further, even if the word count remained comparable. We hope it is now clearer.  We have edited significantly the text on ambivalent forces. |
| 54. | Consider structuring the implications and limitations sections using a list of bullets - I again found myself having to write down major points to keep track. A lot of very useful points are being made but are currently hidden in long narration. | We have re-worked the discussion significantly. We hope that it brings the main points out more clearly. We also included more bulleting, namely in the section outlining the distinctive characteristics of our framework and the limitations of the study. |
| 55. | (2) The authors seem to have already dropped a number of quotes, however, the following are still unclear to me. (I am not advocating their removal but perhaps the authors can you clarify their meaning or relevance.) - Relevance of the quote at the bottom of p. 19 is unclear. - Quote on emergent innovation is unclear. - Under resource limitations - some quotes here are gut wrenching, but their relevance to resource shortage for use of EoLC is not wholly clear. - Clarify that first 4 quotes on p. 27 each separately pertain to points from bulleted list (I think??) - this was quite confusing at first. - On p. 31, the relevance of the second part of the quote is unclear. - The last quote on p. 38 is unclear - what does "only get one chance right at the end" mean? | Thank you for this comment and the specificity of the examples. We opted for moving some key quotes into Boxes (in the manuscript), with the remaining ones transferred into the existing S2 on Further quotes. We tried to select for the Boxes quotes which were clear illustrations of a category needing little context. |
| 56. | (3) Note that a lot of the initial challenges pertain to a lack of user engagement, rather than purely repetiton or a vicious circle: e.g., lack of buy-in from healthcare workers (ambulance service; sticking with letters), and no time set aside for learning. I believe this should be outlined, possibly (but not necessarily) by introducing a separate parameter. | We agree about the user engagement point. In the Discussion, when we are comparing our framework with other implementation frameworks, we clarify why we prefer to focus on form and dynamics as opposed to standard entities (e.g. users). |
| 57. | Importantly, it seems that the authors' recommendations on this topic *sidestep the entire field of change management and implementation science*. E.g., use of the ADKAR model would already help tick a lot of the challenge boxes. This greatly reduces the utility of the recommendations, which currently seem rather ad hoc. | Thank you for prompting us to bring in this literature too. We had made a previous decision not to discuss it, as associated models were part of the literature reviews we used as secondary data. We do, however, see how omitting it leaves the framework we are suggesting decontextualised.  We have now referenced the ADKAR model, as well as four other influential implementation frameworks (see Box 8). We have also added a section on three distinctive features of the framework we propose.    The recommendations were derived from the data, as opposed to being ad hoc, and our experience with the implementation of the project. We have clarified that they need testing.  We also clarified, under “Implications for research”, that we do not see the framework as necessarily a standalone one. |
| 58. | Box 2 and 3 are more interesting, however, consider elaborating on the link between these points and discussions on drivers (e.g., this was clear to me for the "fitting in" recommendation, but not for e.g., "Data sharing projects are not projects", "be gracious when winning"). Similarly, in box 1, the part on "brutal paradox" has a sweeping statement "will thus create at least as many problems as it solves" but does not seem supported by the corresponding section. | Thank you for this comment. We have revised the relevant sections to make them clearer. We have also removed the statement which felt too sweeping. We have not added further contents into the Drivers sections as while we made over 500 deletions throughout the paper, we added about as many “insertions” to respond to reviewers’ recommendations. The information is available in the Appendices and we have pointed towards the latter. |
| 59. | (4) It's a bit unclear to me how the existing literature reviews were used to concretely inform the study, aside from the evaluation setup mentioning that the analysis framework was "expanded through coding the contents of the literature reviews". Please consider linking the parameters to the literature, or including this coding as supplementary information. | Thank you for this comment. We have now added a further point of clarification to the Methods, that “Both the text of the literature reviews and tables representing source studies were coded line-by-line” (as opposed to the more vague “analysed”).  In the Limitations, we also added the following statement:  *Some of its findings are unlikely to be generalisable, even if we aimed to increase generalisability through including data from pre-existing literature reviews. As the depth and richness of the interview data was much higher than that of the reviews, the primary data ended up retaining a dominant role.*  Key findings from the literature review can be seen in Appendix 4.  As the coding of the literature reviews was part of the coding under 1,800 parameters, a meaningful sharing of this (where it is visible what comes from the primary data and what comes from the literature reviews) would mean that we generate, organise and potentially share 1,800 NVivo files. We do not believe that the benefits are worth the investment of time.  We have now added this sentence to the Data Statement:  “The authors will also consider sharing further data and coding frameworks upon reasonable request.” |
| 60. | (5) The issues below refer to vague language being used: |  |
| 61. | - "Patient data systems are notoriously difficult to design, implement and sustain." That they certainly are - consider adding a few short reasons why that is for non-initiates. | Thank you for this suggestion. We will need to request further advice from the editorial office. As per the journal requirements, the Author Summary is 150-200 words. We are at 199. |
| 62. | - There is an unclear link between the EoLC data sharing project and the EPaCCS project - was the latter the target of the EoLC, or something separate (the authors mention it is only "under evaluation")? The results of the study mostly mention the EoLC project. | Thank you for highlighting this. We have now edited the section to include a very direct statement that: “The EoLC data sharing project, launched in 2014, has been a leading Electronic Palliative Care Coordination System”. |
| 63. | - "The Cambridgeshire and Peterborough EPaCCS was initiated in 2012, officially launched in 2014 and is currently “business as usual” in some respects and an evolving innovation with uncertain outcomes in others." This sentence is rather meaningless without any idea of what the former (business as usual) and the latter entail. | Thank you for pointing out the vagueness. We agree and have simplified the statement but have refrained from expanding further for word count reasons. |
| 64. | - "deprioritised the reporting of dated issues" - what does deprioritization mean in this setting? | Thank you for this query. We have now clarified as follows:  *We are not reporting on dated issues, such as: challenges around local organisational entities which no longer exist (e.g. Local Commissioning Groups); claims for the need of a “culture change”, with the latter achieved by the COVID-pandemic; IT-specific challenges which are likely to have been resolved since data collection due to the level of infiltration of technology in everyday life and COVID-related digital gains (e.g. lack of basic IT skills in some staff groups); and claims associated with events which had prominence in the then social discourse but have now faded away (e.g. the Liverpool Care Pathway scandal in palliative and end of life care).* |
| 65. | - "Data collection was spread across three campaigns reflecting distinct stages" So were data collected _during_ each separate campaign? | Thank you for the query, which overlaps with a concern of Reviewer 4. We have now expanded this section as follows:  *Interviews were conducted by MP between October 2013 and October 2015. Data were collected over four periods, reflecting distinct stages of the service development project:*   - *initial development, when the project tools and systems were still a work in progress: Oct 2013 – Mar 2014, 10 interviewees, all of whom project team and steering group members.* - *several months after the formal launch of the project (Apr 2014) when GP and specialist services uptake was expected: Aug – Nov 2014, 13 interviewees, 12 of whom GPs and palliative care specialists.* - *stage at which patients and carers were expected to have experienced care involving the sharing of data: Dec 2014 – Apr 2015, 10 interviewees, 9 of whom patients and carers.* - *stage at which the urgent and emergency services were expected to have integrated the data sharing tools into their workflows: Jun 2015 – Oct 2015, 10 interviewees, 7 of whom working in urgent and emergency services.*   *We did not observe stage-related differences of perceptions of challenges and drivers. Further detail on interviewees whose roles did not fit the stage descriptions (5) or were interviewed outside of the above campaigns (1) can be found in S1.* |
| 66. | - In table 1, what is the difference between mid-career and experienced - how are these categories defined? | Thank you for this query. We have now added the details (available from a prior survey of GPs).  0-9 years of experience – early career  10-19 years of experience – mid-career  Over 20 of experience – experienced |
| 67. | - "An extensive thematic analysis was undertaken after the project team interviews" Unsure what role this refers to in table 1; project developers (those were only 6)? | Thank you. We have now used the same vocabulary of “project developers”. |
| 68. | - Under alternatives (challenge): "national data sharing work, such as the Summary Care Record (generic with an EoLC specific module)", "commercial products (generic, some with an EoLC-specific module)", etc. I am unsure what the parts in parenthesis mean. | We have now edited this section and the statements no longer exist. The sentence which aims to clarify the same points is below. We hope it is clearer:  *Between themselves and unprompted by a specific question in the interview schedule, the interviewees identified 13 types of alternatives to the EoLC data sharing tools, mostly EoLC-specific tools (e.g. patient-held “yellow folders” containing EoLC information) or broad-based tools which have modules with EoLC information (e.g. the national Summary Care Record).* |
| 69. | - "the self-centred choices of some patients in a service free at the point of care" Could you give an example? | This was part of a paragraph which was now removed to reduce word count. |
| 70. | - The last bullet on p. 47 is unclear. | Thank you for this comment. We have now edited as follows:  *Conflicts of values also affected the particular type of data sharing advanced. For instance, some interviewees argued for the benefits of planning future care and consulting existing care plans, while others prioritised the immediate, adaptive response to the clinical situation at hand (with consequences for whether a care plan was included as a data sharing field or not). Differences of values and evaluation criteria also affected assessments of more diffuse characteristics of the data sharing project, such as its progress and success or the support structures in which it was embedded or which it had set up.* |
| 71. | - The part on complex entanglements is unclear. | Thank you for highlighting. We have revised this section significantly. |
|  | (6) Miscellaneous: |  |
| 72. | - There is a stark contrast between section 2.2, where the project team is viewed as highly positive, also by external collaborators, and section 1.3, where collaboration between team members was seen as overwhelmingly negative. I really got a mistaken impression from section 1.3 - consider nuancing this section and referring to section 2.2. | Thank you for this comment. We have now added the following clarification to point 1.3.:  *(Note though that this negative perception did not apply to the core project team, see “2.2. Pure drivers internal to the project development and implementation team”.)* |
| 73. | - The authors initially devised a high-order framework on a number of parameters, instead of the presented dyads. However, it is unclear how the former was more contingent on the data compared to the current approach. | Thank you for this query. We were discussing the *prioritisation* of issues as contingent upon the data. The final framework does not include such a prioritisation. We have now clarified as follows:  *As we felt that the above quantitively derived prioritisation was too contingent on our data …* |
| 74. | - Please include short summaries for the remaining parameters (1.1.5 - 1.1.8; 3.6 - 3.9). | Thank you for this suggestion. Following the advice of Reviewer 4, comment 18 (copied), we decided to leave this for the appendices:  *“Further, I would rethink the aim of this section from covering every detail uncovered in the research to instead focus on how to “teach” the reader the overview of each challenge/driver/ambivalent force. For the details, I would shift the text to supplemental documents when needed to provide immense detail on a framework component. I recommend this approach in order to make a more manageable and approachable paper for a reader to cover in one-sitting. The additional materials are there for the person who needs to study the content at its most nuanced level.”* |
| 75. | - "The provision of more informational support to clinicians who were uncertain in their judgement could mean that they experienced greater pressure to make decisions they felt uncomfortable with." Do the authors mean that there is a higher likelihood that decision will ultimately be wrong? Unsure where the "uncomfortable" is coming from. | Thank you for this point. We have now edited to state:  *The provision of more informational support to clinicians who were uncertain in their judgement could mean that they experienced greater pressure to make decisions by themselves. Alternatively, there was a risk that non-specialists became unjustifiably confident in their ability to make decisions requiring expert input.*  Once again, thank you for the thorough review and helpful queries and recommendations. We hope we have addressed them adequately. |
